# Controlling Isomerization of Photoswitches to Modulate 2D Logic‐in‐Memory Devices by Organic–Inorganic Interfacial Strategy

**DOI:** 10.1002/advs.202207443

**Published:** 2023-03-11

**Authors:** Yongli Duan, Miaomiao Song, Fanxi Sun, Yi Xu, Fanfan Shi, Hong Wang, Yonghao Zheng, Chao He, Xilin Liu, Chen Wei, Xu Deng, Longquan Chen, Fucai Liu, Dongsheng Wang

**Affiliations:** ^1^ School of Optoelectronic Science and Engineering University of Electronic Science and Technology of China Chengdu 610054 P. R. China; ^2^ School of Physics University of Electronic Science and Technology of China Chengdu 610054 P. R. China; ^3^ Department of Physics University of Science and Technology of China Hefei 230026 P. R. China; ^4^ Department of Orthopedic Sichuan Provincial People's Hospital and Sichuan Academy of Medical Science and Affiliated Hospital of University of Electronic Science and Technology of China Chengdu 610072 P. R. China; ^5^ College of Polymer Science and Engineering Sichuan University Chengdu 610065 P. R. China; ^6^ Institute of Fundamental and Frontier Science University of Electronic Science and Technology of China Chengdu 610054 P. R. China

**Keywords:** 2D materials, donor–acceptor Stenhouse adducts, logic‐in‐memory devices, photoisomerization, photoswitches

## Abstract

Logic‐in‐memory devices are a promising and powerful approach to realize data processing and storage driven by electrical bias. Here, an innovative strategy is reported to achieve the multistage photomodulation of 2D logic‐in‐memory devices, which is realized by controlling the photoisomerization of donor–acceptor Stenhouse adducts (DASAs) on the surface of graphene. Alkyl chains with various carbon spacer lengths (*n* = 1, 5, 11, and 17) are introduced onto DASAs to optimize the organic–inorganic interfaces: 1) Prolonging the carbon spacers weakens the intermolecular aggregation and promotes isomerization in the solid state. 2) Too long alkyl chains induce crystallization on the surface and hinder the photoisomerization. Density functional theory calculation indicates that the photoisomerization of DASAs on the graphene surface is thermodynamically promoted by increasing the carbon spacer lengths. The 2D logic‐in‐memory devices are fabricated by assembling DASAs onto the surface. Green light irradiation increases the drain–source current (*I*_ds_) of the devices, while heat triggers a reversed transfer. The multistage photomodulation is achieved by well‐controlling the irradiation time and intensity. The strategy based on the dynamic control of 2D electronics by light integrates molecular programmability into the next generation of nanoelectronics.

## Graphical abstract

Controlling the photoisomerization of donor–acceptor Stenhouse adducts (DASAs) on the surface of graphene is reported here. Carbon spacers with various lengths are introduced onto DASAs to generate tunable organic–inorganic interfaces. The microstructure and mechanical properties on the surface are demonstrated to closely affect the photoisomerization. Multistage photomodulation of 2D logic‐in‐memory devices are further achieved by green light.

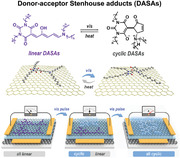

## 1 Introduction

The recent breakthroughs in information technology such as machine learning, Internet of Things, and data‐intensive applications require both high‐performance and energy‐efficient computing.^[^ ^1^ ^]^ However, the processing and storage units are separated in the von Neuman computing architecture, which hinders the ability of standard processors to meet the requirements of the above‐mentioned Big data applications.^[^ ^2^ ^]^ Therefore, approaches to bridge the existing gap between memory and logic units have been attracting researchers’ interest.^[^ ^2a^ ^]^ Logic‐in‐memory was considered as an ideal hardware architecture, as the logic operation and data storage are integrated on the same device.^[^ ^2^, ^3^ ^]^ The success of logic‐in‐memory devices strongly depends on the materials chosen and devices operation mechanism. 2D materials, such as graphene and transition metal dichalcogenides (TMDs), exhibit predominant quantum effects and fascinating electronic and optical properties, which have been extensively investigated in logic‐in‐memory devices.^[^ ^4^ ^]^ Moreover, due to the atomically flat surface, 2D materials show great potential to be integrated with versatile organic materials and could be controlled by external stimuli.^[^ ^5^ ^]^ The integration of sensing, memory, and computing could be thus achieved via the regulation of conductivity and carrier.^[^ ^6^ ^]^

Light has been demonstrated to be a promising external stimulus to control the switching of logic‐in‐memory devices.^[^ ^4^, ^7^ ^]^ Compared with the other stimuli, such as pH, thermal, electric field, magnetic field, gas and vapor, light shows unique priority to control molecules and materials with precisely tunable temporal‐spatial resolution and noncontact manner. Photoresponsive molecules exhibit reversible and controllable switching of conformation, conjugation, light absorbance, and polarity under light irradiation and/or heat.^[^ ^8^ ^]^ Therefore, photoresponsive materials could be switched between multifunctional states, while the optical properties (color and transmittance), mechanical properties (modulus and rheology), chemical properties (hydrophilicity, polarity, and solubility), and electrical properties (conductivity and dielectric properties) could be controlled.^[^ ^9^ ^]^

Donor–acceptor Stenhouse adducts (DASAs) as a series of novel photoresponsive molecules have extensively gained researchers’ sights since 2014.^[^ ^10^ ^]^ With a triene *π*‐bridge located between electron‐donating and ‐withdrawing moieties, DASAs exhibit reversible isomerization between *linear* and *cyclic* under controlling of visible/near‐infrared (NIR) light and heat.^[^ ^11^ ^]^ The molecular geometrical structure and conjugation property of DASAs could be reversibly switched, which is attractive in the photomodulation for logic‐in‐memory devices.^[^ ^11^, ^12^ ^]^ Compared with the traditional photoswitches (e.g., azobenzene, spiropyran, and diarylethene),^[^ ^8b^ ^–^ ^d^ ^]^ which exhibit ultraviolet light‐induced isomerization and are therefore at risk from photobleaching under high energy input, DASAs show the advantages to be triggered by long‐wavelength‐light. Visible and NIR light could penetrate deeply into the materials and are potential to generate an efficient control of the properties and functions.

However, adjusting the organic–inorganic interfaces between photoswitches and 2D materials has always been important for the fabrication of light‐controllable logic‐in‐memory devices. Similar to the well‐studied traditional photoresponsive molecules (e.g., azobenzene, spiropyran),^[^ ^8a,c^ ^]^ the isomerization of DASAs is hindered in the solid state due to the strong intermolecular *π*–*π* aggregation, which limits the photomodulation of logic‐in‐memory devices. Therefore, it is necessary to optimize the organic–inorganic interfaces to promote the isomerization between *linear* and *cyclic* DASAs on the surface of 2D materials. Fast and efficient isomerization requires sufficient molecular mobility and a suitable physicochemical environment.^[^ ^13^ ^]^ Generally speaking, introducing bulky substituted groups with soft structures (e.g., alkyl chains, ethylene glycol chains) weakens the intermolecular *π*–*π* interaction between photoresponsive molecules, which further increases the free volume for isomerization in the solid state.^[^ ^14^ ^]^ Moreover, the alkyl chains provide a solvent‐like physicochemical environment in the solid state, where the photoresponsive molecules are surrounded by the saturated carbon spacers.

**Scheme 1:**
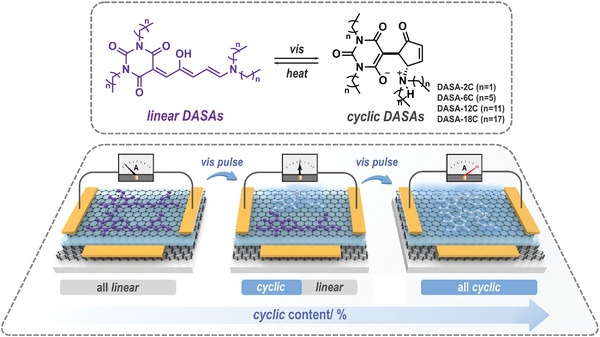
Schematic illustration of modulating the 2D logic‐in‐memory devices by visible light and heat.

However, the relationships between the length of introduced alkyl chains and photoisomerization of DASAs on surface have not been well‐understood. In this work, we synthesized a series of DASAs with four‐armed carbon spacers (termed as **DASA‐2C**, **DASA‐6C**, **DASA‐12C**, and **DASA‐18C**, respectively) (**Scheme** **1**). The isomerization properties (kinetics and efficiency) between *linear* and *cyclic* DASAs in the solid state were adjusted by varying the carbon spacer lengths: 1) Prolonging the carbon spacers weakens the intermolecular *π*–*π* aggregation in the solid state and improves both the forward (*linear*‐to‐*cyclic*) and backward (*cyclic*‐to‐*linear*) isomerization of DASAs. 2) However, too long carbon spacers cause crystallization and hinder the isomerization. The photoisomerization process of DASAs on graphene surface was further understood by density functional theory (DFT) calculation. The 2D logic‐in‐memory devices were fabricated by assembling DASAs on the surface. The drain–source current (*I*_ds_) of the 2D devices was dynamically controlled and reversibly switched based on the *linear*‐*cyclic* isomerization under visible light (*λ* = 520 nm) and heat (Scheme 1). Moreover, **DASA‐12C** was selected to achieve the multistage photomodulation for the 2D logic‐in‐memory devices, which was realized by well‐controlling the irradiation time and intensity.

## 2 Results and Discussion

**Figure 1:**
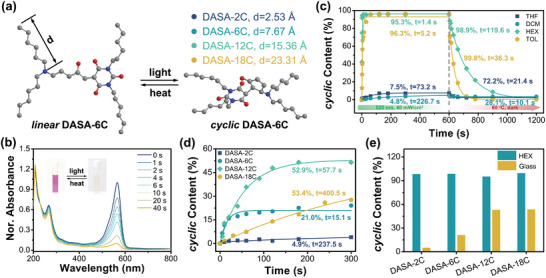
Isomerization performance of DASAs in solutions and in the solid state. a) Calculated molecular structure of *linear* and *cyclic* **DASA‐6C**, *d* represents the length of carbon spacers for **DASA‐2C**, **DASA‐6C**, **DASA‐12C**, and **DASA‐18C**. b) Normalized absorbance of **DASA‐12C** ([**DASA‐12C**] = 0.01 mm in HEX) under green light irradiation (520 nm, 40 mW cm^−2^) for various time intervals, inner shows the photographic images under light and in the dark. c) Time‐dependent *linear*‐to‐*cyclic* (left, 520 nm, 40 mW cm^−2^) and *cyclic*‐to‐*linear* (right, in the dark) isomerization of **DASA‐12C** in various solutions ([**DASA‐12C**] = 0.01 mm). d) Time‐dependent *linear*‐to‐*cyclic* (520 nm, 40 mW cm^−2^) isomerization of DASAs in the solid state (on glass surface). e) Summary of *cyclic* isomer contents at equilibrium for the DASAs in HEX (DASAs] = 0.01 m) and in the solid state (on glass surface).

DASAs with four‐armed carbon spacers were synthesized according to a modified strategy based on the previous reports (**Figure** **1**a and Schemes S1–S4, Supporting Information). Introducing alkyl chains does not obviously contribute to the electron distribution on the triene *π*‐bridge. Therefore, all the DASAs exhibit a strong *n*‐π* absorption band at ≈564 nm in hexane (HEX), generating bright purple solutions, which is resulted from the push–pull nature of DASAs (Figure 1b and Figures S1–S4, Supporting Information). All the synthesized DASAs belong to the first generation of DASAs, which show fast and efficient isomerization in nonpolar solvents (e.g., toluene and HEX) under controlling of green light (40 mW cm^−2^) and heat (60 °C in the dark), while the *linear* isomers are stabilized in part of polar solvents (e.g., dichloromethane (DCM), tetrahydrofuran) (Figure 1c and Figures S5–S7, Supporting Information). Both the *linear*‐to‐*cyclic* and *cyclic*‐to‐*linear* isomerization of DASAs follow the first‐order kinetics in solutions, which is in line with previous reports.^[^ ^12^, ^15^ ^]^ The first‐order kinetics also fits well with the photoisomerization of traditional photoresponsive molecules, such as azobenzene^[^ ^16^ ^]^ and spiropyran.^[^ ^8^, ^17^ ^]^

DASAs were deposited on the glass surface to investigate the isomerization properties in the solid state. **DASA‐2C** shows a splitting and widening *n*‐π* absorption band at ≈540–650 nm, which is attributed to the strong intermolecular *π*–*π* aggregation (Figures S8 and S9, Supporting Information). The *n*‐π* absorption band gradually narrows with prolonging the carbon spacers for **DASA‐6C** and **DASA‐12C**, which however broadens again for **DASA‐18C** (Figures S10–S15, Supporting Information). Due to the hindered molecular mobility in the solid state, the *linear*‐to‐*cyclic* photoisomerization of **DASA‐2C** is limited on surface, where only 4.9% *linear* **DASA‐2C** switches to *cyclic* under green light irradiation (40 mW cm^−2^) for 237.5 s (fitted data) (Figure 1d). The equilibrium of photoisomerization is obviously promoted for **DASA‐6C**, **DASA‐12C**, and **DASA‐18C**, where the isomerization efficiency keeps increasing with prolonging the alkyl chains. Over 50% of *linear* **DASA‐12C** and **DASA‐18C** switch to *cyclic* at equilibrium (Figure 1d). DASAs with various carbon spacer lengths exhibit high and similar *linear*‐to‐*cyclic* photoisomerization efficiency in HEX (>95%) (Figure 1e). On the other hand, the photoisomerization efficiency in the solid state increases to a limitation at ≈55% with prolonging the carbon spacers, which is still far from that in solutions. These indicate that the introduction of the four‐armed carbon spacers structure eliminates the intermolecular aggregation of DASAs, which further provides sufficient molecular mobility and benefits the isomerization in the solid state. The alkyl chains also provide a closed physicochemical environment in the solid state with that in nonpolar solutions.

**Figure 2:**
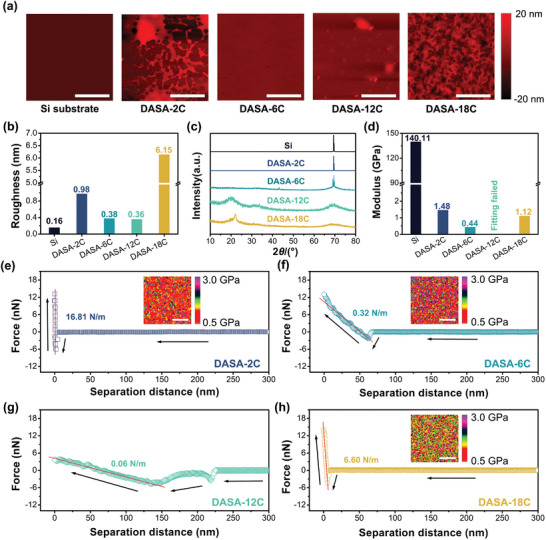
Microstructure formed by DASAs in the solid state. a) AFM images (morphology) of the DASAs films on silicon wafer substrates surface, scare bar: 2 µm. b) Roughness on the surface of the DASAs films (obtained from AFM images). c) XRD patterns of silicon wafer substrates and DASAs films. d) Young's moduli of silicon wafer substrates and DASAs films (obtained from force–distance curves). e) Force–distance curve (approach) of the **DASA‐2C** films on silicon wafer substrates surface, inner shows the modulus map, scale bar: 2 µm. f) Force–distance curve (approach) of the **DASA‐6C** films on silicon wafer substrates surface, inner shows the modulus map, scale bar: 2 µm. g) Force–distance curve (approach) of the **DASA‐12C** films on silicon wafer substrates surface, inner shows the modulus map, scale bar: 2 µm. h) Force–distance curve (approach) of the **DASA‐18C** films on silicon wafer substrates surface, inner shows the modulus map, scale bar: 2 µm.

Interestingly, keeping prolonging the carbon spacers does not trivially promote the *linear*‐to‐*cyclic* photoisomerization of DASAs in the solid state. The transition efficiency of **DASA‐18C** and **DASA‐12C** at equilibrium is similar, but **DASA‐18C** takes a much longer time to reach the equilibrium (Figure 1d). To understand the relationship between the carbon spacer lengths and the isomerization kinetics, the assembled microstructure of DASAs on silicon wafer substrates was monitored using an atom force microscope (AFM). Due to the conjugated planar structure, **DASA‐2C** exhibits poor film‐forming property, which generates plenty of fragments on surface and induces an apparent increase in roughness (**Figure** **2**a,b). By prolonging the carbon spacers, a smooth film of **DASA‐6C** and **DASA‐12C** are formed on the surface. Nevertheless, further increasing the length of carbon spacers leads to the formation of needle‐like crystals (with the length and width of ≈700 and ≈14 nm, respectively) in the film of **DASA‐18C**, which can be attributed to the crystallization of alkyl chains (Figure 2a). The films of **DASA‐6C**, **DASA‐12C**, and **DASA‐18C** show similar thickness between ≈70 and ≈100 nm (Table S1 and Figure S16, Supporting Information). As a consequence, the surface roughness is obviously enhanced for the film of **DASA‐18C** (Figure 2b). The kinetics and transition efficiency at equilibrium of the photoisomerization of DASAs might be further improved by optimizing the assembled microstructure on surface.

The crystallization of DASAs on the solid surface was further investigated by X‐ray diffraction (XRD). Silicon substrates exhibit sharp and clear diffraction signals at 2*θ* = 69.4° and 69.6°, respectively (Figure 2c). The deposition of DASAs on the surface gradually broadens the typical peaks for silicon substrates, indicating the physicochemical environment near the surface is occupied by the alkyl chains. With the extending of carbon spacers (**DASA‐12C**), a broad diffraction peak is observed at 2*θ* = ≈15–25°, which is ascribed to the formed amorphous phase. This soft phase provides sufficient molecular mobility and promotes the isomerization between *linear* and *cyclic* DASAs. However, further prolonging the carbon spacers induces crystallization of the alkyl chains (**DASA‐18C**), and a sharp diffraction peak could be noticed at 2*θ* = ≈22.5° (Figure 2c). These results are with good accordance to the microscopic images.

The mechanical properties of the films are closely interrelated with the isomerization of DASAs in the solid state, which was characterized using fast force volume in AFM‐nDMA mode. A linear regime is always observed in the acquired force–distance curves, in which the loading force monotonically increases with the indenting distance (Figure 2d,h and Figures S17–S20, Supporting Information). This linear response suggests an elastic deformation of the assembled DASAs films under the mechanical load, and its slope defines an effective stiffness quantifying the deformability.^[^ ^18^ ^]^ Obviously, the rigidity of the DASAs films shows a nontrival dependence on the length of the carbon spacers, which first decreases from 16.81 N m^−1^ for **DASA‐2C** to 0.06 N m^−1^ for **DASA‐12C**, and then increases to 6.60 N m^−1^ for **DASA‐18C** (Figure 2e–h). The mechanical properties of diverse DASAs can be further quantified by extrapolating the Young's moduli from the force–distance curves using the standard Johnson–Kendall–Robert model,^[^ ^19^ ^]^ which gives an average value of 1.48, 0.44, and 1.12 GPa for the films of **DASA‐2C**, **DASA‐6C**, and **DASA‐18C**, respectively (Figure 2d). These findings also serve as solid evidences for the proposed isomerization kinetics, where the fast and efficient photoisomerization is achieved in the DASAs films with low Young's moduli (Figure 1d,e).

**Figure 3:**
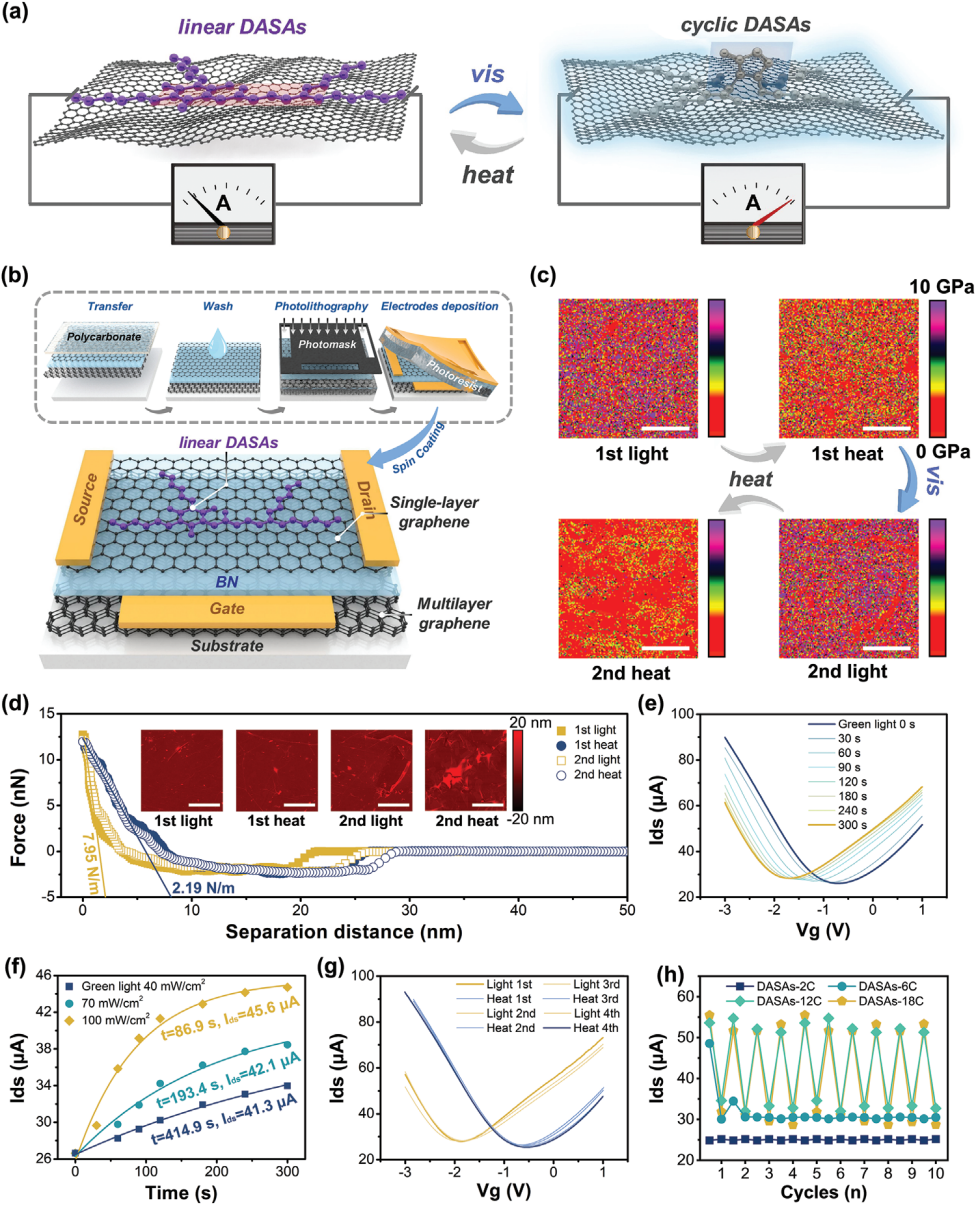
Light‐controlling electrical properties of the 2D logic‐in‐memory devices. a) Schematic illustration of controlling the electrical properties of the devices by triggering the isomerization between *linear* and *cyclic* DASAs. b) Schematic illustration of fabricating the 2D logic‐in‐memory devices. c) Modulus maps of the 2D logic‐in‐memory devices surface after repeated treatment of green light (100 mW cm^−2^, 300 s) and heat (40 °C, 300 s), scale bar: 2 µm. d) Force–distance curves of the 2D logic‐in‐memory devices surface after repeated treatment of green light (100 mW cm^−2^, 300 s) and heat (40 °C, 300 s), inner shows the morphology maps after each treatment, scale bar: 2 µm. e) *I*_ds_–*V*_g_ curves of the devices with **DASA‐18C** after green light irradiation (100 mW cm^−2^). f) Time‐dependent *I*_ds_ variation of the devices with DASA‐18C under green light irradiation with different intensities. g) *I*_ds_–*V*_g_ curves of the devices with **DASA‐18C** after repeated treatment of green light (100 mW cm^−2^, 300 s) and heat (40 °C, 300 s). h) The *I*_ds_ variation of the devices after repeated treatment of green light (100 mW cm^−2^, 300 s) and heat (40 °C, 300 s) for ten cycles.

Due to the reversible isomerization between *linear* and *cyclic* DASAs on surface, the electrical properties of the 2D logic‐in‐memory devices could be switched by green light irradiation and heat (**Figure** **3**a). The 2D logic‐in‐memory devices were fabricated through the following procedure (Figure 3b): 1) Transfer a composite film of monolayer graphene/hexagonal boron nitride (h‐BN)/sublayer graphene to silicon wafer substrate surface (transfer and wash). 2) Design and form the electrodes on the heterostructure (photolithography and electrodes deposition). 3) Deposit DASAs layer on the surface of graphene (spin coating) (Scheme S5 and Figure S21, Supporting Information).

The isomerization between *linear* and *cyclic* DASAs generates reversible switching of the mechanical properties on the 2D logic‐in‐memory devices surface. The light‐induced *linear*‐to‐*cyclic* isomerization of **DASA‐12C** results in a rigid film with an average Young's modulus of 83.6 GPa (Figure 3c,d). By contrast, after heating at 40 °C for 300 s, the thermal‐induced *cyclic*‐to‐*linear* isomerization of **DASA‐12C** would lead to a striking decrease in the rigidity by over 40 times, and the average Young's modulus is reduced to 2.0 GPa. The reversible switching of the mechanical properties can be realized by sequentially treating the 2D logic‐in‐memory devices with green light and heat, which has been further demonstrated by the PeakForce quantitative nanomechanical mapping (Figure 3c). The force–distance curves for the **DASA‐12C** deposited on the devices overlap after the periodical treatment under green light irradiation (100 mW cm^−2^, 300 s) and heat (40 °C, 300 s), while no obvious changes are identified on the surface roughness (Figure 3d). Interestingly, the light‐induced increase of rigidity on the 2D logic‐in‐memory devices hinders the proceeding of *linear*‐to‐*cyclic* isomerization, which implies a limitation effect of the transition efficiency at equilibrium (Figure 1e).

The devices exhibit negatively shifted threshold voltage (*V*_g_) and gradually increased *I*_ds_ upon irradiation using **DASA‐6C**, **DASA‐12C**, and **DASA‐18C** as the photoswitches (Figure 3e and Figures S22–S27, Supporting Information). It should be notable that as the control, the devices without DASAs do not show obvious *I*_ds_–*V*_g_ curve shift even after green light irradiation with high intensity (125 mW cm^−2^ for 300 s), indicating the *V*_g_ shift is resulted from the photoisomerization (Figure S28, Supporting Information). On the other hand, due to the limited light‐induced *linear*‐to‐*cyclic* isomerization of **DASA‐2C** in the solid state, the *I*_ds_–*V*_g_ curve of the corresponding devices does not shift after irradiation (Figure S29, Supporting Information). Interestingly, the photomodulation of *I*_ds_ follows the first‐order kinetics, which is in good accordance with the previous results on the photoisomerization process in the solid state^[^ ^20^ ^]^ (Figure 3f). While using **DASA‐18C** as the photoswitches, the *I*_ds_ gradually increased from 26.2 to 41.3 µA at equilibrium under 40 mW cm^−2^ green light irradiation, while the time needed is 414.9 s (fitted data) (Figure 3f and Figures S30–S33, Supporting Information). Both the modulation range and kinetics are further improved using green light with higher intensity and the *I*_ds_ at equilibrium reaches 45.6 µA under 100 mW cm^−2^ green light irradiation for 86.9 s (fitted data) (Figure 3f).

The reversible switching of the *I*_ds_ of the 2D logic‐in‐memory devices was realized by sequentially treating with green light irradiation and heat. The *I*_ds_–*V*_g_ curves for both the devices with **DASA‐12C** and **DASA‐18C** are fully reversible under green light irradiation (100 mW cm^−2^, 300 s) and heat (40 °C, 300 s) (Figure 3g and Figures S34–S36, Supporting Information). No obvious loss of the *I*_ds_ is observed in ten cycles (Figure 3h). Therefore, the dynamic doping control window (photomodulation range) could be calculated as 19.7 and 22.7 µA for the devices with **DASA‐12C** and **DASA‐18C** (Figures S37 and S38, Supporting Information). However, both the devices with **DASA‐2C** and **DASA‐6C** exhibit poor reversibility on the *I*_ds_, which might be attributed to the strong intermolecular *π*–*π* aggregation between the DASAs or between the DASAs and graphene surface (Figure 3h and Figure S39 and S40, Supporting Information, will be discussed in the following section).

**Figure 4:**
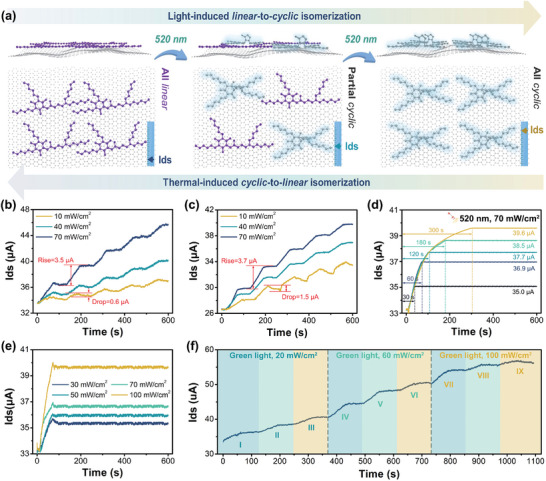
Multistage photomodulation of the 2D logic‐in‐memory devices. a) Schematic illustration of achieving the multistage photomodulation of the 2D logic‐in‐memory devices by well‐controlling the isomerization of DASAs on surface. b) Time‐dependent *I*_ds_ variation of the devices with **DASA‐12C** under green light irradiation with different intensities (periodically treated by light for 60 s and dark for 60 s). c) Time‐dependent *I*_ds_ variation of the devices with **DASA‐18C** under green light irradiation with different intensities (periodically treated by light for 60 s and dark for 60 s). d) Retention curves of *I*_ds_ for the devices with **DASA‐12C** under green light irradiation for different time (light intensity is kept at 70 mW cm^−2^). e) Retention curves of *I*_ds_ for the devices with **DASA‐18C** under green light irradiation with different intensities (irradiation time is kept at 60 s). f) Time‐dependent *I*_ds_ variation of the devices with **DASA‐12C** under green light irradiation, the light intensity, and irradiation time are controlled to achieve multistage photomodulation.

To achieve multistage photomodulation and programming for the 2D logic‐in‐memory devices, the *cyclic* content of DASAs needs to be adjusted on the surface of the devices. Compared with that in solutions, the thermal‐triggered *cyclic*‐to‐*linear* isomerization slows down in the solid state (Figure 1c). Therefore, multiple intermediate states between the initial (OFF state, all *linear*) and photostationary state (ON state, all *cyclic*) are possible to be achieved by controlling the irradiation time and intensity (**Figure** **4**a). Based on the results of the photoisomerization and reversibility of DASAs on the 2D logic‐in‐memory devices surface, **DASA‐12C** and **DASA‐18C** were selected as the photoswitches. The devices were periodically treated with green light irradiation (60 s on and 60 s off), while the irradiation intensity was kept at 10, 40, and 70 mW cm^−2^, respectively. Both the devices with **DASA‐12C** and **DASA‐18C** exhibit sharp *I*_ds_ increase at the beginning of under green light irradiation (Figure 4b,c). The *I*_ds_ increases faster for the devices with **DASA‐18C**, which increases by 3.7 µA under 70 mW cm^−2^ green light irradiation for 60 s (Figure 4c). The *I*_ds_ drop could be noticed for both the devices with **DASA‐12C** and **DASA‐18C** after green light irradiation with low intensity, which is negligible when the irradiation intensity is strong (Figure 4b,c). The devices with **DASA‐18C** exhibit an *I*_ds_ drop of 1.5 µA after 10 mW cm^−2^ green light irradiation, which is more than two times higher than those with **DASA‐12C**. This is because the introduction of long carbon spacers induces crystallization of the alkyl chains on graphene surface, which might stabilize the *linear* isomers and promote the *cyclic*‐to‐*linear* thermal relaxation. Therefore, for the multistage photomodulation of 2D logic‐in‐memory devices, prolonging the alkyl chains of photoswitches is not always the right way to go: 1) On the one hand, the promoted isomerization between *linear* and *cyclic* DASAs extends the photomodulation range and kinetics. 2) On the other hand, the spontaneously occurred thermal‐relaxation does not benefit the multistage photomodulation. Selecting the suitable carbon spacers and balancing the forward (*linear*‐to‐*cyclic*) and backward (*cyclic*‐to‐*linear*) isomerization is necessary.

We track the time‐dependent *I*_ds_ of the devices with **DASA‐12C** after green light irradiation with adjusted time and intensity (Figure 4d,e). The initial *I*_ds_ is 33.4 µA, denoting the OFF state of the transistor. We fabricated nine logic‐in‐memory devices with **DASA‐12C**, which exhibit the initial *I*_ds_ between 31.9 and 35.4 µA, indicating a tiny device‐to‐device variation (Table S2, Supporting Information). The yield of the devices is ≈67%. Upon green light irradiation (70 mW cm^−2^) for 30 s, the partial *linear*‐to‐*cyclic* isomerization of **DASA‐12C** determines a negative shift of *V*_g_ and further induces a rise of *I*_ds_ to 35.0 µA (Figure 4d). Extending the irradiation time to 300 s further increases the *I*_ds_ up to 39.6 µA. Multi‐intermediate memory states could be defined by well‐controlling the irradiation time (60 s for 36.9 µA, 120 s for 37.7 µA, and 180 s for 38.5 µA). Moreover, due to the stabilized *cyclic* isomers, the *I*_ds_ does not obviously drop after removing the light. The increase of *I*_ds_ is getting slower while extending the irradiation time because of the first‐order kinetics.

Light intensity was demonstrated to be another variable to modulate the 2D logic‐in‐memory devices by rebuilding the equilibrium. The light intensity varied between 30 and 100 mW cm^−2^, while the irradiation time was kept the same at 60 s (Figure 4e). The *I*_ds_ increases from 33.4 to 35.3 µA under 30 mW cm^−2^ green light irradiation, which was further promoted to 36.0, 36.8, and 39.6 µA with the light intensity of 50, 70, and 100 mW cm^−2^, respectively.

The 2D logic‐in‐memory devices could be further modulated by well‐controlling the irradiation time and intensity (Figure 4f). The devices with **DASA‐12C** were first treated with green light irradiation with low intensity (20 mW cm^−2^), and the *I*_ds_ increases from 33.4 to 36.1, 38.3, and 40.7 µA step‐by‐step (Figure 4f, steps I, II, and III). After the *linear*‐to‐*cyclic* isomerization of **DASA‐12C** reaches equilibrium, the green light irradiation intensity was increased to 60 mW cm^−2^, and a new kinetics was built (Figure 4f, step IV, V and VI). Further increasing the light intensity to 100 mW cm^−2^ extends the photomodulation range and the *I*_ds_ as high as 56.8 µA is reached (Figure 4f, step VII, VIII, and IX).

**Figure 5:**
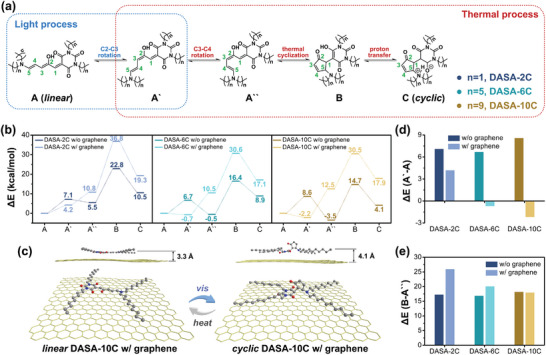
Mechanism of DASAs isomerization on graphene surface. a) Schematic illustration of the main isomerization route between *linear* and *cyclic* DASAs. b) Calculated molecular energy variation through the *linear‐*to‐*cyclic* isomerization of **DASA‐2C** (left), **DASA‐6C** (middle), and **DASA‐10C** (right) with and without graphene. c) Schematic illustration of the calculated molecular structure of *linear* and *cyclic* **DASA‐10C** on the surface of graphene. d) Relative molecular energy variation of DASAs through the A‐A` transition step. e) Relative molecular energy variation of DASAs through the A``‐B transition step.

The isomerization between *linear* and *cyclic* DASAs with various carbon spacers length on graphene surface were further revealed by DFT calculations. **DASA‐2C**, **DASA‐6C**, and **DASA‐10C** (*n* = 9, replaced for **DASA‐12C**) were modeled, and five key structures from 14 intermediates in the isomerization pathway of each modeled molecules respectively were investigated (**Figure** **5**a).^[^ ^21^ ^]^ DASAs in *linear* form (A) absorb photons in the visible light region and result in sequential *trans*‐to‐*cis* transition of C2—C3 and C3—C4 bonds on the triene *π*‐bridge, inducing the A‐A`‐A`` isomerization (Figure 5a). The formation of A`` increases the relative activity of proton on the —OH on triene *π*‐bridge, which results in the intramolecular proton transfer from —OH to C=O on the electron‐withdrawing moiety, triggering the cyclization and A``‐B transition. The proton further transfers to the nitrogen on the electron‐donating moiety through tautomerization and generates *cyclic* DASAs (C).

The calculated results showed that all the DASAs had similar variation tendency of the relative molecular energy variation (Δ*E*) in the gas phase (Figure 5b and Figures S41–S46, Supporting Information) the calculated results were included in the Supporting Information). All the DASAs show similar variation tendencies of the Δ*E* in the gas phase, where the A‐A` and A``‐B transition are the main steps exhibiting Δ*E* increase during the *linear*‐to‐*cyclic* isomerization process. Therefore, these two steps hinder the proceeding of *linear*‐to‐*cyclic* isomerization of DASAs (Figure 5b). Interestingly, introducing graphene decreases the Δ*E* needed to go across for the A‐A` transition of all the DASAs (Figure 5c,d). Moreover, with the prolonging of carbon spacers, the Δ*E* of A‐A` transition keeps decreasing from 4.2 (**DASA‐2C**) to −2.2 kcal mol^−1^ (**DASA‐10C**) on the surface of graphene (Figure 5d). On the other hand, the Δ*E* of A‐A` transition does not obviously change for **DASA‐2C**, **DASA‐6C**, and **DASA‐10C** in the gas phase.

The cyclization (A``‐B transition) is the key step in the *linear*‐to‐*cyclic* isomerization pathway and the intramolecular proton transfer has been demonstrated to be important.^[^ ^22^ ^]^ **DASA‐2C** shows a sharp increase of Δ*E* of A``‐B transition after immobilizing on the graphene surface, which limits the *linear*‐to‐*cyclic* photoisomerization (Figure 5b,e). This value is further decreased by prolonging the carbon spacers of DASAs. These might be attributed to the interaction between *linear* DASAs and graphene. The distance between *linear* DASAs and graphene surface was calculated to be ≈3.3 Å, indicating the existence of strong *π*–*π* interaction (Figure 5c). The long carbon spacers might weaken the *π*–*π* interaction between *linear* DASAs and between *linear* DASAs and graphene surface, which further promotes the *linear*‐to‐*cyclic* isomerization. Moreover, the electron transition through the interfaces between DASAs and graphene is supposed to be the mechanism of photomodulation of the 2D logic‐in‐memory devices (Figures S47 and S48, Supporting Information).

## 3 Conclusion

In summary, we fabricated 2D logic‐in‐memory devices by depositing DASAs with various carbon spacers on the surface of graphene and achieved multistage photomodulation by controlling the visible light irradiation time and intensity. The isomerization properties (kinetics and equilibrium) between *linear* and *cyclic* DASAs in the solid state were demonstrated to be closely interrelated with the length of carbon spacers: 1) Prolonging the carbon spacers generates amorphous phase with low rigidity, which weakens the intermolecular *π*–*π* aggregation and promotes both the *linear*‐to‐*cyclic* photoisomerization and *cyclic*‐to‐*linear* thermal‐relaxation of DASAs. 2) Too long carbon spacers induce crystallization of the alkyl chains, which increases the surface rigidity and hinders the isomerization instead. Green light irradiation induces negatively shifted *V*_g_ and gradually increased *I*_ds_ of the 2D logic‐in‐memory devices, which is reversible by heating in the dark. Optimization of carbon spacers length is necessary for the multistage photomodulation of the devices, while the irradiation time and intensity were carefully adjusted to reach multi‐intermediates between the OFF (initial) and ON (equilibrium) states. Moreover, the photoisomerization mechanism of DASAs on graphene surface was understood with the assistance of DFT calculation. Prolonging of carbon spacers was demonstrated to lower the relative molecular energy variation of key steps in the photoisomerization process of DASAs on graphene surface, which thermodynamically promotes the *linear*‐to‐*cyclic* isomerization. This work supports the important and versatile applications of photoswitches to accomplish advanced electronic functions, and offers a nonconventional strategy to control nanodevices in a precise, efficient, and noncontact manner. We envision the performance of 2D logic‐in‐memory devices could be further improved in the further work by optimizing the distribution of DASAs on surface. Moreover, logic operations including AND, OR, NAND, and NOR might be developed by assembling of devices.

## 4 Experimental Section

### Synthesis of DASAs

Four DASAs involved here (**DASA‐2C**, **DASA‐6C**, **DASA‐12C**, and **DASA‐18C**) were typical first‐generation of DASAs. The synthesis of DASAs with various alky chain lengths was summarized below: 1) Barbituric acid as the electron‐withdrawing moiety was synthesized by ethyl‐, hexyl‐, dodecyl‐, and octadecyl‐substituted isocyanate and amine via an exothermic reaction under assistance of malonyl chloride. 2) The precursor of DASAs was synthesized through the knoevenagel condensation between barbituric acid and furfural. 3) DASAs were synthesized by the (aza‐)Piancatelli rearrangement reaction with the purchased electron‐donating moiety.

**DASA‐2C** was taken as an example. Under nitrogen, 20 mmol ethane isocyanate (hexyl‐, dodecyl‐, and octadecyl‐substituted for **DASA‐6C**, **DASA‐12C**, and **DASA‐18C**, respectively) was dissolved in 30 mL DCM, which was added to a solution of 20 mmol ethylamine (hexyl‐, dodecyl‐, and octadecyl‐substituted for **DASA‐6C**, **DASA‐12C**, and **DASA‐18C**, respectively) in 100 mL DCM. The mixture was stirred at room temperature for 2 h. Subsequently, malonyl chloride (20 mmol) was added under ice bath. The mixture was refluxed for 1 h and the solution was cooled to room temperature and then quenched with 1 n HCl (20 mL*3). The crude product was extracted with DCM (60 mL*3) and the organic phase was collected and dried with Na_2_SO_4_. Solvents were removed under rotary evaporation and further purified by column chromatography to give the barbituric acid with various alkyl chain lengths.

The as‐prepared barbituric acid compounds (5 mmol) and 2‐furaldehyde (5 mmol) were added into 20 mL of DCM and stirred at room temperature for 1 h until the mixture became deep yellow. Subsequently, solvents were removed by rotary evaporation and further purified by column chromatography to give the precursor of DASAs.

The precursor of DASAs (5 mmol) and 5 mmol diethylamine (dihexylamine, didodecylamine, and dioctadecylamine for **DASA‐6C**, **DASA‐12C**, and **DASA‐18C**, respectively) were added into 20 mL of DCM and the color of the solution turned to purple immediately. The mixture was stirred for 2 h at 45 °C. Solvents were removed by rotary evaporation and further purified by column chromatography to give the DASAs with various alkyl chain lengths.

### Fabrication of DASAs Films

The DASAs films were fabricated by spin‐coating the solutions ([DASAs] = 0.01 m in DCM) at 1000 rpm for 30 s, which was further annealed at 40 °C for 30 min.

### Preparation of the 2D Logic‐in‐Memory Devices

The 2D logic‐in‐memory devices were fabricated via the following procedure:

1. Monolayer graphene/h‐BN/sublayer graphene were picked up by polymer films composed of polycarbonate (PC)/polydimethylsiloxane (PDMS) and then deposited on silicon substrates.
2. The polymer films were dissolved and washed with trichloromethane (CHCl_3_).
3. Electrodes were deposited on the heterostructure surface using the photomask lithography and metal thermal evaporation/lift‐off process.
4. DASAs were spin‐coated on the surface of graphene.

In detail, h‐BN and graphene were exfoliated on the surface of silicon substrates (thickness: 285 nm) using adhesive tapes. The transfer film was composed of a PC supported by PDMS. The viscosity of PC was used to pick up monolayer graphene at 70°C for 3–10 min, and the BN/sublayer graphene was picked up with the same method at 180 °C, which was laid under the monolayer graphene to obtain the heterostructure onto a SiO_2_. After cooled to room temperature, the outmost polycarbonate layer was dissolved and washed by chloroform. The Cr/Au (5 nm/40 nm) electrodes were deposited on the heterostructure surface using the photomask lithography and metal thermal evaporation (evaporation rate: Cr 3 Å s^−1^ and Au 1 Å s^−1^; the pressure of vacuum was 9×10^−5^) and the device was obtained after peeling off the photoresist layer. Afterward, DASAs films were formed on the surface of devices by spin‐coating the solutions ([DASAs] = 0.03 mm in chloroform) at 1000 rpm for 30 s.

## Conflict of Interest

The authors declare no conflict of interest.

## Supporting information

Supporting Information

## Data Availability

The data that support the findings of this study are available from the corresponding author upon reasonable request.
