# Supplementary material for: Controlling Isomerization of Photoswitches to Modulate 2D Logic‐in‐Memory Devices by Organic–Inorganic Interfacial Strategy
Source: Adv Sci (Weinh). 2023 Mar 11;10(13):2207443. doi: 10.1002/advs.202207443 (PMC10161064; doi:10.1002/advs.202207443)
Supplement: Supplementary file 1 — Supporting Information [file ADVS-10-2207443-s001.pdf]

Supporting Information

Controlling isomerization of photoswitches to modulate 2D logic-in-memory devices by organic-inorganic interfacial strategy

Yongli Duan[1]#, Miaomiao Song[1]#, Fanxi Sun[1], Yi Xu[2], Fanfan Shi[3], Hong Wang[3], Yonghao Zheng[1, 4]*, Chao He[5], Xilin Liu[4], Chen Wei[1], Xu Deng[6], Longquan Chen[2]*, Fucai Liu[1]*, Dongsheng Wang[1]*

1. School of Optoelectronic Science and Engineering, University of Electronic Science and Technology of China, Chengdu 610054, China.
2. School of Physics, University of Electronic Science and Technology of China, Chengdu 610054, China.
3. Department of Physics, University of Science and Technology of China, Hefei 230026, China.
4. Department of Orthopedic, Sichuan Provincial People's Hospital & Sichuan Academy of Medical Science & Affiliated Hospital of University of Electronic Science and Technology of China, Chengdu 610072, China.
5. College of Polymer Science and Engineering, Sichuan University, Chengdu 610065, China.
6. Institute of Fundamental and Frontier Science, University of Electronic Science and Technology of China, Chengdu 610054, China.

Content

1. Materials and reagents 3
2. General methods 4
3. Synthesis 6
3.1. Synthesis of DASA-2C 6
3.2. Synthesis of DASA-6C 7

3.3. Synthesis of DASA-12C 9
3.4. Synthesis of DASA-18C 11
4. Photoisomerization of DASAs 13
4.1. Photoisomerization of DASAs in solutions 13
4.2. Photoisomerization of DASAs in the solid state 17
5. Mechanical properties of DASAs films 22
6. Fabrication of 2D logic-in-memory devices 26
7. Light-controlling the 2D logic-in-memory devices 28
8. DFT calculation 39
9. NMR spectra of new chemicals 47
10. Reference 55

1. Materials and reagents

All the chemicals and reagents were directly used without further purification. Furfural ($C_5H_4O_2$, CAS No. 98-01-1), ethane isocyanato (C_3H_5NO, CAS No. 109-90-0), ethylamine (C_2H_7N, CAS No. 75-04-7), diethylamine ($C_4H_{11}N$, CAS No. 109-89-7), malonyl dichloride ($C_3H_2Cl_2O_2$, CAS No. 1663-67-8) and hexyl isocyanate ($C_7H_{13}NO$, CAS No. 2525-62-4) were purchased Aladdin Chemicals. 1-Hexanamine ($C_6H_{15}N$, CAS No. 111-26-2), 1-dodecanamine ($C_{12}H_{27}N$, CAS No. 124-22-1) and dihexylamine ($C_{12}H_{27}N$, CAS No. 143-16-8) were purchased Beijing Merida Technology Co., LTD. Dodecane ($C_{13}H_{25}N$, CAS No. 4202-38-4), didodecylamine ($C_{24}H_{51}N$, CAS No. 3007-31-6), octadecanamine ($C_{18}H_{39}N$, CAS No. 124-30-1), octadecane ($C_{19}H_{37}NO$, CAS No. 112-96-9), dioctadecylamine ($C_{36}H_{75}N$, CAS No. 112-99-2) were purchased from Beijing Innochem Science & Technology co., LTD. Boron nitride (BN, CAS No. 10043-11-5) and graphene (CAS No. 1034343-98-0) were purchased from Guizhou Platinum Strontium Titanium Chemical Products Co. LTD. Polycarbonate (PC, CAS No. 25037-45-0), column-layer chromatographic silica gel and polydimethylsiloxane (PDMS, CAS No. 106214-84-0) was purchased from Chengdu Selwensi Biotechnology Co., LTD. Sodium sulfate (Na_2SO_4, CAS No. 7757-

82-6), hydrochloric acid (HCl, CAS No. 7647-01-0), dichloromethane (CH_2Cl_2, CAS No. 75-09-2), trichloromethane ($CHCl_3$, CAS No. 67-66-3), hexyl hydride (C_6H_{14}, CAS No. 110-54-3), tetrahydrofuran (C_4H_8O, CAS No. 109-99-9) and toluene (C_7H_8, CAS No. 108-88-3) were purchased from Chengdu Keshi-Reagent. All the solvents were used without further purification. Milli-Q water (resistivity: 18.2 MΩ×cm) was used throughout the project. SLYGARD 184 Silicone Elastomer Kit was purchased from The Dow Chemical Company.

2. General methods

Density functional theory (DFT) simulations were performed using CP2K (http://www.cp2k.org) [1] based on the mixed Gaussian and plane-wave scheme [2] and the Quickstep module. [3] The calculation used Perdew-Burke-Ernzerhof (PBE) exchange correlation functional [4], and molecularly optimized short range Double-Zeta-Valence plus Polarization basis set [5] with Goedecker-Teter-Hutter pseudo-potentials [6] (DZVP-MOLOPT-SR-GTH). The plane-wave energy cutoff was 400 Ry, and a Grimme's dispersion correction with Becke-Johnson damping (D3BJ) dispersion correction [7] was applied. The calculation was performed on Gamma point only without symmetry constraint. Structural optimization was performed using the Broyden-Fletcher-Goldfarb-Shannon (BFGS) optimizer until the maximum force fell below 0.00045 Ry/Bohr (0.011 eV/Å). The finite displacement method was used for the phonon calculation, with incremental displacement of 0.01 Bohr (0.0053 Å). HOMO and LUMO diagrams were drawn via *VMD (1.9.3)* software, where the isovalue of HOMO was 0.02 a.u., and that of LUMO was 0.015 a.u.

^{1}H and ^{13}C nuclear magnetic resonance (NMR) spectra were recorded on a Bruker Avance 400 MHz spectrometer.

UV/vis transmittance and absorption spectra were measured on a Shimazu UV-2600. To find out how much excitation light was absorbed by the films of DASAs, the transmittance spectra were transformed into absorbance spectra by equation (1)

$$A = log\frac{1}{T} = log\frac{I_0}{I_t} \quad (1)$$

where A, T, I_0 and I_t represent the absorbance, transmittance, intensity of incident light and intensity of transmission light, respectively. The films of **DASA-6C**, **DASA-12C** and **DASA-18C** show similar absorbance in the visible light region, which is with good accordance to the results of thickness.

X-ray diffraction (XRD) data were collected in the angular range of 2θ =2°~90° with a Bruker D8 Advance X-ray diffractometer.

The elemental mapping was performed on a field-emission scanning electron microscope (SEM) (Carl Zeiss GeminiSEM 300) equipped with an energy-dispersive X-ray spectrometer (EDS). Samples were scattered on silicon wafers and metal sprayed.

The morphology on the films surface was determined with a transmission electron microscopy (TEM) (FEI Tecnai F20) microscope. Samples were prepared on a carbon-coated copper grid.

The morphology on the films surface was determined with an atomic force microscope (AFM) (Bruker Multimode 8) microscope. Samples were spin-coated on the silicon wafer and graphene device.

Drain-source current-threshold voltage (I_{ds}-V_g) curves for the 2D logic-in-memory devices were tested by Pro Plus FS-Pro.

The LED light source with the emitted wavelength at 520 nm was purchased from Zhongjiao Jinyan Systems. The output intensity of the LED was controlled by an LED controller (Zhongjiao Jinyuan Systems) and calibrated by a Laserpoint calibrator (A-02-D12-BBF).

3. Synthesis

All DASAs and its intermediates were synthesized according to a modified strategy based on the previous reports [8].

3.1. Synthesis of DASA-2C

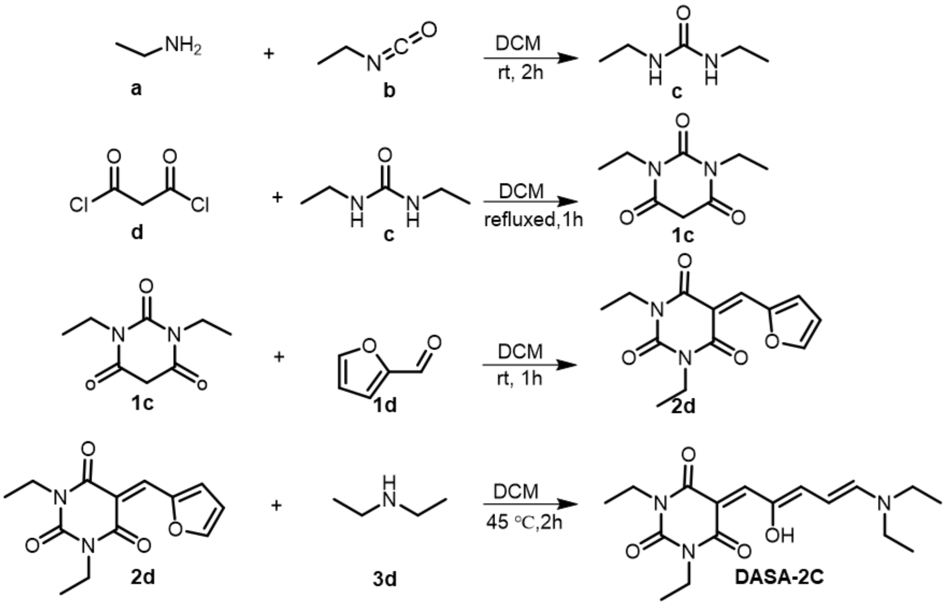

Scheme S1. Schematic illustration of the synthesis of **DASA-2C**.

Synthesis of ***c****:* Under nitrogen atmosphere, ethane isocyanate (compound **b**, 1.44 g, 20 mmol) was dissolved in 30 mL DCM, which was added to a solution of ethylamine (compound **a**, 1 mL, 20 mmol) dissolved in 100 mL DCM. The mixture was stirred at room temperature for 2 h. Subsequently, under ice bath conditions malonyl chloride (compound **d**, 1.94 mL, 20 mmol) was added, and the mixture was refluxed for 1 h, then cooled to room temperature, quenched with 1N HCl (20 mL*3). The crude product was extracted with DCM (60 mL*3), the organic phase was collected and dried with Na_2SO_4. Solvents were removed under rotary evaporation and further purified by column chromatography; 2.42 g yellow oil was collected (yield: 65%).

*Synthesis of **1c**:* 1,3-diethylpyrimidine-2,4,6(1*H*,3*H*,5*H*)-trione (compound **1c**, 1.52 g, 10 mmol) and 2-furaldehyde (compound **1d**, 0.83 mL, 10 mmol) were added sequentially to 35 mL of DCM, the mixture was stirred at room temperature for 1 h until it became a yellow solution. Subsequently, the reaction solution was removed by rotary evaporation and further purified by column chromatography to 2.23 g compound **2c'** as yellow powder was collected (yield: 85%). 1H NMR (400 MHz, $CDCl_3$) δ 1.15 (td, *J*=4.0, 7.1 Hz, 6H), 3.92 (q, *J*=7.0 Hz, 4H), 6.63 (dd, *J*=0.8, 1.7, 3.9 Hz, 1H), 7.75 (d, *J*=1.6 Hz, 1H), 8.32 (s, 1H), 8.53 (d, *J*=3.8 Hz, 1H). ^{13}C NMR (101 MHz, $CDCl_3$) δ 13.30, 13.33, 36.83, 37.55, 111.90, 115.02, 127.76, 140.74, 150.19, 150.48, 151.21, 160.47, 162.01.

*Synthesis of **DASA-2C**:* 1,3-diethylpyrimidine-2,4,6(1*H*,3*H*,5*H*)-trione (compound **2d**, 1.31 g 5 mmol) and diethylamine (compound **3d**, 0.42 mL, 5 mmol) were added sequentially to 30 mL of DCM. The purple mixture was heated to 45 °C and stirred for 2 h. The reaction solution was removed by rotary evaporation and purified by column chromatography 1.26 g **DASA-2C** purple powder was collected (yield: 75%). ^{1}H NMR (400 MHz, $CDCl_3$) δ 1.51 (d, *J*=6.4 Hz, 6H), 1.62 (dd, *J*=7.0, 13.9 Hz, 6H), 3.77 (p, *J*=7.5 Hz, 5H), 4.26 - 4.32 (m, 4H), 6.36 (t, *J*=12.3 Hz, 1H), 7.06 (dd, *J*=1.5, 12.4 Hz, 1H), 7.53 (d, *J*=11.5 Hz, 2H), 12.91 (d, *J*=1.4 Hz, 1H). ^{13}C NMR (101 MHz, $CDCl_3$) δ 164.79, 163.04, 151.53, 146.53, 135.79, 128.27, 125.68, 125.40, 98.89, 34.24, 30.61, 30.06, 21.36, 21.05, 13.73, 13.26, 12.60.

3.2. Synthesis of DASA-6C

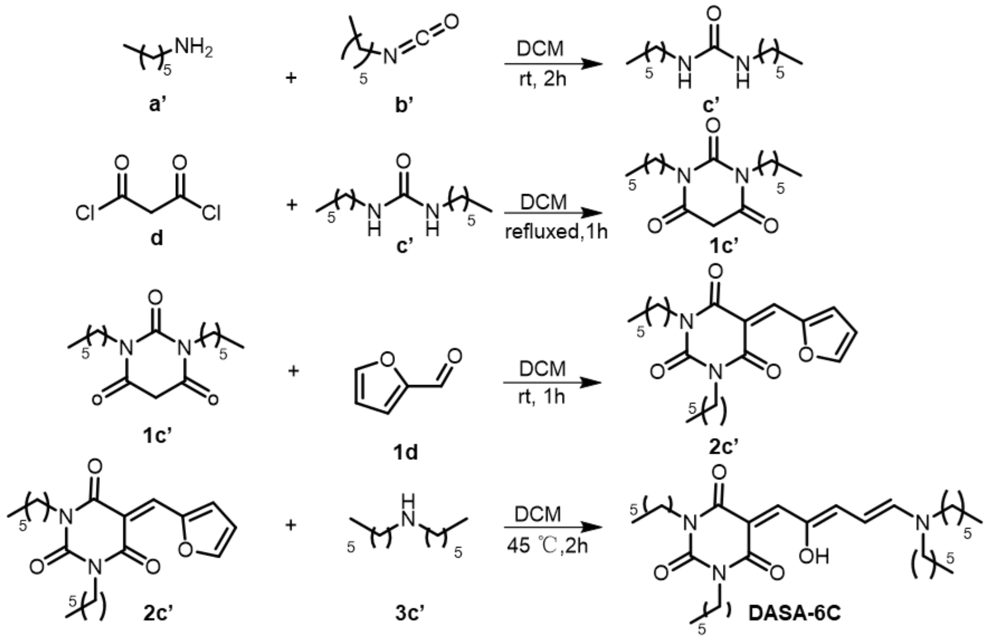

Scheme S2. Schematic illustration of the synthesis of **DASA-6C**.

Synthesis of compound 1c’: Under nitrogen atmosphere, hexyl isocyanate (compound **b’**, 1.28 g 10 mmol) was dissolved in 20 mL DCM as solution A, ethylamine (compound **a’**, 1.01 g, 10 mmol) was dissolved in 80 mL DCM as solution B. Injecting solution A into solution B and the mixture was stirred at room temperature for 2 h. Subsequently, malonyl chloride (compound d, 0.97 mL, 10 mmol) was added under an ice bath, the mixture was refluxed for 1 h and cool to room temperature, quenched with 1N HCl (20 mL*3). The crude product was extracted with DCM (40 mL*3), the organic phase was collected and dried with Na_2SO_4. Solvents were removed by rotary evaporation and further purified by column chromatography to give 2.59 g compound **1c’**as the deep yellow oil (yield: 70%).

Synthesis of compound 2c’: 1,3-dihexylpyrimidine-2,4,6(1*H*, 3*H*, 5*H*)-trione (compound **1c’**, 1.48 g, 5 mmol) and 2-furaldehyde (compound **1d**, 0.42 mL, 5 mmol) were added sequentially to 20 mL of DCM, stirred at room temperature for 1 h until the mixture became a deep yellow solution. Subsequently, solvents were removed by rotary evaporation and further purified by column chromatography to give 1.41 g compound **2c’** as a yellow powder (yield: 75%). ^{1}H NMR (400 MHz, $CDCl_3$) δ 0.78 (m, 6H), 1.26 (h, *J*=5.6, 8.3 Hz, 12H), 1.56 (td, *J*=4.8, 7.5 Hz, 4H), 3.88 (td, *J*=2.0, 7.6 Hz, 4H), 6.63 (m, 1H), 7.76 (d, *J*=1.6 Hz, 1H), 8.34 (s, 1H), 8.55 (d, *J*=3.8 Hz, 1H). ^{13}C NMR (101

MHz, $CDCl_3$) δ 13.98, 14.07, 22.56, 26.58, 28.00, 30.94, 31.47, 31.99, 41.76, 42.45, 43.13, 111.87, 114.67, 127.89, 140.66, 150.85, 151.22, 160.59, 162.19.

*Synthesis of **DASA-6C**:* 1,3-dihexylpyrimidine-2,4,6(1*H*,3*H*,5*H*)-trione (compound **2c'**, 1.41 g 5 mmol) and dihexylamine (compound **3c'**, 0.88 g, 5 mmol) were added sequentially to 30 mL of DCM and the color of solution turned purple immediately. The mixture was heated to 45 °C and stirred for 2 h. After the solution cooled to room temperature, solvents were removed by rotary evaporation and further purified by column chromatography to give 1.71 g **DASA-6C** as the purple oil (yield: 63%). ^{1}H NMR (400 MHz, $CDCl_3$) δ 1.03 (d, *J*=5.5 Hz, 12H), 1.46 (s, 24H), 1.75 (m, 8H), 3.53 (t, *J*=7.5 Hz, 4H), 4.06 (dt, *J*=5.0, 9.5 Hz, 4H), 6.20 (t, *J*=12.3 Hz, 1H), 6.90 (d, *J*=12.4 Hz, 1H), 7.26 (s, 1H), 7.39 (d, *J*=12.3 Hz, 1H), 12.79 (s, 1H). ^{13}C NMR (101 MHz, $CDCl_3$) δ 163.72, 162.00, 156.14, 149.36, 145.32, 138.07, 137.63, 101.54, 97.44, 56.90, 56.51, 48.50, 41.30, 40.28, 39.81, 31.42, 29.58, 28.13, 27.88, 27.60, 26.50, 25.96, 24.98, 24.91, 21.52, 21.35, 20.97, 13.28, 12.85, 12.53.

3.3. Synthesis of DASA-12C

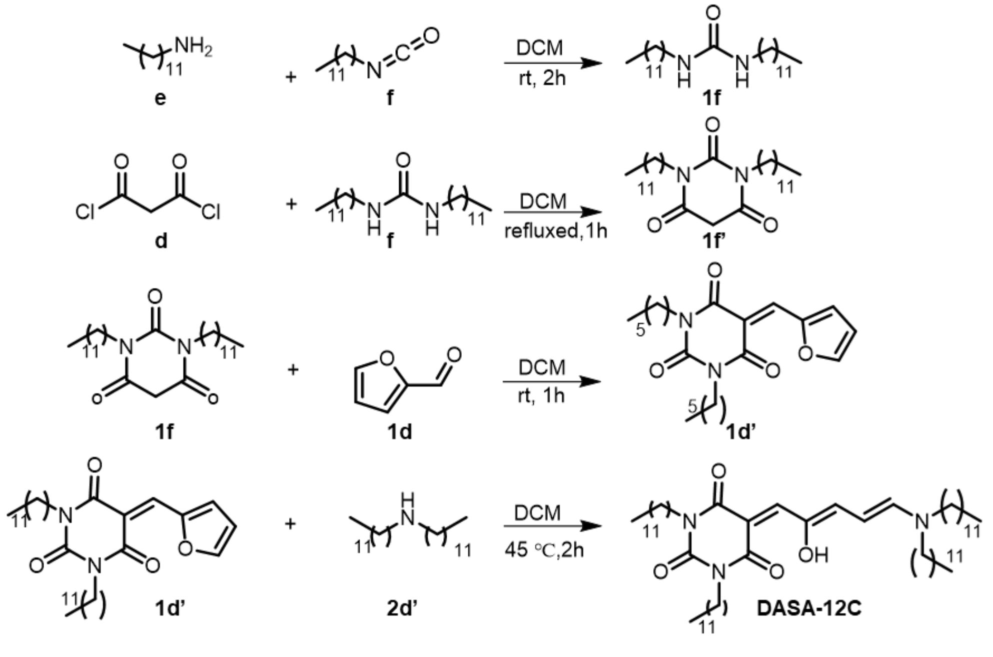

Scheme S3. Schematic illustration of the synthesis of **DASA-12C**.

*Synthesis of compound **1f'**:* Under nitrogen atmosphere, dodecyl isocyanate

(compound **f**, 2.11 g, 10 mmol) was dissolved in 20 mL DCM as solution A, ethylamine dodecylamine (compound **e**, 1.85 g, 10 mmol) was dissolved in 80 mL DCM as solution B. Injecting solution A into solution B and the mixture was stirred at room temperature for 2 h. Subsequently, malonyl chloride (compound **d**, 0.97 mL, 10 mmol) was added under an ice bath, the mixture was refluxed for 1 h. The solution was quenched with 1N HCl (20 mL*3) after the solution cool to room temperature. The crude product was extracted with DCM (40 mL*3), the organic phase was collected and dried with Na_2SO_4. Solvents were removed by rotary evaporation and further purified by column chromatography to give 3.29 g compound **1d** as the yellow oil (yield: 71%).

Synthesis of compound 1d': 1,3-didodecylpyrimidine-2,4,6(1*H*,3*H*,5*H*)-trione (compound **1f**, 2.32 g, 5 mmol) and 2-furaldehyde (compound **1d**, 0.42 mL, 5 mmol) were added sequentially to 20 mL of DCM, stirred at 40°C for 1 h until the color of the mixture became deep yellow. Subsequently, solvents were removed by rotary evaporation and further purified by column chromatography to give 1.76 g compound **1d'** as a yellow powder (yield: 65%).^{1}H NMR (400 MHz, $CDCl_3$) δ 0.80 (t, *J*=6.7 Hz, 6H), 1.15 (m, 36H), 1.52 (m, 4H), 3.88 (td, *J*=2.1, 7.6 Hz, 4H), 6.62 (m, 1H), 7.76 (d, *J*=1.7 Hz, 1H), 8.34 (s, 1H), 8.55 (d, *J*=3.8 Hz, 1H). ^{13}C NMR (101 MHz, $CDCl_3$) δ 14.08, 14.18, 22.70, 23.19, 26.95, 27.49, 28.09, 28.80, 29.35, 29.58, 29.65, 29.87, 30.11, 31.93, 32.42, 41.78, 42.48, 111.89, 127.65, 127.88, 140.67, 140.90, 150.86, 151.25, 160.61, 162.19.

Synthesis of ***DASA-12C:*** 1,3-didodecyl-5-(furan-2-ylmethylene) pyrimidine-2,4,6(1*H*,3*H*,5*H*)-trione (compound **1d'**, 2.71 g 5 mmol) and didodecylamine (compound **2d'**, 1.76 g, 5 mmol) were added sequentially to 30 mL of DCM and the color of the mixture turned purple immediately. The mixture was heated to 45 °C and stirred for 2 h. Solvents were removed by rotary evaporation and further purified by column chromatography to give 2.56 g **DASA-12C** as the purple oil (yield: 56%).^{1}H NMR (400 MHz, $CDCl_3$) δ 0.81 (s, 12H), 1.18 (s, 72H), 3.30 (t, *J*=7.6 Hz, 8H), 3.83 (dd, *J*=5.1, 9.7 Hz, 8H), 5.97 (t, *J*=12.3 Hz, 1H), 6.65 (d, *J*=12.3 Hz, 1H), 7.05 - 7.14 (m, 2H), 12.57 (s, 1H).^{13}C NMR (101 MHz, $CDCl_3$) δ 164.89, 163.14, 156.67, 151.42, 150.29, 146.55, 139.51, 102.44, 98.99, 57.58, 49.57, 41.83, 31.94, 31.91, 29.60, 29.52,

29.37, 29.22, 29.07, 28.24, 27.00, 26.47, 22.70.

3.4. Synthesis of DASA-18C

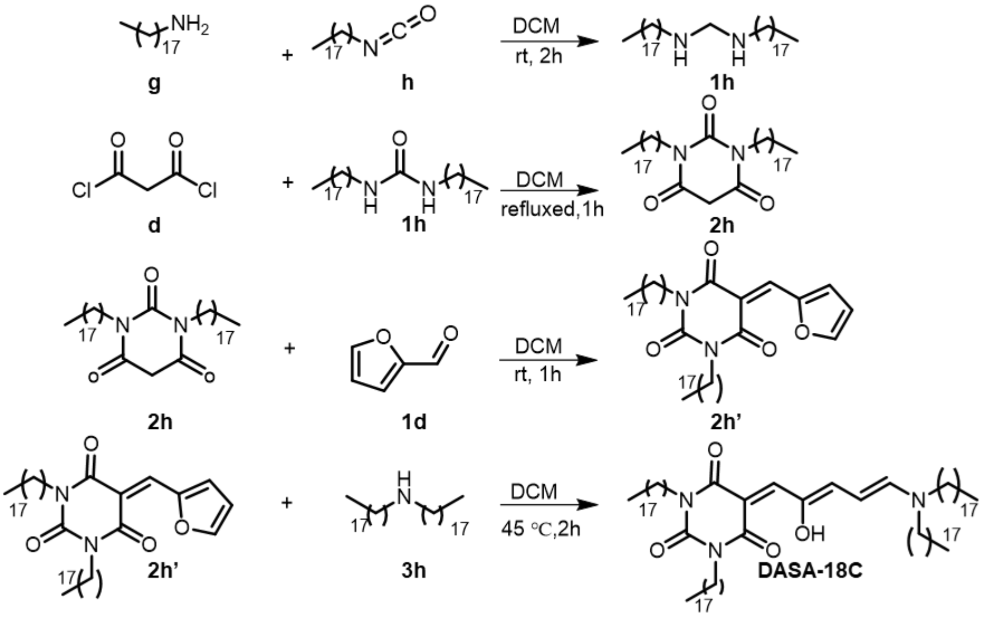

Scheme S4. Schematic illustration of the synthesis of **DASA-18C**.

*Synthesis of compound **2h**:* Under nitrogen atmosphere, octadecyl isocyanate (compound **h**, 2.95 g, 10 mmol) was dissolved in 20 mL DCM as solution A, octadecylamine (compound **g**, 2.69 g, 10 mmol) was dissolved in 80 mL DCM as solution B. Injecting solution A into solution B and the mixture was stirred at room temperature for 2 h. Subsequently, malonyl chloride (compound **d**, 0.97 mL, 10 mmol) was added under an ice bath, the mixture was refluxed for 1 h and cool to room temperature, quenched with 1N HCl (20 mL*3). The crude product was extracted with DCM (40 mL*3), the organic phase was collected and dried with Na_2SO_4. Solvents were removed by rotary evaporation and further purified by column chromatography to give 3.91 g compound **2h** as a deep yellow powder (yield: 63%).

*Synthesis of compound **2h'**:* 1-(octadecan-2-yl)-3-octadecylpyrimidine-2,4,6 (1*H*,3*H*,5*H*)-trione (compound **2h**, 3.16 g, 5 mmol) and 2-furaldehyde (compound **1d**, 0.42 mL, 5 mmol) were added sequentially to 40 mL of DCM, stirred at 40°C for 1 h

till the color of the mixture became deep yellow. Subsequently, Solvents were removed by rotary evaporation and further purified by column chromatography to give 2.09 g compound **2h'** as a light yellow powder (yield:56%).^{1}H NMR (400 MHz, $CDCl_3$) δ 0.81 (t, *J*=6.7 Hz, 6H), 1.18 (m, 60H), 1.53 - 1.60 (m, 4H), 3.83 - 3.93 (m, 4H), 6.65 (dd, *J*=1.6, 3.9 Hz, 1H), 7.76 (d, *J*=1.6 Hz, 1H), 8.35 (s, 1H), 8.55 (d, *J*=3.8 Hz, 1H). ^{13}C NMR (101 MHz, $CDCl_3$) δ 162.22, 160.62, 150.87, 149.96, 140.93, 140.70, 127.90, 127.67, 115.34, 114.70, 111.89, 77.35, 77.03, 76.71, 42.49, 41.79, 31.95, 30.19, 29.72, 29.39, 28.10, 26.96, 23.19, 22.71, 14.19.

*Synthesis of **DASA-18C**:* 1,3-dioctadecylpyrimidine-2,4,6(1*H*,3*H*,5*H*)-trione (compound **2h'**, 2 g, 3 mmol) and dioctadecylamine (compound **3h**, 1.46 g, 3 mmol) were added sequentially to 20 mL of DCM and the color of the solution turned purple immediately. The mixture was heated to 45 ℃ and stirred for 2 h. Solvents were removed by rotary evaporation and further purified by column chromatography to give 1.92 g **DASA-18C** as a purple powder (yield: 56%). ^{1}H NMR (400 MHz, $CDCl_3$) δ 0.81 (t, 24H), 1.19 (td, 189H), 1.55 - 1.63 (m, 9H), 3.29 (t, *J*=7.0 Hz, 5H), 3.84 (t, *J*=5.5 Hz, 6H), 5.96 (t, *J*=12.3 Hz, 1H), 6.63 (d, *J*=12.2 Hz, 1H), 7.04 - 7.12 (m, 2H), 12.57 (s, 1H). ^{13}C NMR (101 MHz, $CDCl_3$) δ 163.88, 162.12, 155.49, 150.40, 149.15, 145.55, 138.69, 101.34, 98.07, 56.54, 48.55, 40.82, 40.64, 30.92, 28.74, 28.70, 28.66, 28.63, 28.60, 28.52, 28.46, 28.42, 28.40, 28.36, 21.68, 13.10.

4. Photoisomerization of DASAs

4.1. Photoisomerization of DASAs in solutions

Due to the push-pull nature of DASAs, the absorption spectra shift in different solutions (**Figure S1-S4**). Introducing alkyl chains does not obviously shift the n-π* absorption band of DASAs. DASAs with various carbon spacers (**DASA-2C**, **DASA-6C**, **DASA-12C**, and **DASA-18C**) show limited isomerization in polar solvents (*e.g.* dichloromethane (DCM) and tetrahydrofuran (THF)) and promoted isomerization in nonpolar solvents (*e.g.* hexane (HEX) and toluene (TOL)) (**Figure S5-S7**).

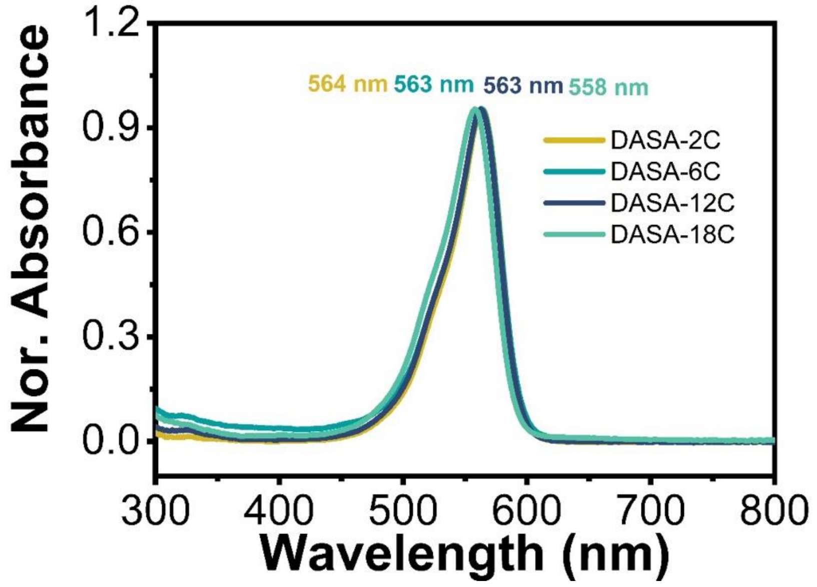

Figure S1. Normalized absorbance spectra of **DASA-2C**, **DASA-6C**, **DASA-12C** and **DASA-18C** in HEX ([DASAs]=0.01 mM).

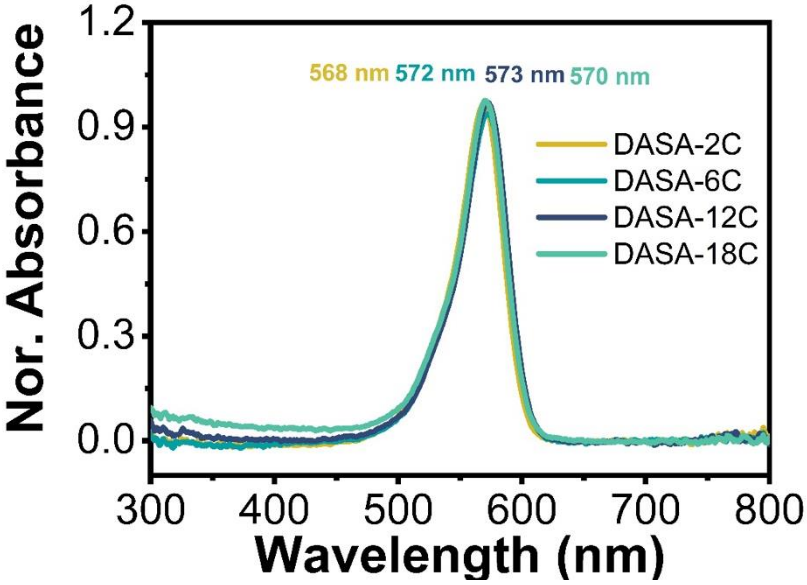

Figure S2. Normalized absorbance spectra of **DASA-2C**, **DASA-6C**, **DASA-12C** and **DASA-18C** in TOL ([DASAs]=0.01 mM).

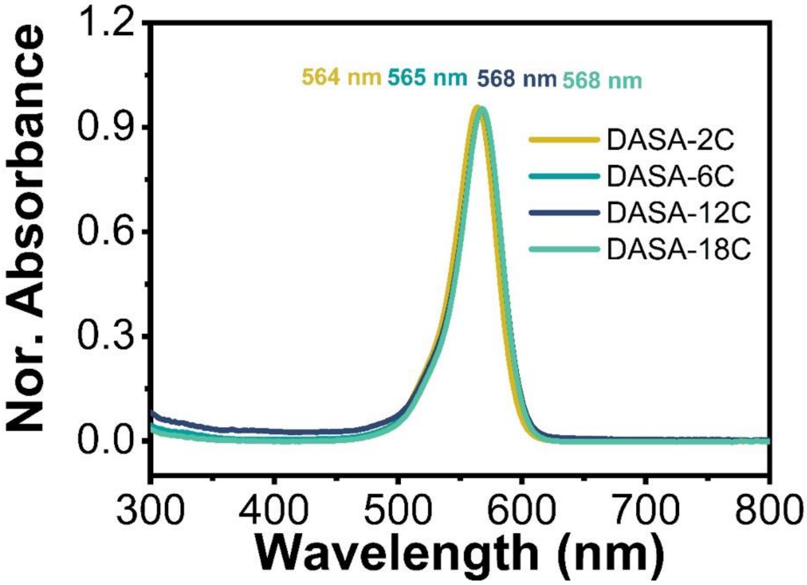

Figure S3. Normalized absorbance spectra of **DASA-2C**, **DASA-6C**, **DASA-12C** and **DASA-18C** in DCM ([DASAs]=0.01 mM).

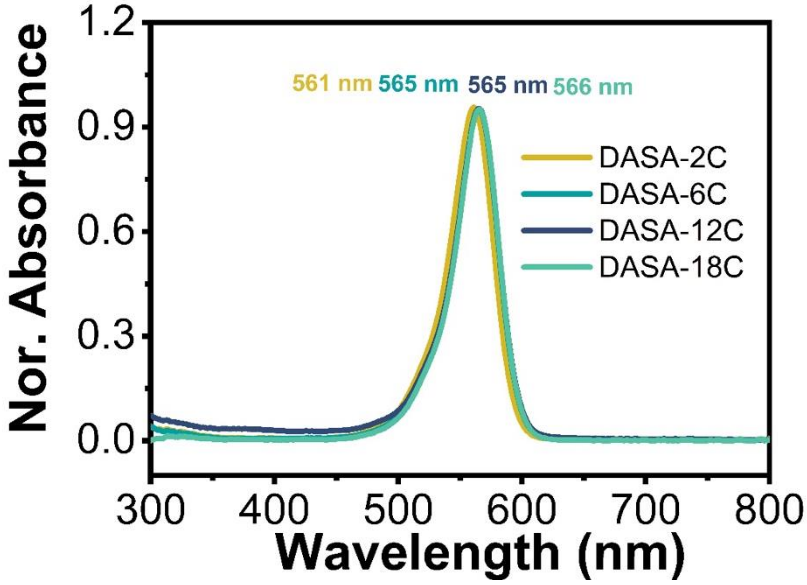

Figure S4. Normalized absorbance spectra of **DASA-2C**, **DASA-6C**, **DASA-12C** and **DASA-18C** in THF [DASAs]=0.01 mM.

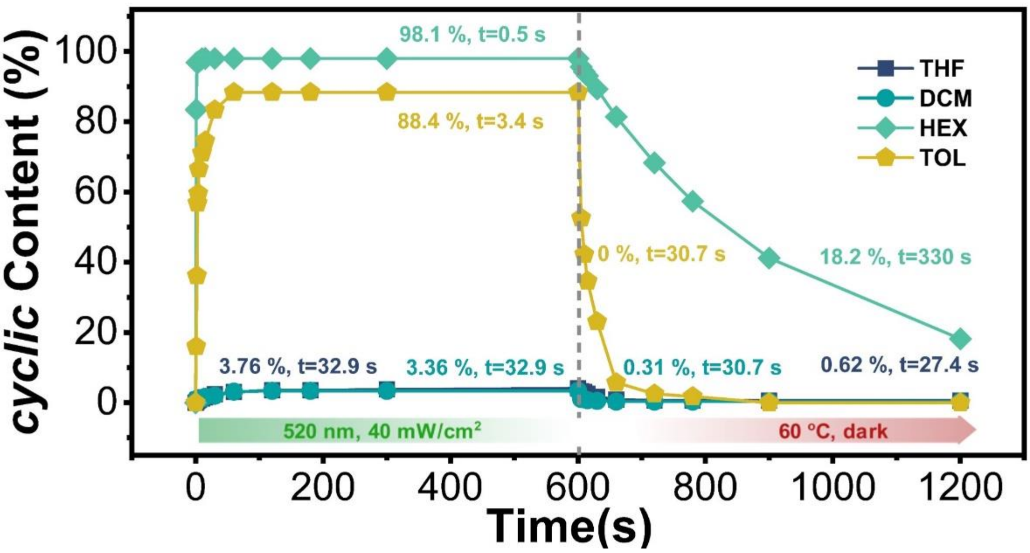

Figure S5. Time-dependent *linear*-to-*cyclic* (left, 520 nm, 40 mW/cm^2) and *cyclic*-to-*linear* (right, in the dark) isomerization of **DASA-2C** in various solvents ([**DASA-2C**]=0.01 mM). The time needed to reach equilibrium is obtained by fitting.

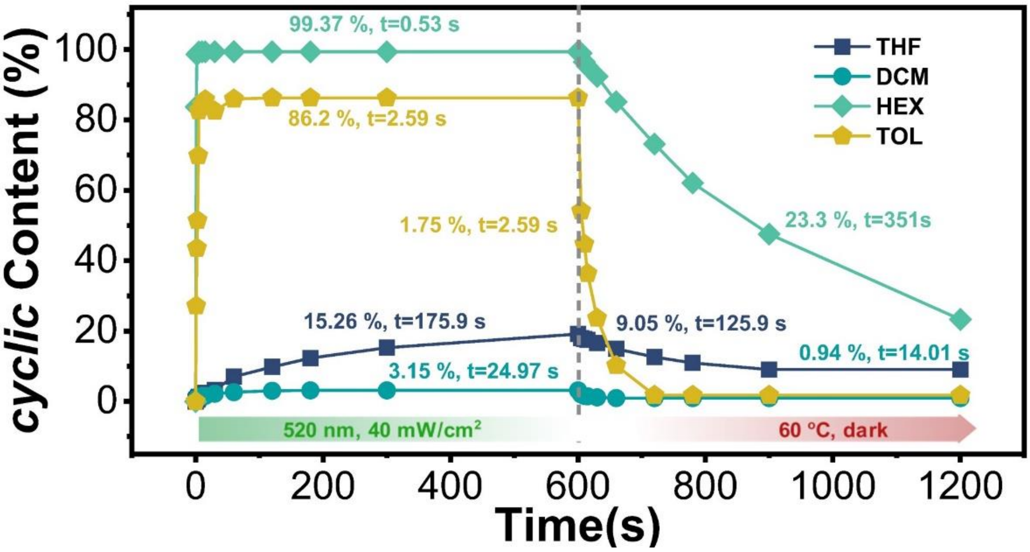

Figure S6. Time-dependent *linear*-to-*cyclic* (left, 520 nm, 40 mW/cm^2) and *cyclic*-to-*linear* (right, in the dark) isomerization of **DASA-6C** in various solvents ([**DASA-6C**]=0.01 mM). The time needed to reach equilibrium is obtained by fitting.

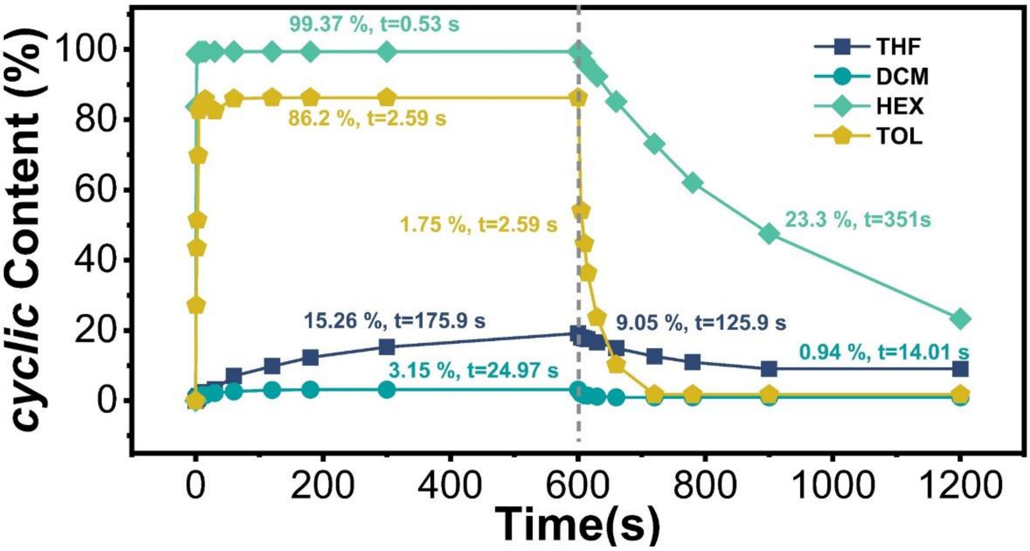

Figure S7. Time-dependent *linear*-to-*cyclic* (left, 520 nm, 40 mW/cm^2) and *cyclic*-to-*linear* (right, in the dark) isomerization of **DASA-18C** in various solvents ([**DASA-18C**]=0.01 mM). The time needed to reach equilibrium is obtained by fitting.

4.2. Photoisomerization of DASAs in the solid state

The isomerization of DASAs in the solid state was investigated by formation of films on the surface of glass substrates via spin-coating at 1000 rpm for 30 s ([DASAs]=0.01 M). A splitting and widening n-π* absorption band at ~540-650 nm was observed for **DASA-2C** in the solid state, which is attributed to the strong intermolecular π-π aggregation (**Figure S8-S9**). The n-π* absorption band gradually narrows with prolonging the carbon spacers for **DASA-6C** and **DASA-12C**, indicating the intermolecular π-π aggregation is inhibited (**Figure S10-S13**). On the other hand, the n-π* absorption band splits and broadens again by further prolonging the carbon spacers for **DASA-18C**, which might be attributed to the crystallization on surface (**Figure S14-S15**).

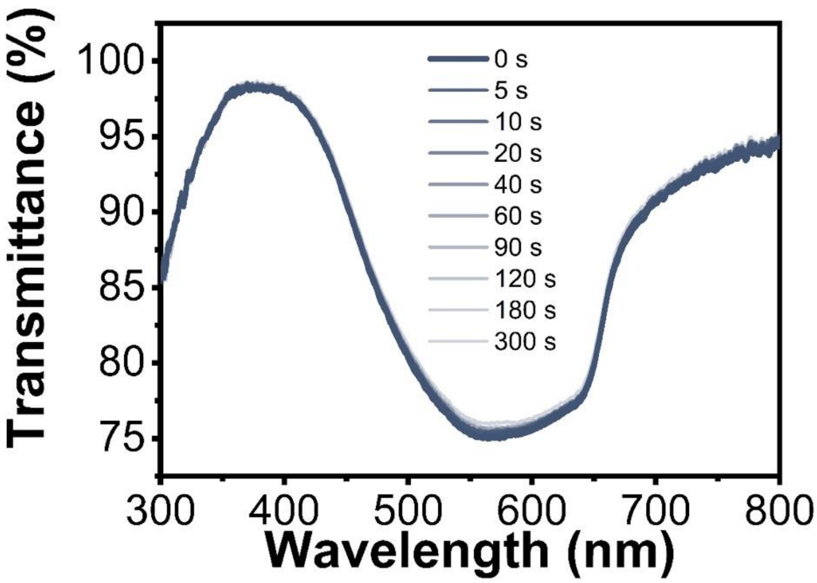

Figure S8. UV-vis transmittance spectra of **DASA-2C** on the glass surface under green light irradiation (520 nm, 40 mW/cm^2) at different time intervals.

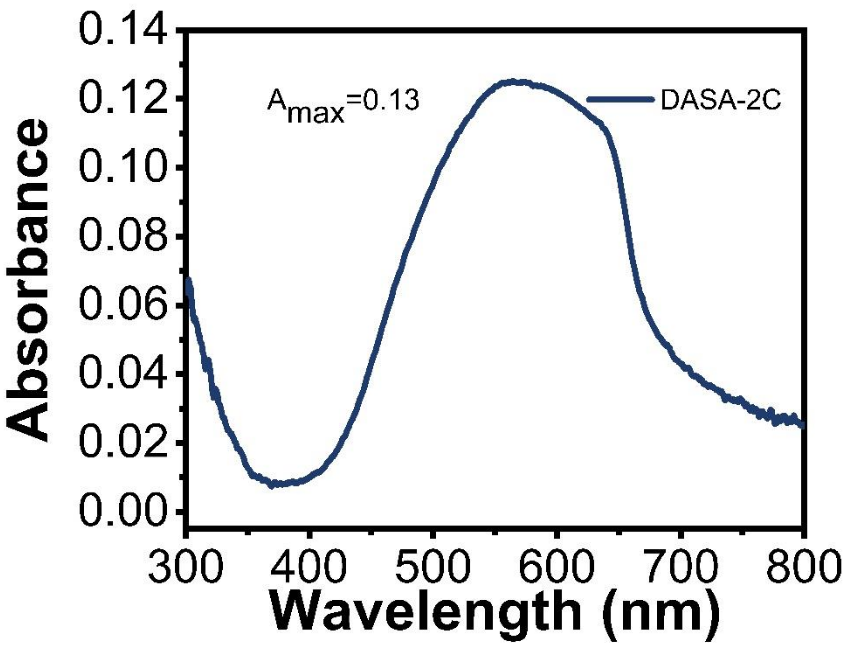

Figure S9. Absorbance spectrum of the film of **DASA-2C** on silicon wafer substrate surface.

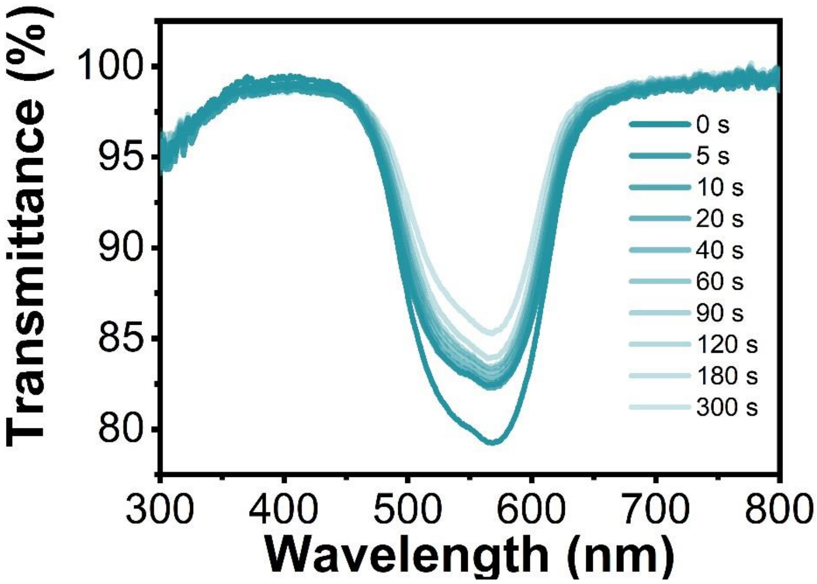

Figure S10. UV-vis transmittance spectra of **DASA-6C** on the glass surface under green light irradiation (520 nm, 40 mW/cm^2) at different time intervals.

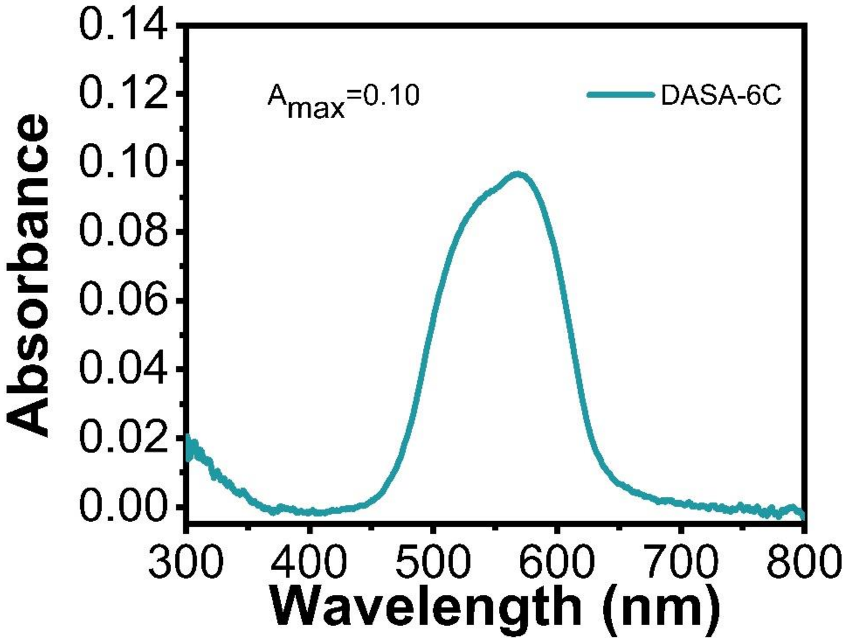

Figure S11. Absorbance spectrum of the film of **DASA-6C** on silicon wafer substrate surface.

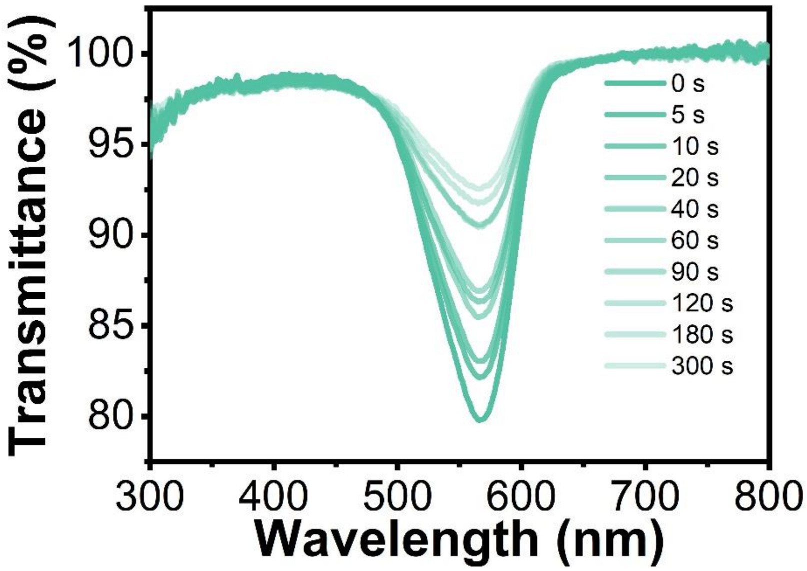

Figure S12. UV-vis transmittance spectra of **DASA-12C** on the glass surface under green light irradiation (520 nm, 40 mW/cm^2) at different time intervals.

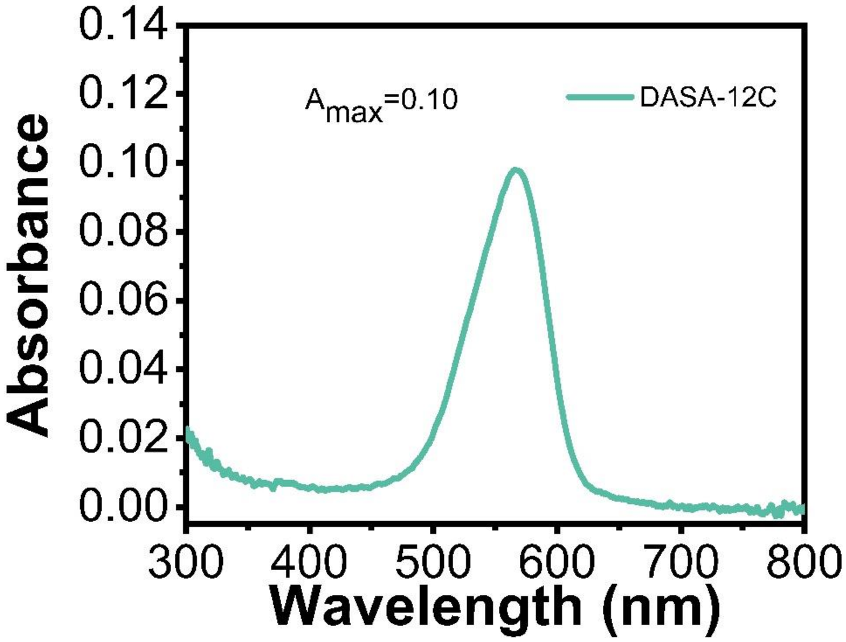

Figure S13. Absorbance spectrum of the film of **DASA-12C** on silicon wafer substrate surface.

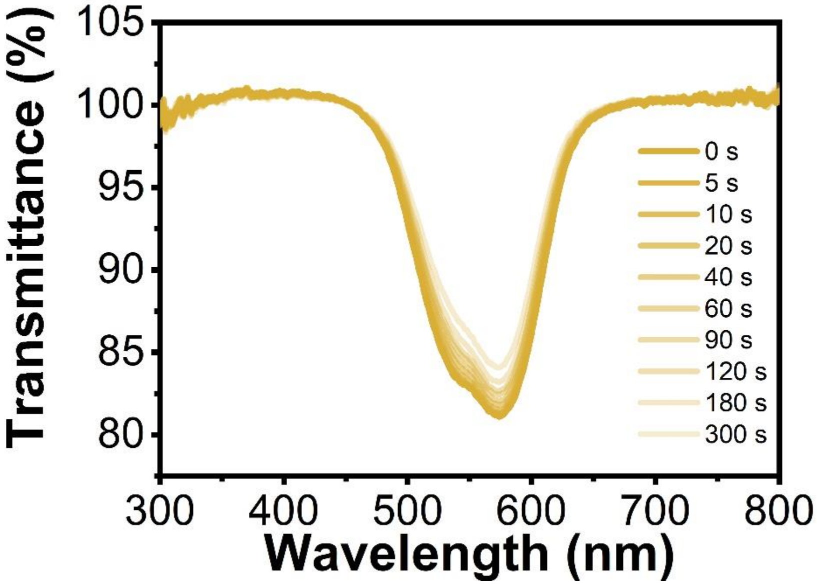

Figure S14. UV-vis transmittance spectra of **DASA-18C** on the glass surface under green light irradiation (520 nm, 40 mW/cm^2) at different time intervals.

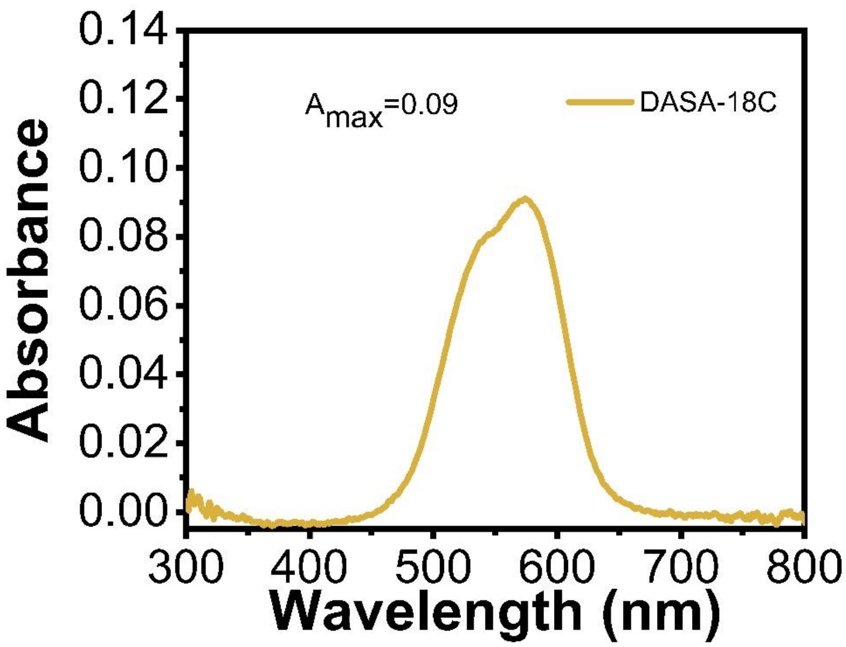

Figure S15. Absorbance spectrum of the film of **DASA-12C** on silicon wafer substrate surface.

5. Mechanical properties of DASAs films

The thickness of the DASAs films were determined by AFM, and the information has been summarized in **Figure S16** and **Table S1**. However, due to the poor film-forming property of **DASA-2C**, which generates plenty of fragments on surface, the thickness is therefore not provided. On the other hand, the films of **DASA-6C**, **DASA-12C** and **DASA-18C** exhibit similar thickness ranged between ~70 and ~100 nm.

Table S1. Summarized thickness of the films of DASAs on silicon wafer substrates surface measured by AFM.

	Mean (nm)	Min (nm)	Max (nm)
**DASA-2C**	**-**	-	-
**DASA-6C**	69.38	44.33	75.33
**DASA-12C**	99.32	96.33	103.7
**DASA-18C**	88.57	79.33	101

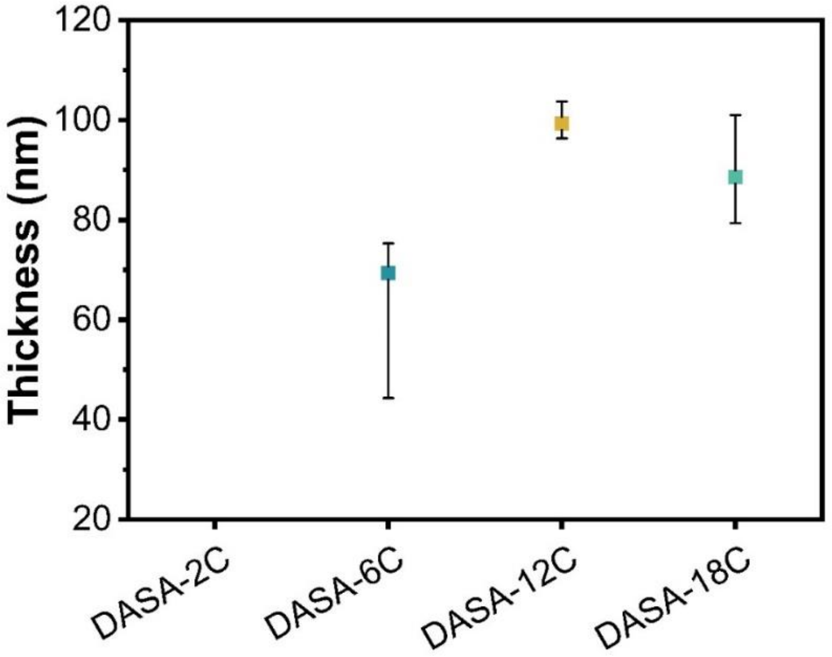

Figure S16. Thickness of the films of DASAs on silicon wafer substrates surface measured by AFM.

High-resolution AFM measurements were implemented with rectangular silicon nitride (Si_3N_4) cantilevers (RTESPA-150, Bruker, USA) with tapered silicon tips. The spring constant of each cantilever (~ 5 N/m) was calibrated on the sapphire substrate (SAPPHIRE-12M, Bruker, USA) with the thermal noise method in air at room temperature (24 ± 1 °C).[9, 10] The topographical characterization of the sample was performed in the PeakForce QNM mode with a scanning force of 15 nN. AFM-based mechanical tests were operated with the "force volume" function and the indenting force in the mechanical experiments was kept identical, *i.e.*, 15 nN, to ensure consistent testing conditions. The captured images were analyzed via the AFM in-built offline processing software (NanoScope Analysis). Because of the small load in experiments and the large adhesion between the cantilever tip and the sample, the Young's modulus can be calculated based on the JKR elastic contact model,[11, 12] *i.e.*, solving the following equations,

$$\begin{cases} a^3 = \frac{R}{E^*}(F + 3\pi wR + \sqrt{6\pi wRF + (3\pi wR)^2}) \\ \delta = \frac{a^2}{3R} + \frac{2F}{3aE^*} \end{cases} \quad (1)$$

where a is the contact radius of probe and sample, R is the tip radius, w is the adhesion energy, F is the loading force, δ is the indentation depth, E^* is the reduced Young's modulus. And $\frac{1}{E^*} = \frac{1-\nu_{sample}^2}{E_{sample}} + \frac{1-\nu_{tip}^2}{E_{tip}}$, ν, E are Poisson's ratio and Young's modulus, respectively. Since $E_{tip} \gg E_{sample}$, $\frac{1}{E^*} \approx \frac{1-\nu_{sample}^2}{E_{sample}}$.

Force-distance curves of the DASAs films on silicon wafer substrates were summarized (**Figure S17-S20**).

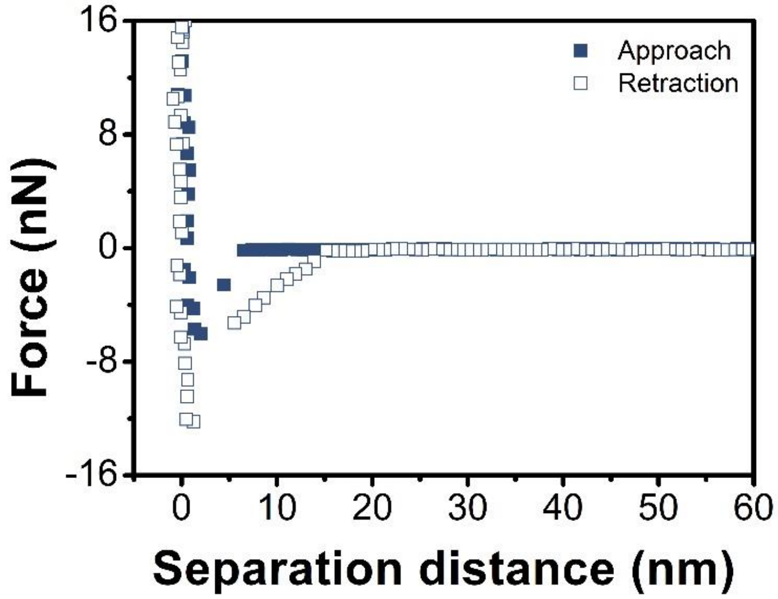

Figure S17. Force-distance curve of the **DASA-2C** films on silicon wafer substrates surface.

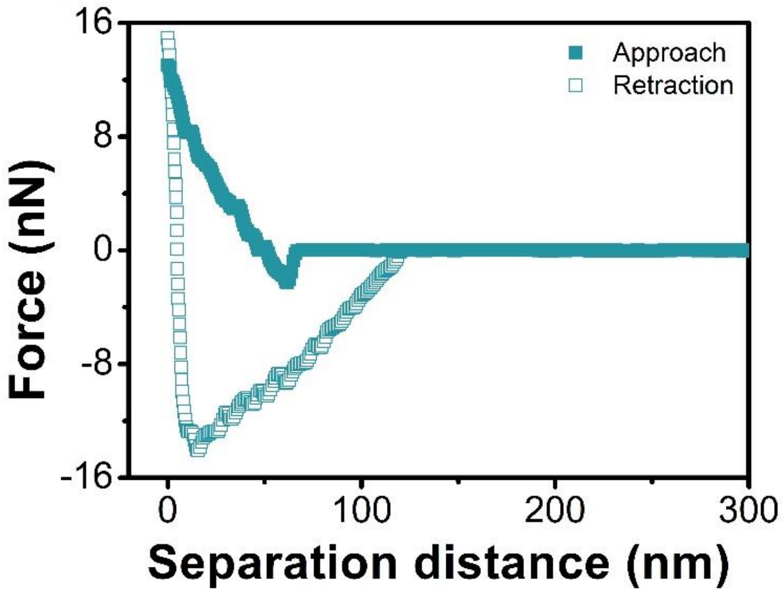

Figure S18. Force-distance curve of the **DASA-6C** films on silicon wafer substrates surface.

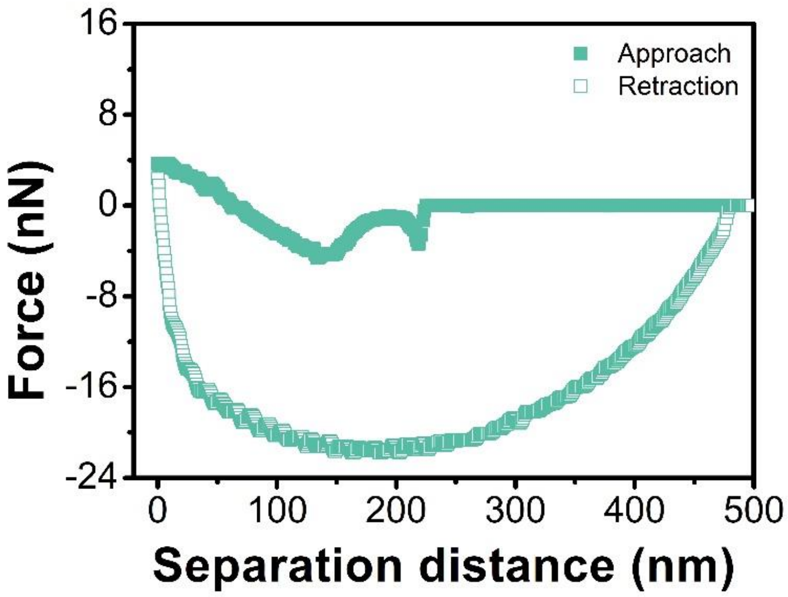

Figure S19. Force-distance curve of the **DASA-12C** films on silicon wafer substrates surface.

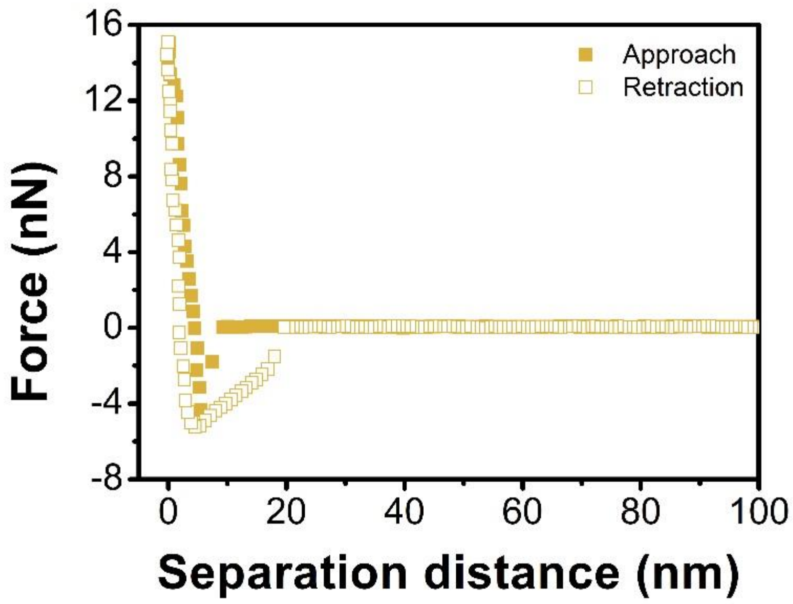

Figure S20. Force-distance curve of the **DASA-18C** films on silicon wafer substrates surface.

6. Fabrication of 2D logic-in-memory devices

The 2D logic-in-memory devices were fabricated via the following procedure:

(1) Monolayer graphene/h-BN/sublayer graphene were picked up by polymer films composed of polycarbonate (PC)/poly-dimethylsiloxane (PDMS) and then deposited on silicon substrates.

(2) The polymer films were dissolved and washed with trichloromethane (CHCl3).

(3) Electrodes were deposited on the heterostructure surface using the photomask lithography and metal thermal evaporation/lift-off process.

(4) DASAs were spin-coated on the surface of graphene.

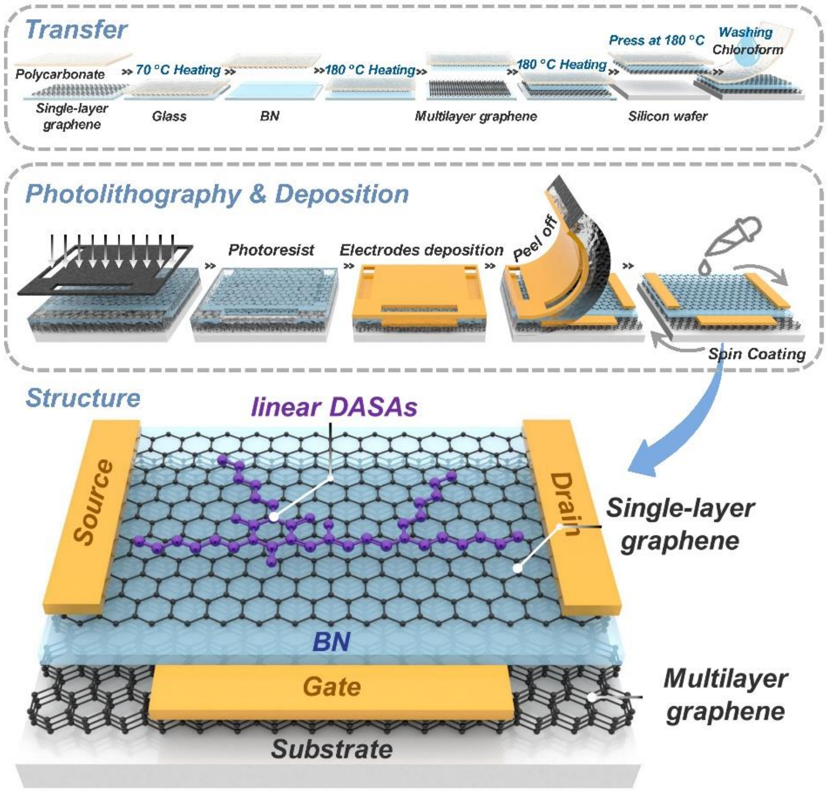

Scheme S5. Schematic illustration of the fabrication of the 2D logic-in-memory device.

In detail, h-BN and graphene were exfoliated on the surface of silicon substrates (thickness: 285 nm) using adhesive tapes. The transfer film is composed of a PC supported by PDMS. Use the viscosity of PC to pick up monolayer graphene at 70℃ for 3-10 min, and the BN/sublayer graphene was picked up with the same method at 180 °C, which was laid under the monolayer graphene to obtain the heterostructure onto a SiO_2. After cooled to room temperature, the outmost polycarbonate layer was dissolved and washed by chloroform. The Cr/Au (5 nm/40 nm) electrodes were deposited on the heterostructure surface using the photomask lithography and metal thermal evaporation (evaporation rate: Cr 3 Å/s and Au 1 Å /s; the pressure of vacuum is 9×10^{-5}) and the device was obtained after peeling off the photoresist layer. Afterwards, DASAs films were formed on the surface of devices by spin-coating the solutions ([DASAs]=0.03 mM in chloroform) at 1000 rpm for 30 s (**Scheme S5**).

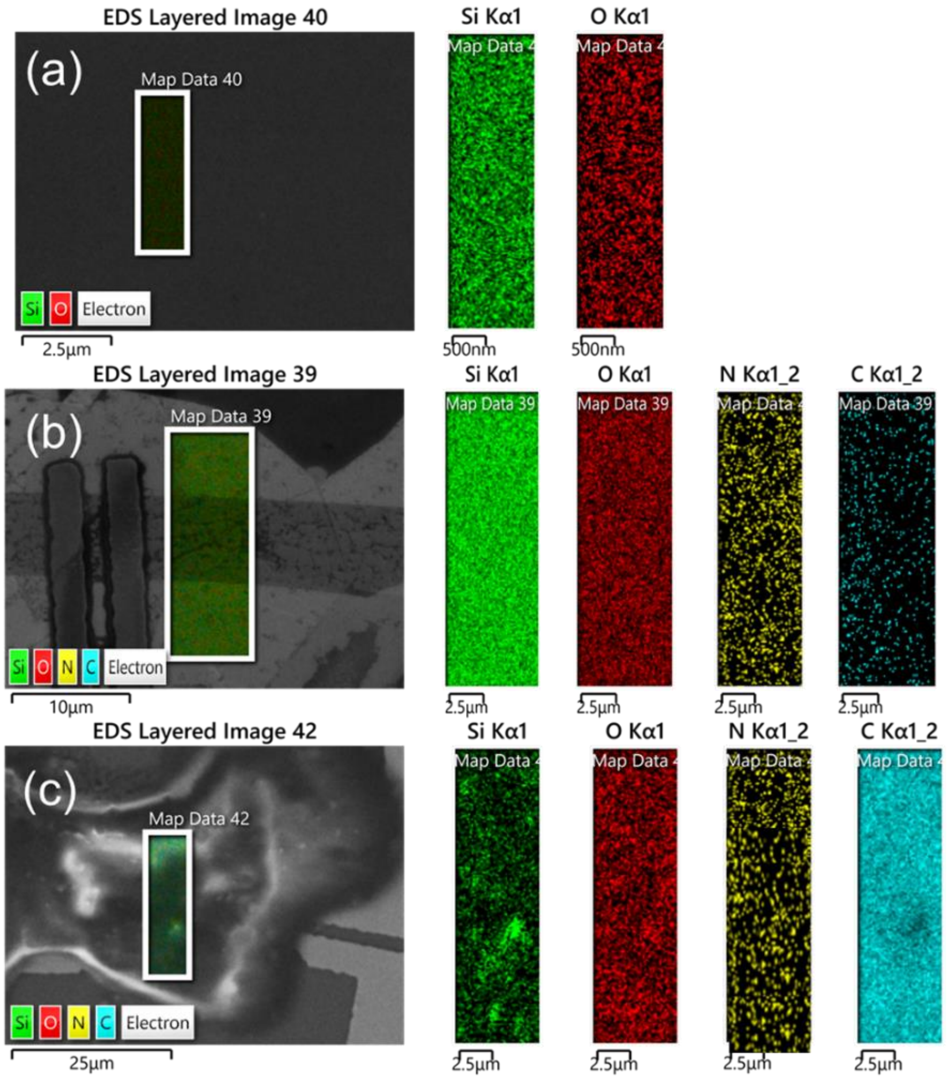

Figure S21. SEM images and EDS mapping on the surface of silicon wafer substrates (a), 2D devices without DASAs (b), and 2D logic-in-memory devices with **DASA-12C** (c), scale bar: 2.5 μm.

7. Light-controlling the 2D logic-in-memory devices

Depositing of DASAs on the surface of 2D devices shifts the I_{ds}-V_g curves, indicating the interfacial effect and intermolecular interaction between graphene and DASAs (**Figure S22-S25**). The 2D logic-in-memory devices exhibit negatively shifted V_g and gradually increased I_{ds} upon irradiation while using **DASA-6C**, **DASA-12C,** and **DASA-18C** as the photoswitches (**Figure S26-S27**). As expected, the I_{ds}-V_g curves of the devices with **DASA-2C** does not shift after irradiation due to the strong intermolecular π-π stacking (**Figure S29**).

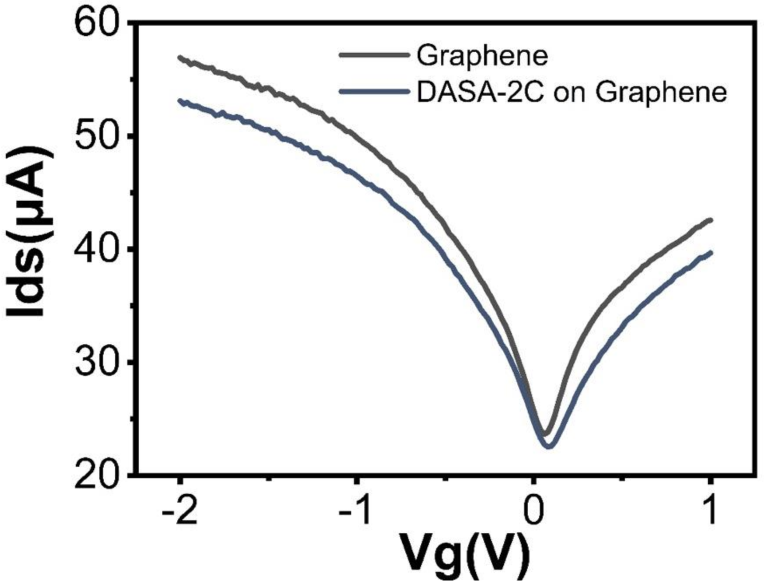

Figure S22. I_{ds}-V_g curves of the 2D devices without DASAs (black) and with **DASA-2C** (navy blue).

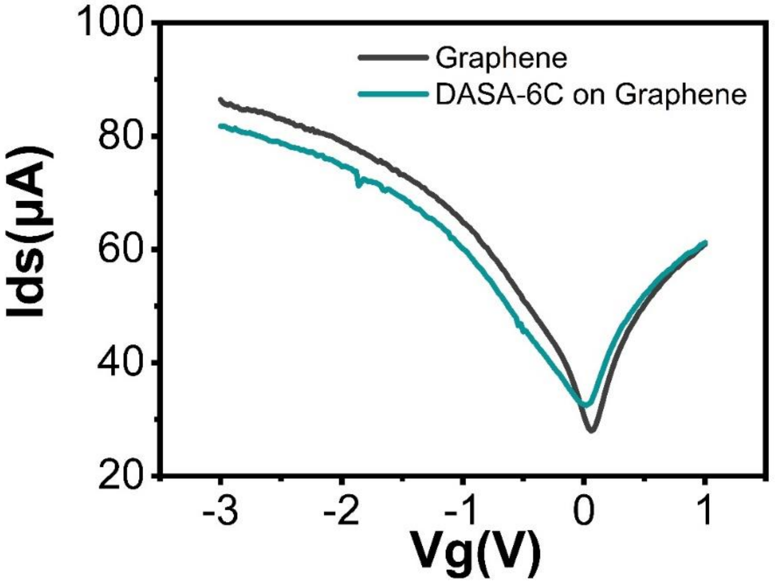

Figure S23. I_{ds}-V_g curves of the 2D devices without DASAs (black) and with **DASA-6C** (navy blue).

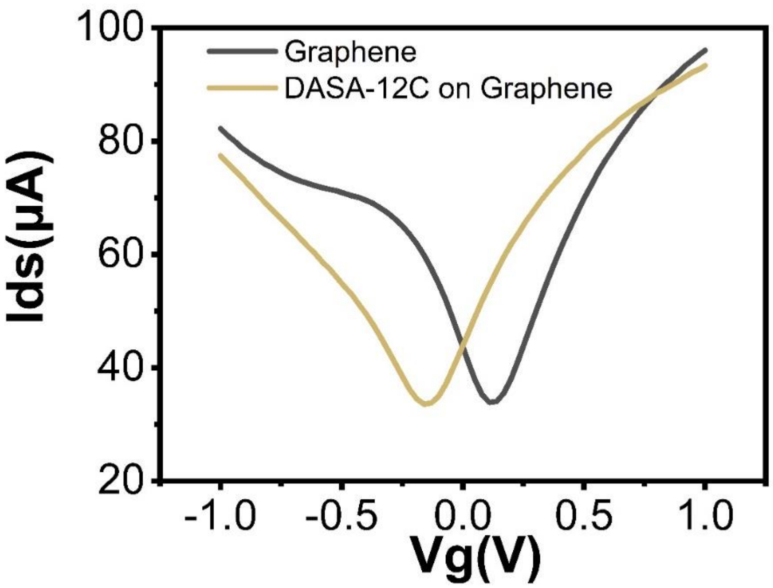

Figure S24. I_{ds}-V_g curves of the 2D devices without DASAs (black) and with **DASA-12C** (yellow).

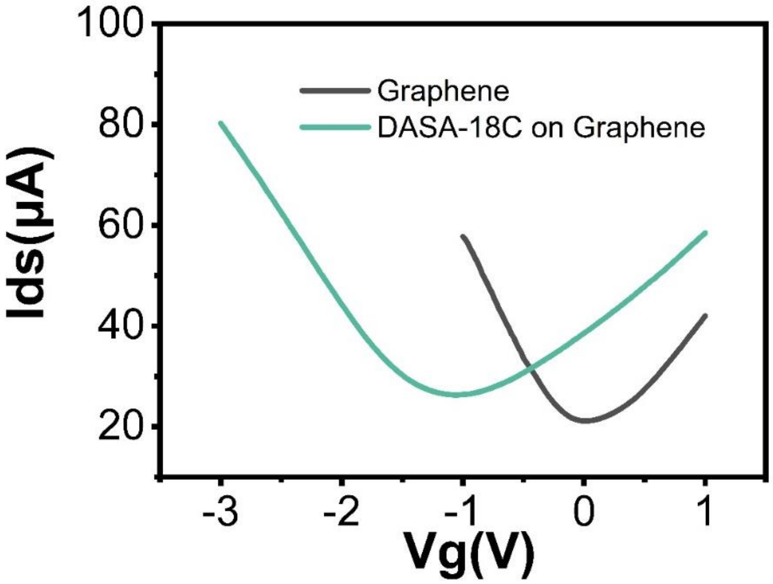

Figure S25. I_{ds}-V_g curves of the 2D devices without DASAs (black) and with **DASA-18C** (green).

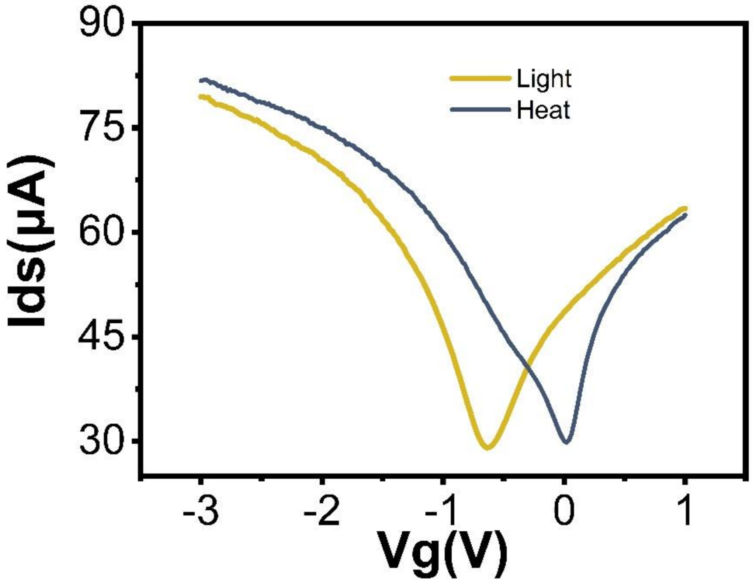

Figure S26. I_{ds}-V_g curves of the devices with **DASA-6C** before (navy blue) and after (yellow) green light irradiation (100 mW/cm^2).

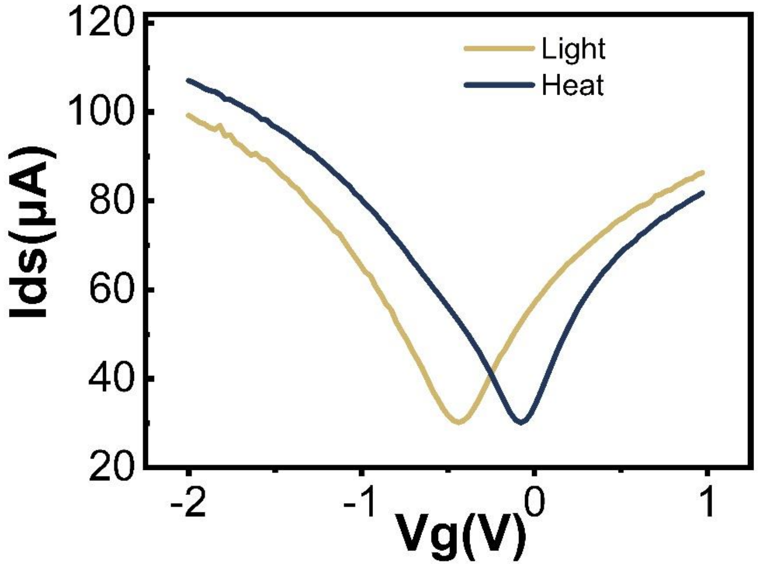

Figure S27. I_{ds}-V_g curves of the devices with **DASA-12C** before (navy blue) and after (yellow) green light irradiation (100 mW/cm^2).

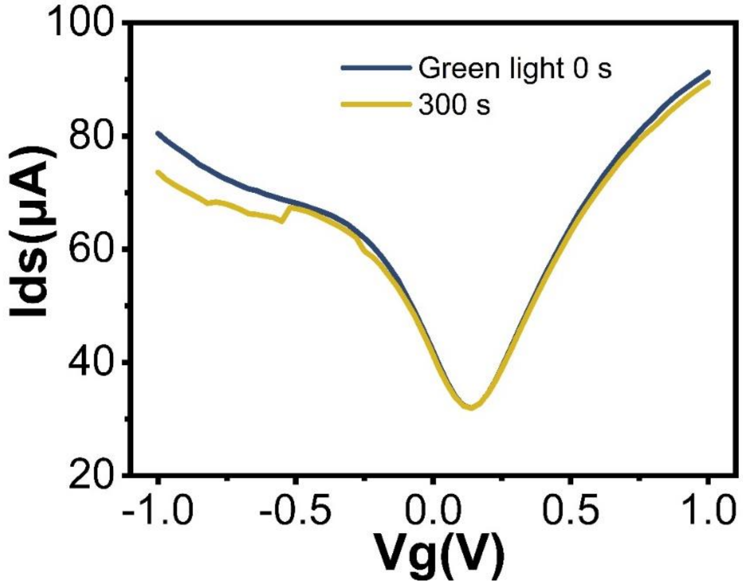

Figure S28. I_{ds}-V_g curves of the 2D devices without DASAs before (navy blue) and after green (yellow) light irradiation (520 nm, 125 mW/cm^2).

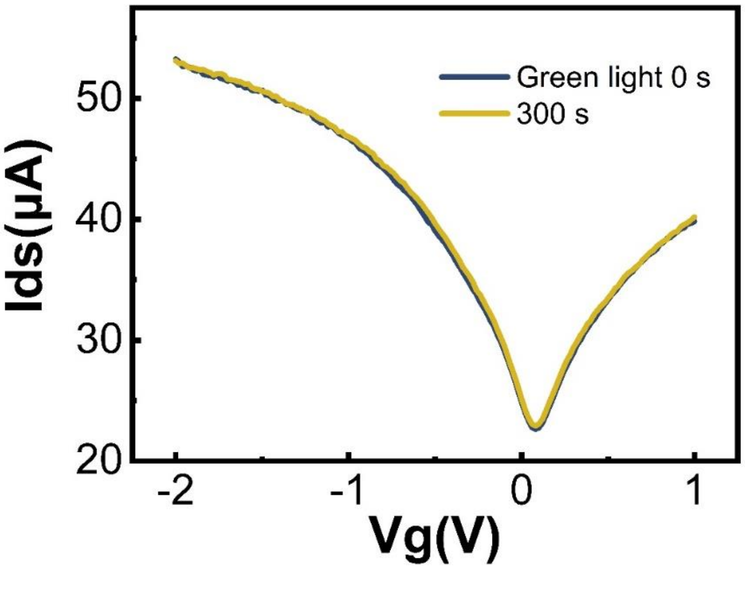

Figure S29. I_{ds}-V_g curves of the devices with **DASA-2C** before (navy blue) and after (yellow) green light irradiation (100 mW/cm^2).

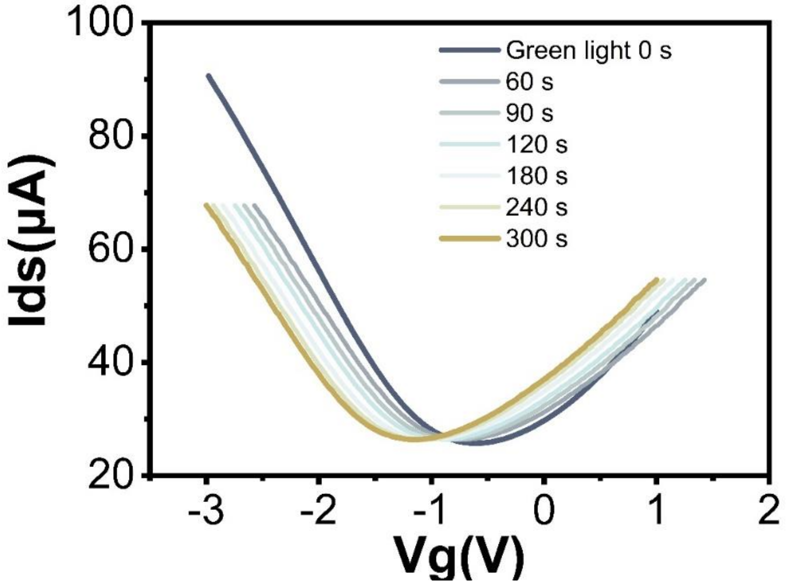

Figure S30. I_{ds}-V_g curves of the devices with **DASA-18C** after green light irradiation (40 mW/cm^2).

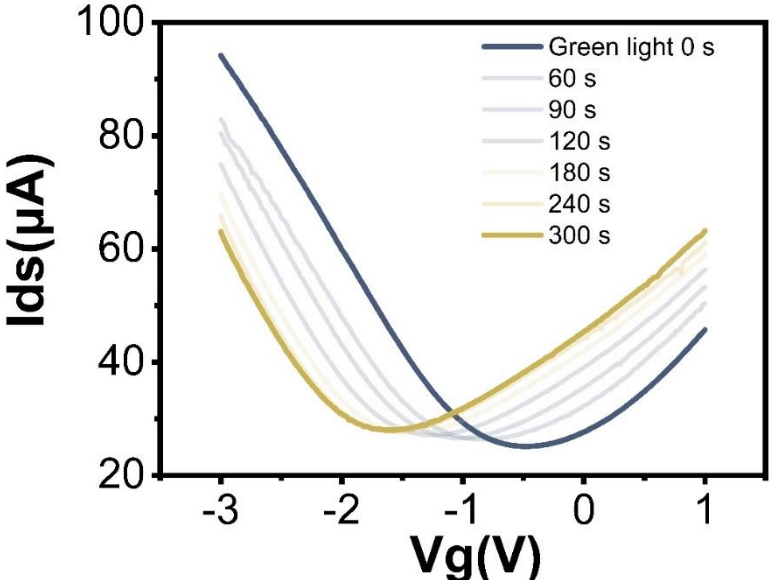

Figure S31. I_{ds}-V_g curves of the devices with **DASA-18C** after green light irradiation (70 mW/cm^2).

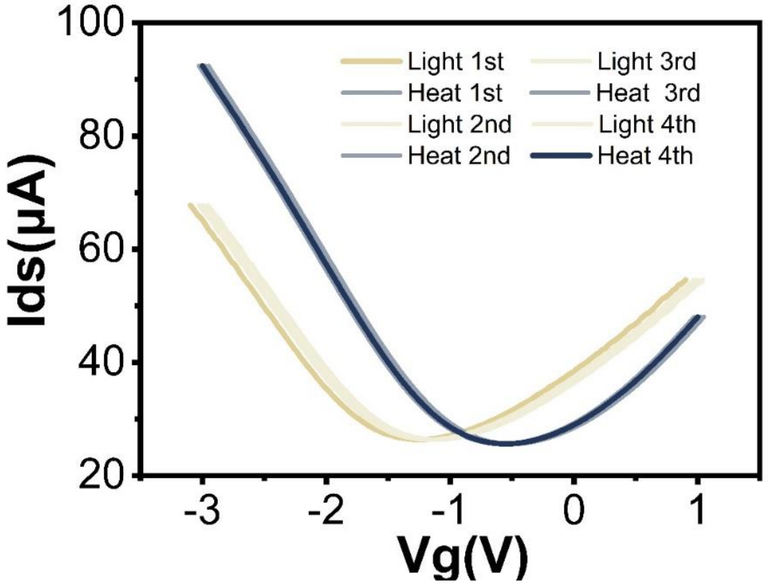

Figure S32. I_{ds}-V_g curves of the devices with **DASA-18C** after repeated treatment of green light (40 mW/cm^2, 300 s) and heat (40 ^{o}C, 300 s).

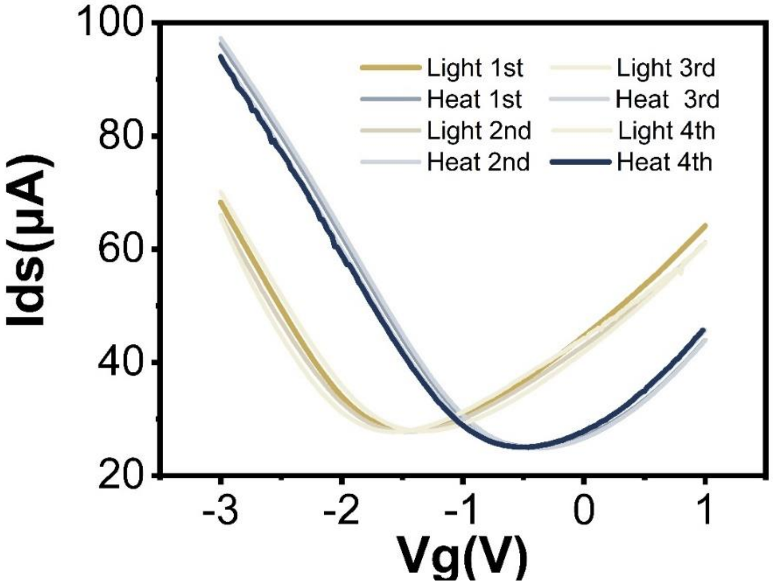

Figure S33. I_{ds}-V_g curves of the devices with **DASA-18C** after repeated treatment of green light (70 mW/cm^2, 300 s) and heat (40 ^{o}C, 300 s).

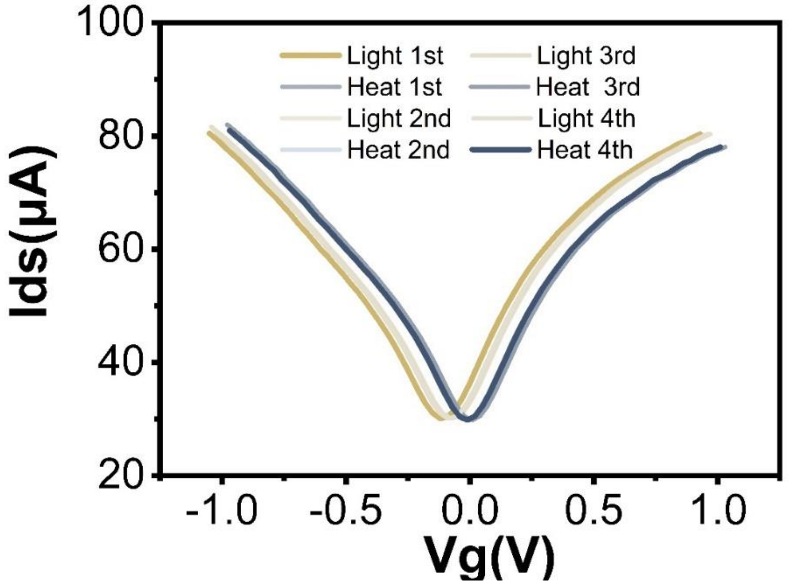

Figure S34. I_{ds}-V_g curves of the devices with **DASA-12C** after repeated treatment of green light (40 mW/cm^2, 300 s) and heat (40 ^oC, 300 s).

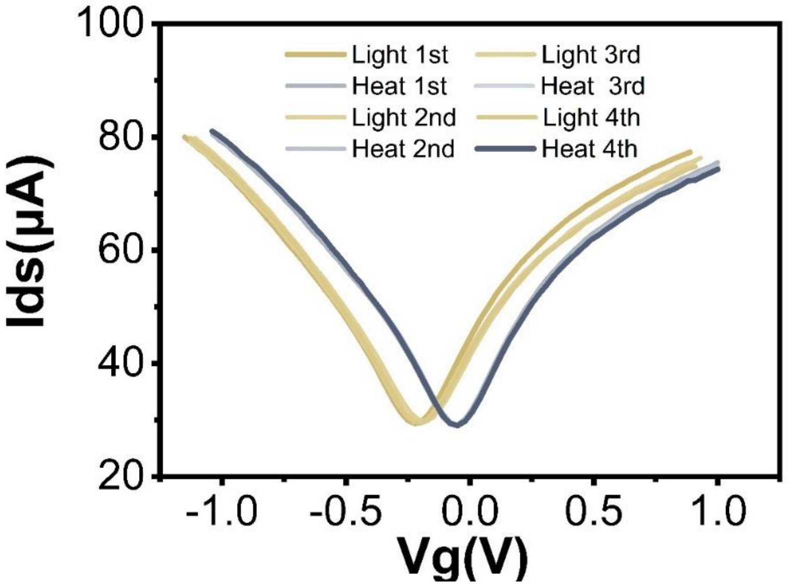

Figure S35. I_{ds}-V_g curves of the devices with **DASA-12C** after repeated treatment of green light (70 mW/cm^2, 300 s) and heat (40 ^oC, 300 s).

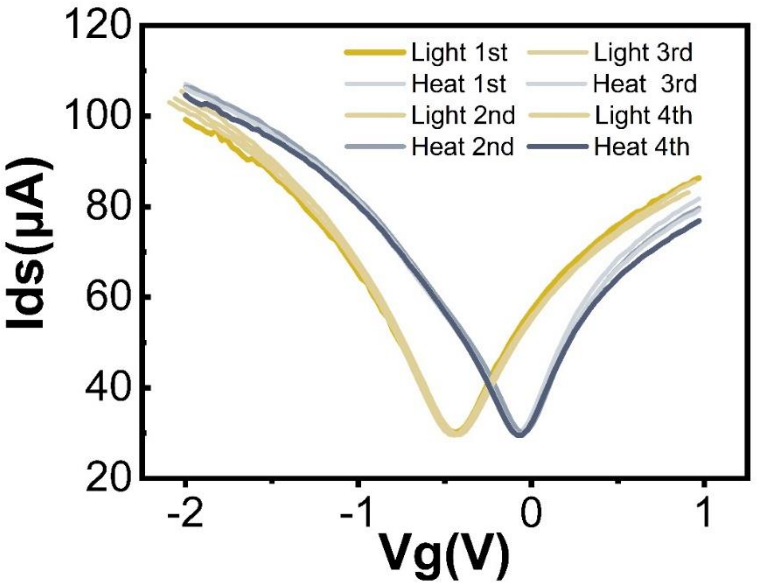

Figure S36. I_{ds}-V_g curves of the devices with **DASA-12C** after repeated treatment of green light (100 mW/cm^2, 300 s) and heat (40 ^{o}C, 300 s).

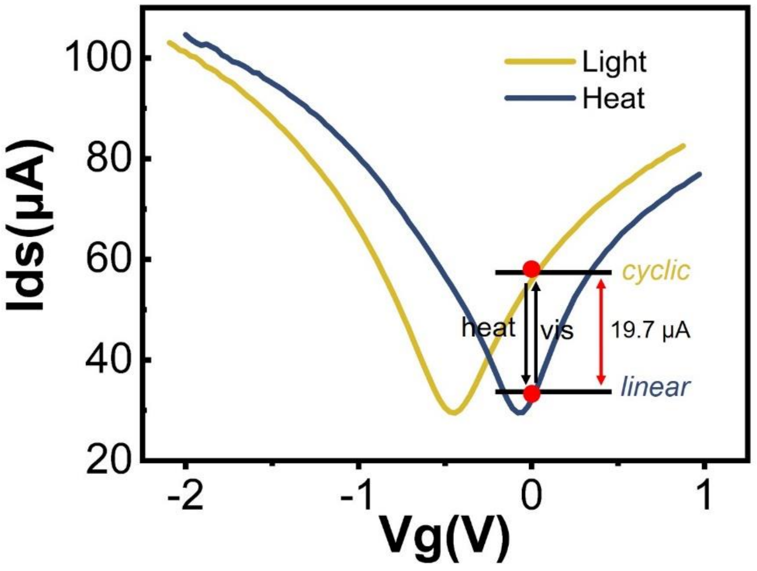

Figure S37. I_{ds}-V_g curves of the devices with **DASA-12C** after repeated treatment of green light (100 mW/cm^2, 300 s) and heat (40 ^{o}C, 300 s). The red double arrow indicates the range of the dynamic doping control window.

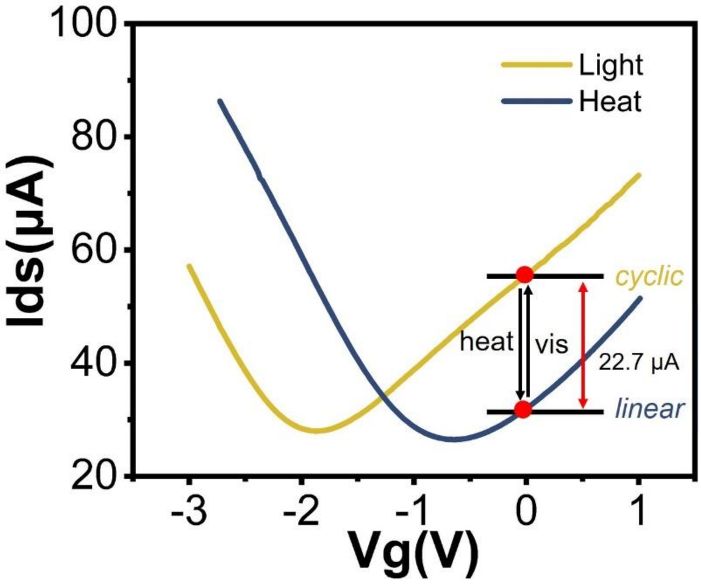

Figure S38. I_{ds}-V_g curves of the devices with **DASA-12C** after repeated treatment of green light (100 mW/cm^2, 300 s) and heat (40 ^{o}C, 300 s). The red double arrow indicates the range of the dynamic doping control window.

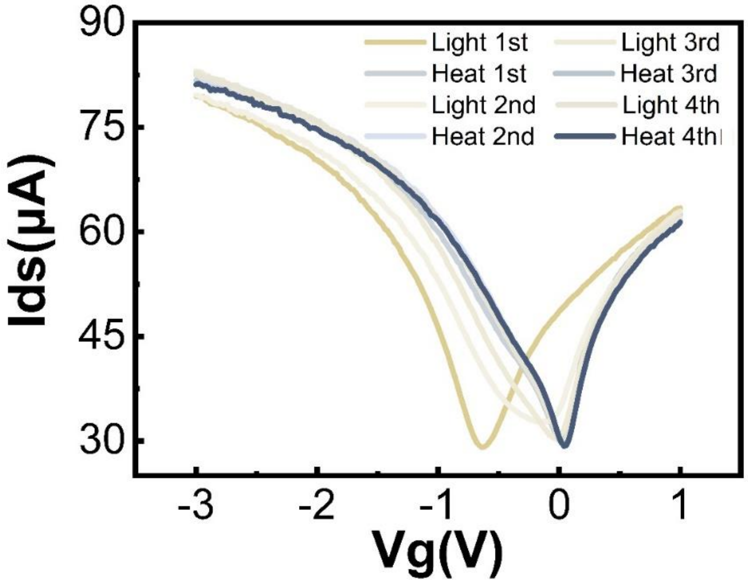

Figure S39. I_{ds}-V_g curves of the devices with **DASA-6C** after repeated treatment of green light (100 mW/cm^2, 300 s) and heat (40 ^{o}C, 300 s).

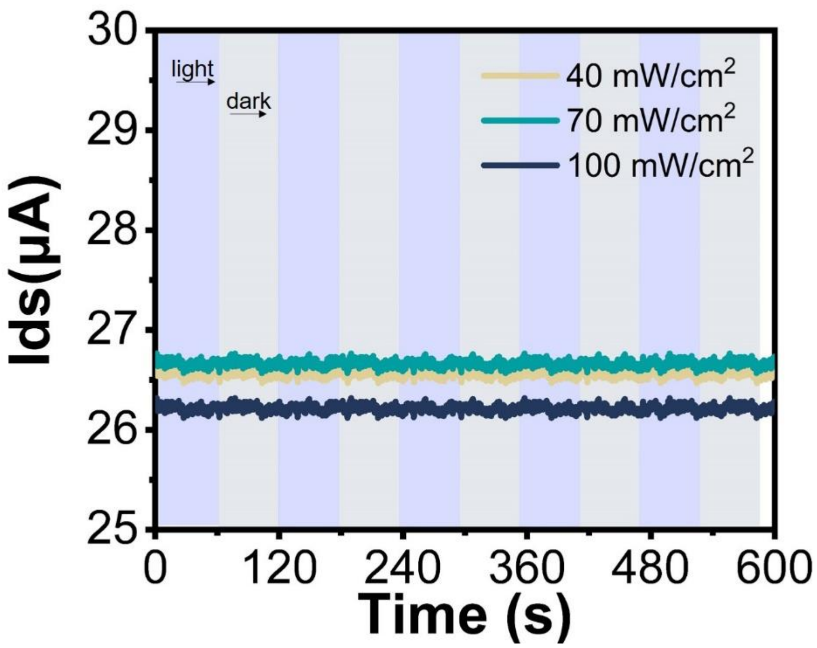

Figure S40. Time-dependent I_{ds} variation of the devices with **DASA-2C** under green light irradiation with different intensities (periodically treated by light for 60 s and dark for 60 s).

Table S2. Summarized I_{ds} of logic-in-memory devices of **DASA-12C**. The devices were treated by green light irradiation (100 mW/cm^2, 300 s) and heat (40 ^{o}C, 300 s) sequentially.

No.	Fabrication	Pristine I_{ds} (μA)	I_{ds} after green light (μA)	I_{ds} after heat (μA)
Sample-1	Success	33.4	54.2	30.7
Sample-2	Success	32.7	55.6	33.2
Sample-3	Failed	-	-	-
Sample-4	Failed	-	-	-
Sample-5	Success	32.2	55.9	33.3
Sample-6	Success	35.4	54.7	31.6
Sample-7	Failed	-	-	-

Sample-8	Success	33.1	54.1	35.5
Sample-9	Success	31.9	55.1	34.3

8. DFT calculation

The mechanism of DASAs isomerization was understood with the assistance of DFT calculations. The molecular structures of the key intermediates during *linear*-to-*cyclic* isomerization of DASAs were calculated.

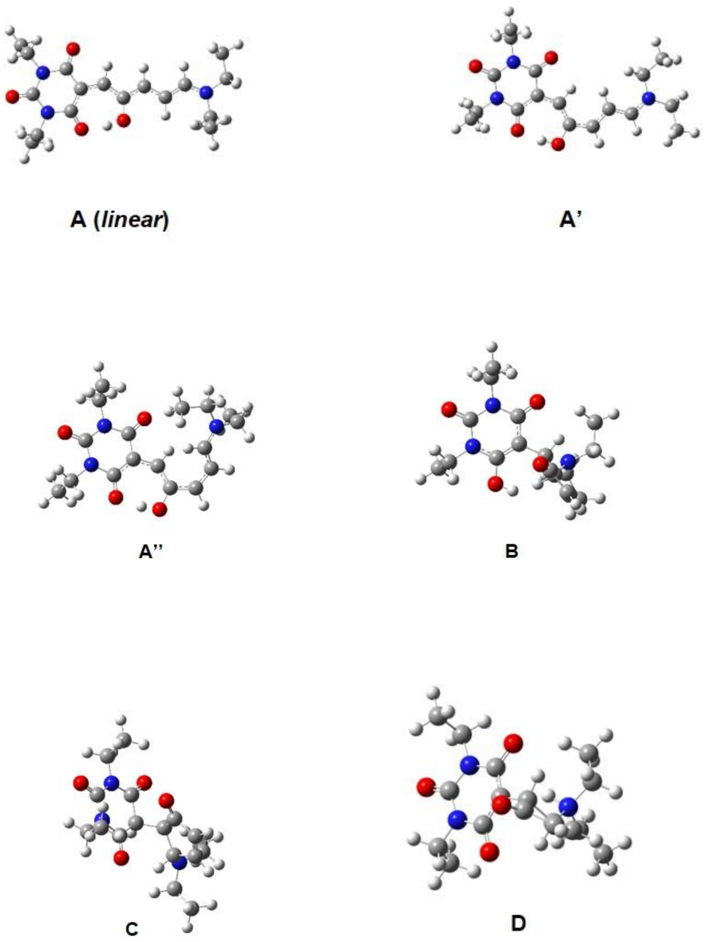

Figure S41. Calculated molecular structures of the 6 intermediates of **DASA-2C** during the *linear*-to-*cyclic* isomerization without graphene.

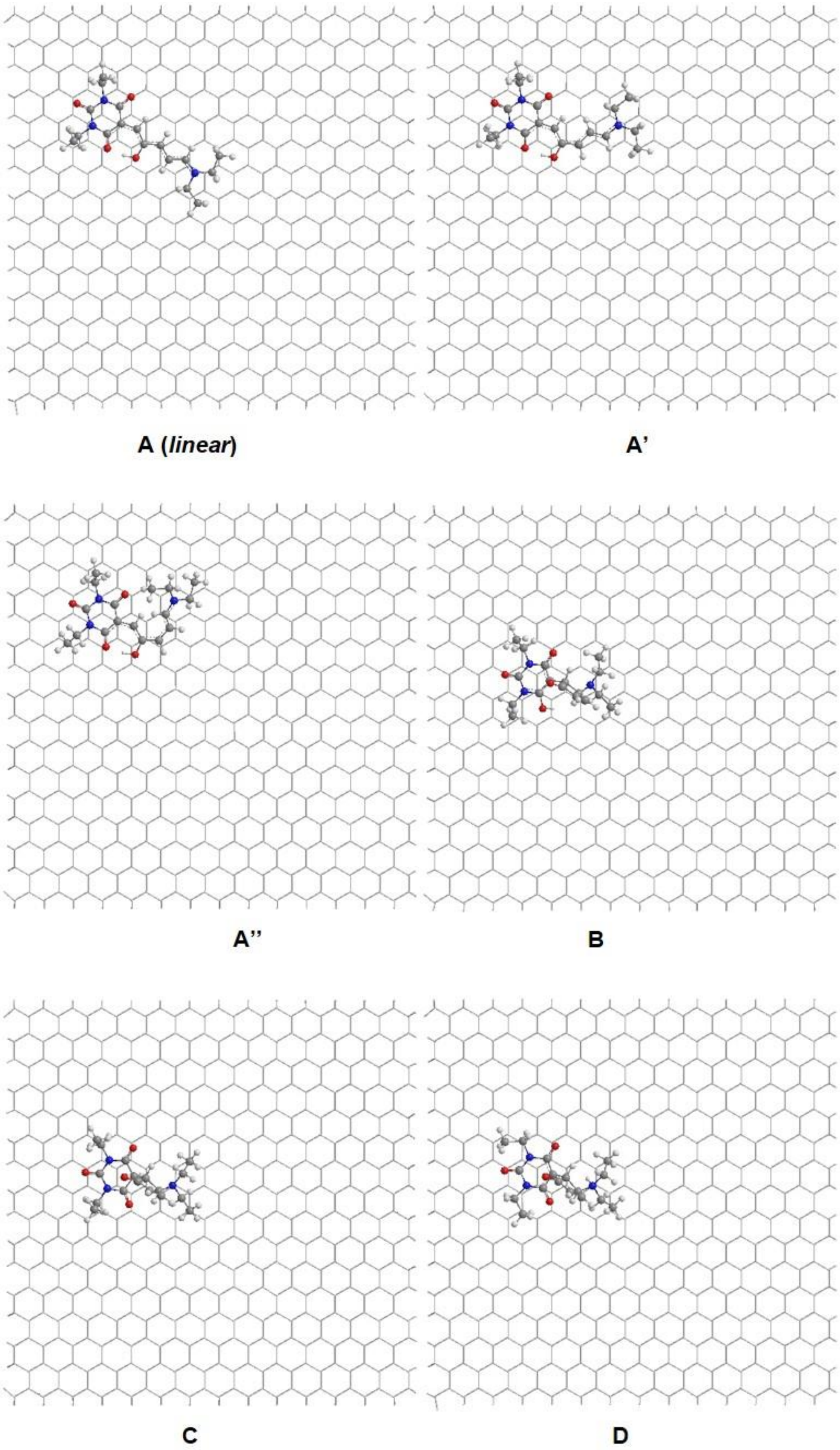

Figure S42. Calculated molecular structures of the 6 intermediates of **DASA-2C** during the *linear*-to-*cyclic* isomerization on the graphene surface.

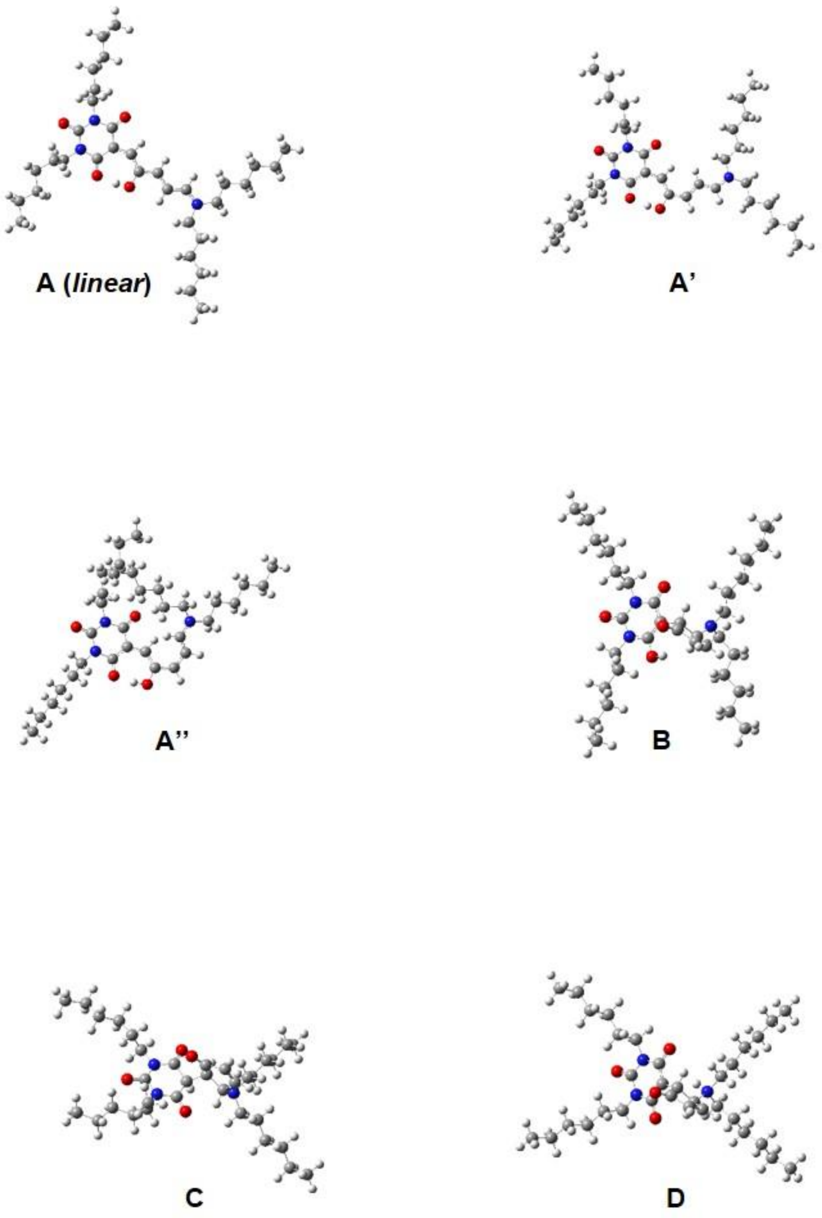

Figure S43. Calculated molecular structures of the 6 intermediates of **DASA-6C** during the *linear*-to-*cyclic* isomerization without graphene.

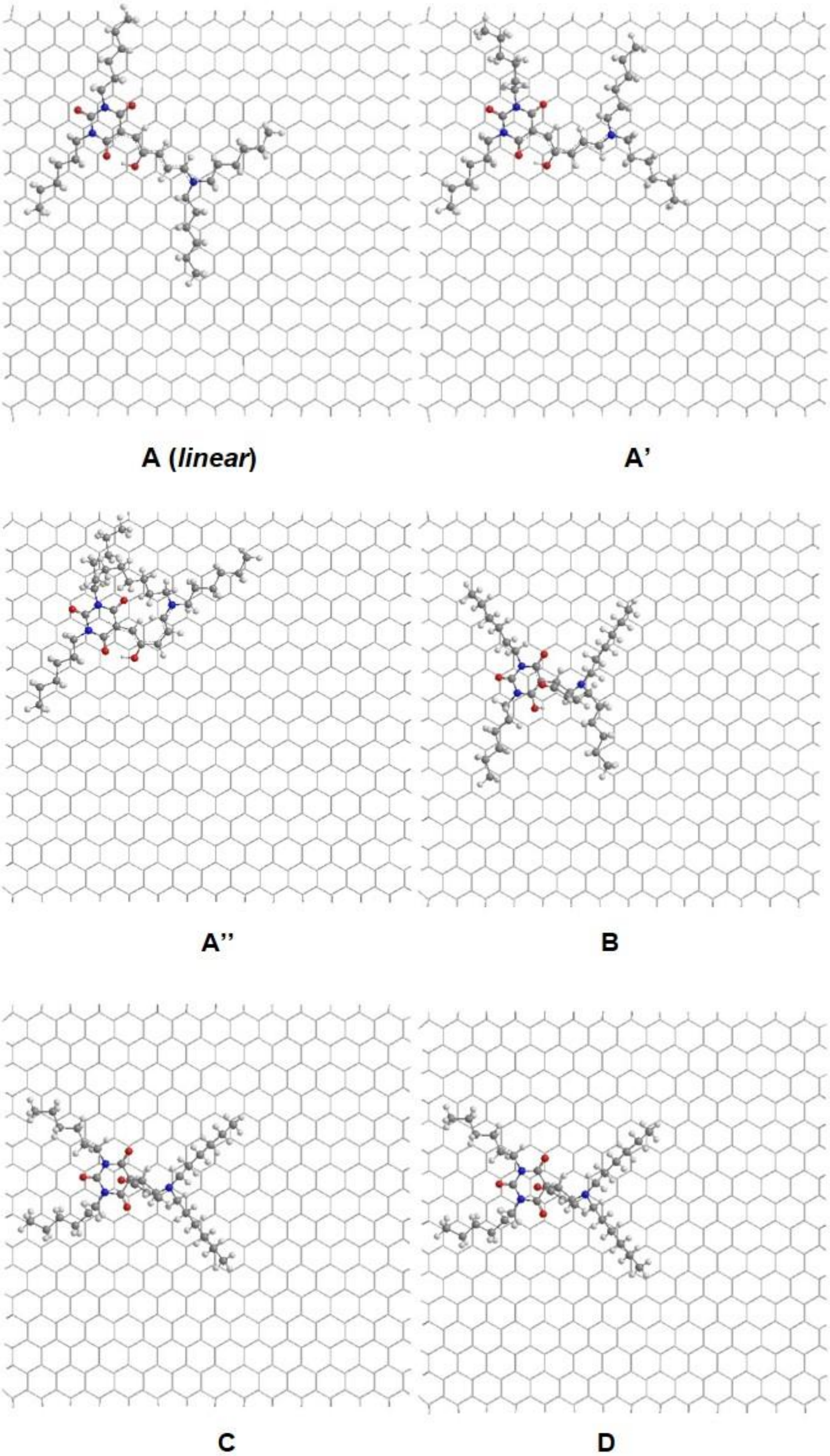

Figure S44. Calculated molecular structures of the 6 intermediates of **DASA-6C** during the *linear*-to-*cyclic* isomerization on the graphene surface.

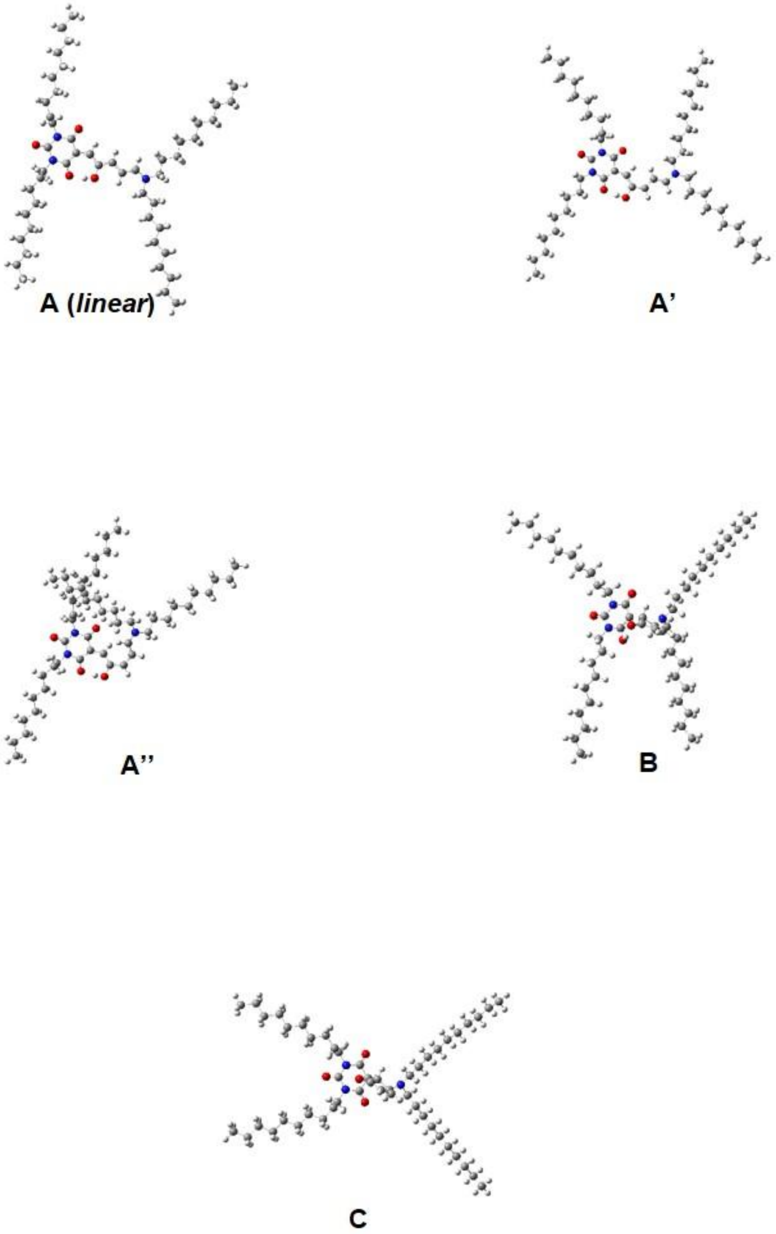

Figure S45. Calculated molecular structures of the 5 intermediates of **DASA-10C** during the *linear*-to-*cyclic* isomerization without graphene.

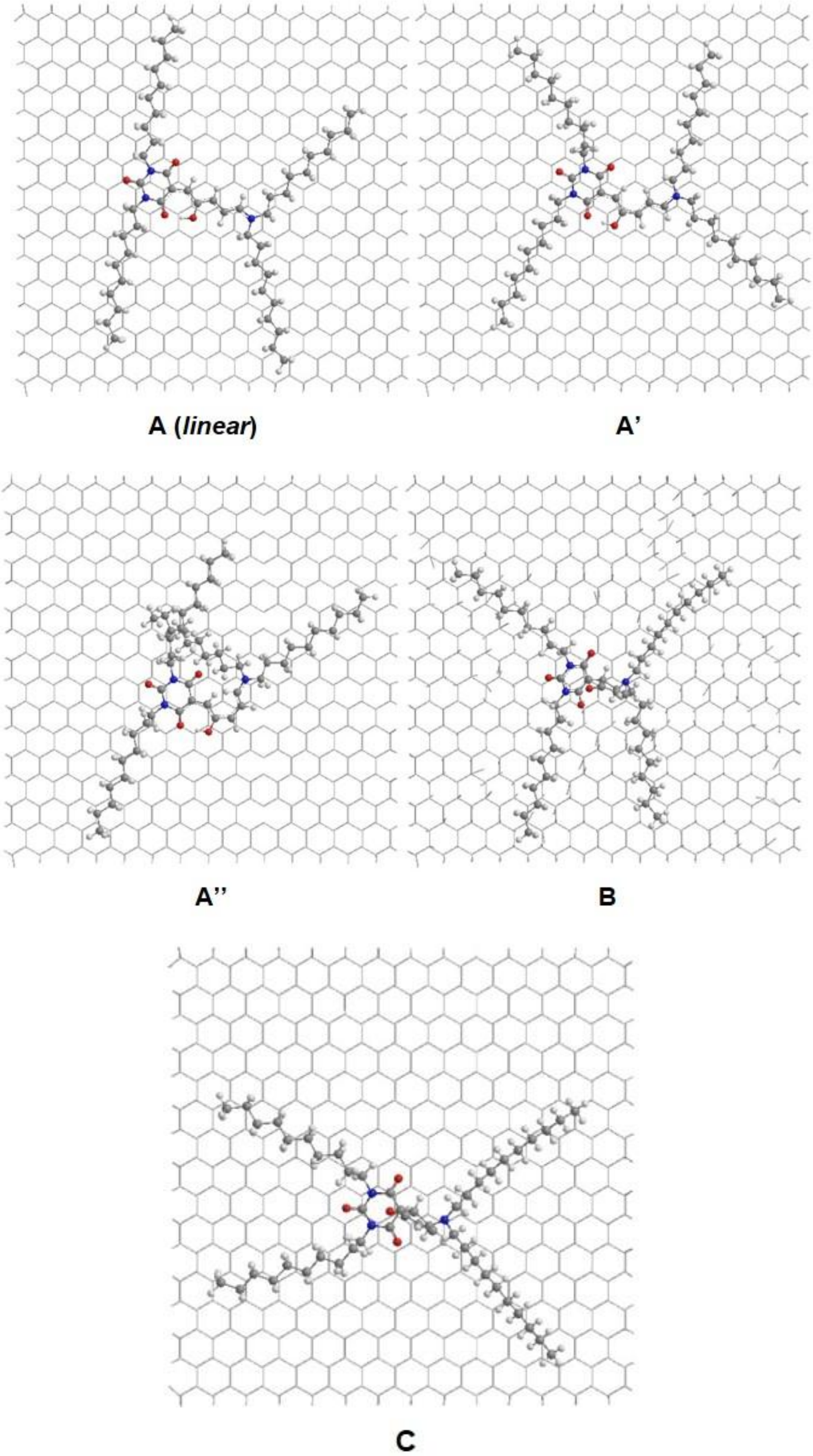

Figure S46. Calculated molecular structures of the 5 intermediates of **DASA-10C** during the *linear*-to-*cyclic* isomerization on the graphene surface.

Moreover, the mechanism of switching the I_{ds} of 2D logic-in-memory devices by *linear-cyclic* isomerization of DASAs was studied via DFT calculation. DASAs with 2-armed carbon spacers (n=11, termed as **DASA-12C-2arm**) immobilized on a single layer graphene were modeled (**Scheme S6**). The highest occupied molecular orbital (HOMO) of both *linear* and *cyclic* **DASA-12C-2arm** with graphene localizes on the photoswitches, while the lowest unoccupied molecular orbital (LUMO) localizes on graphene (**Figure S47-S48**). This means electron transition occurs from **DASA-12C-2arm** to graphene. The LUMO-HOMO energy gaps were calculated to be 0.28 and 0.57 eV for *linear* and *cyclic* **DASA-12C-2arm** with graphene, indicating the electronic conductivity is higher between *linear* **DASA-12C-2arm** and graphene. The energy gap difference was probably originated from the geometry of **DASA-12C-2arm** on the graphene surface and the intrinsic electronic structure. However, the interfaces are not considered during the calculation, which might be the main reason for the photomodulation of 2D logic-in-memory devices.

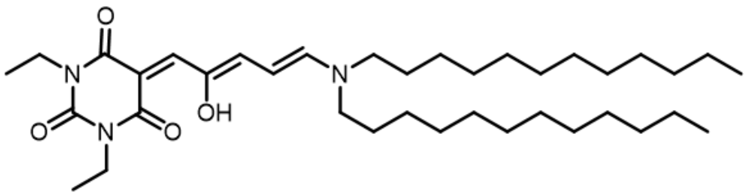

Scheme S6. Schematic illustration of the chemical structure of **DASA-12C-2arm**.

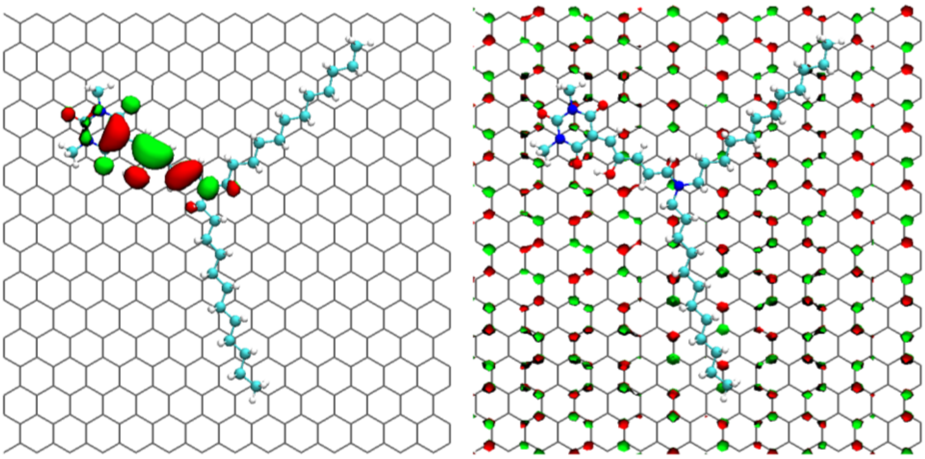

Figure S47. Calculated HOMO and LUMO distribution for *linear* **DASA-12C-2arm**

with graphene.

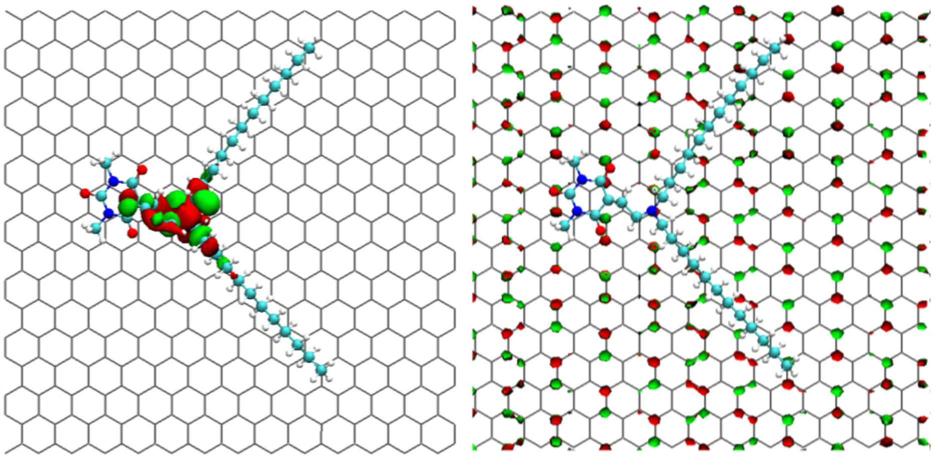

Figure S48. Calculated HOMO and LUMO distribution for *cyclic* **DASA-12C-2arm** with graphene.

9. NMR spectra of new chemicals

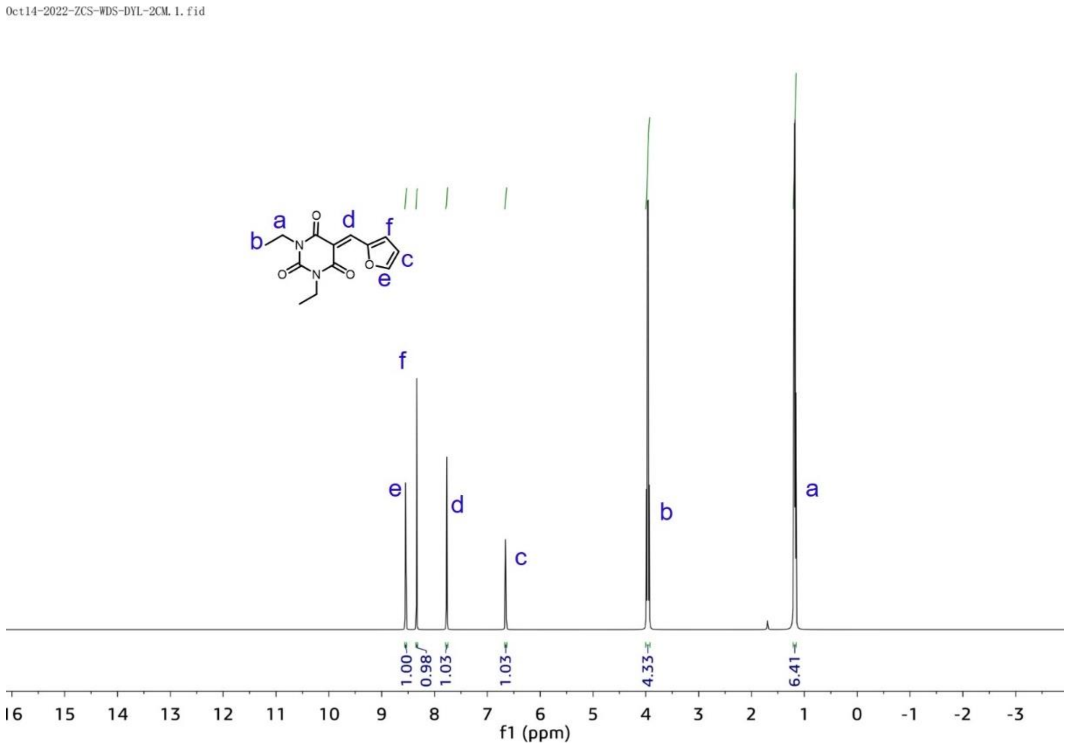

Figure S49. ^{1}H NMR spectrum (400 MHz at 298 K) of **compound 2d** in $CDCl_3$.

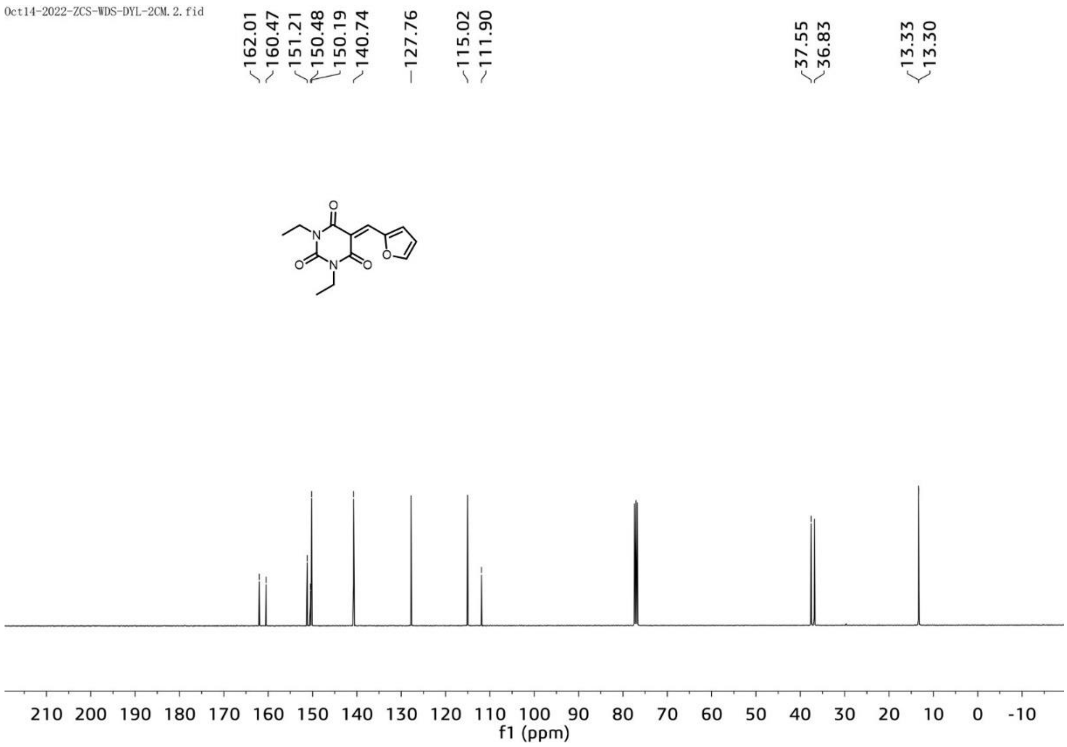

Figure S50. ^{13}C NMR spectrum (101 MHz at 298 K) of **compound 2d** in $CDCl_3$.

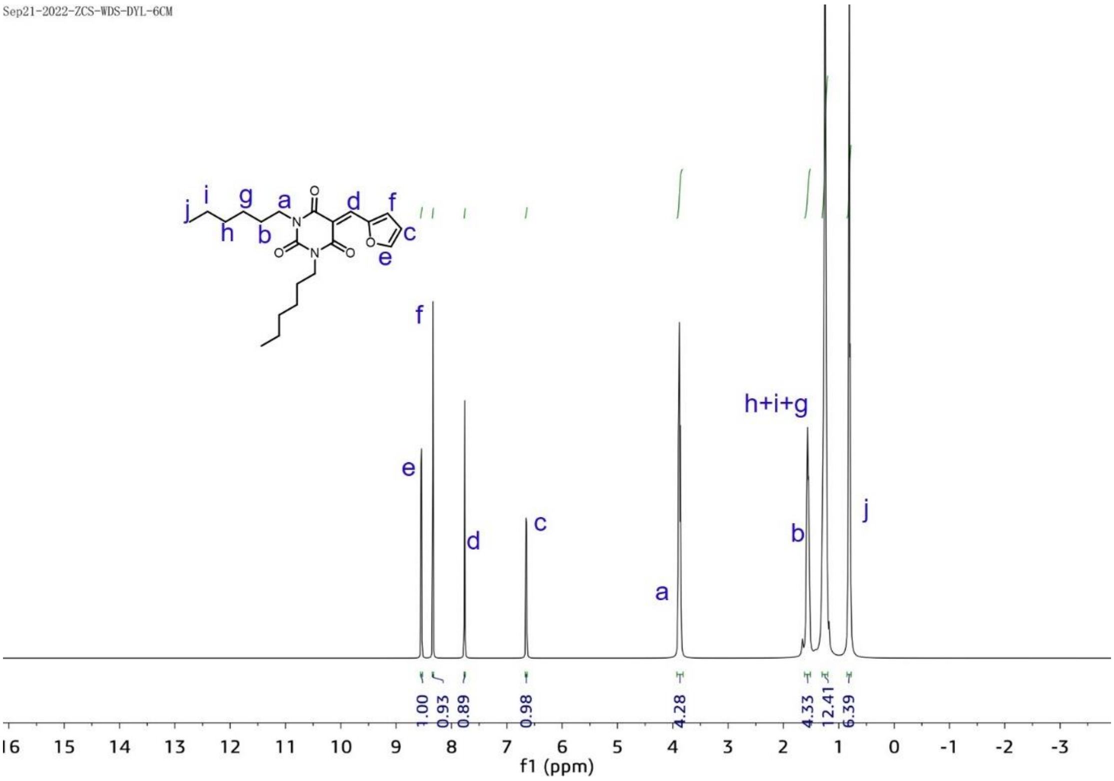

Figure S51. ^{1}H NMR spectrum (400 MHz at 298 K) of **compound 2c’** in $CDCl_3$.

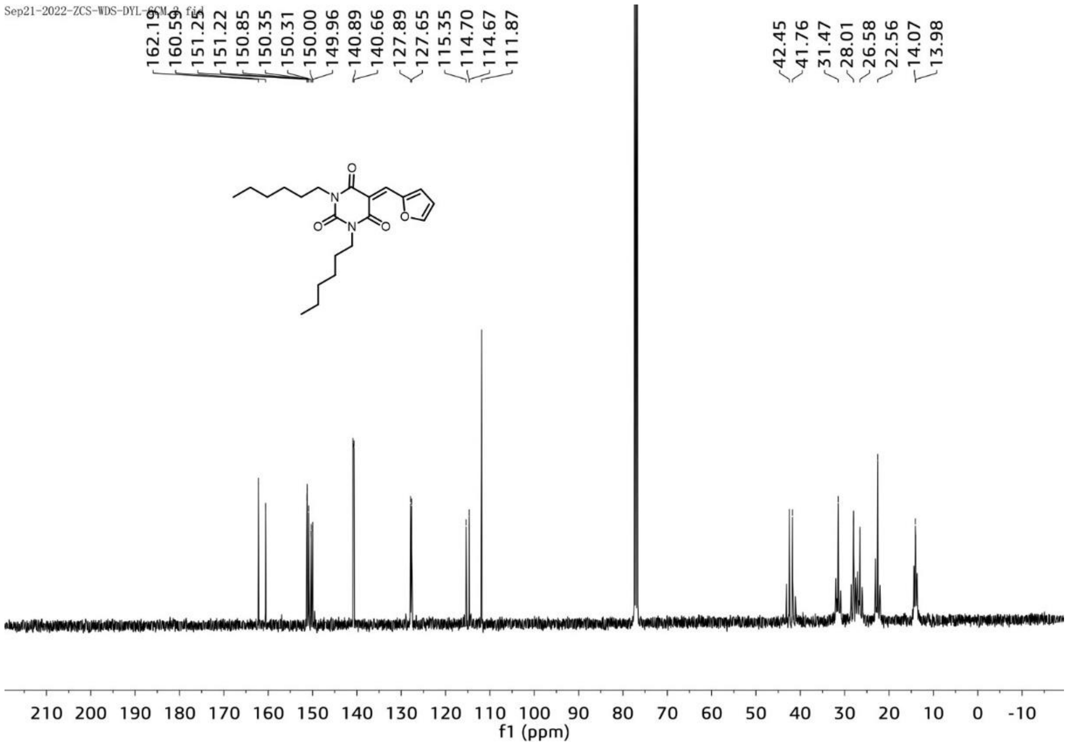

Figure S52. ^{13}C NMR spectrum (101 MHz at 298 K) of **compound 2c’** in $CDCl_3$.

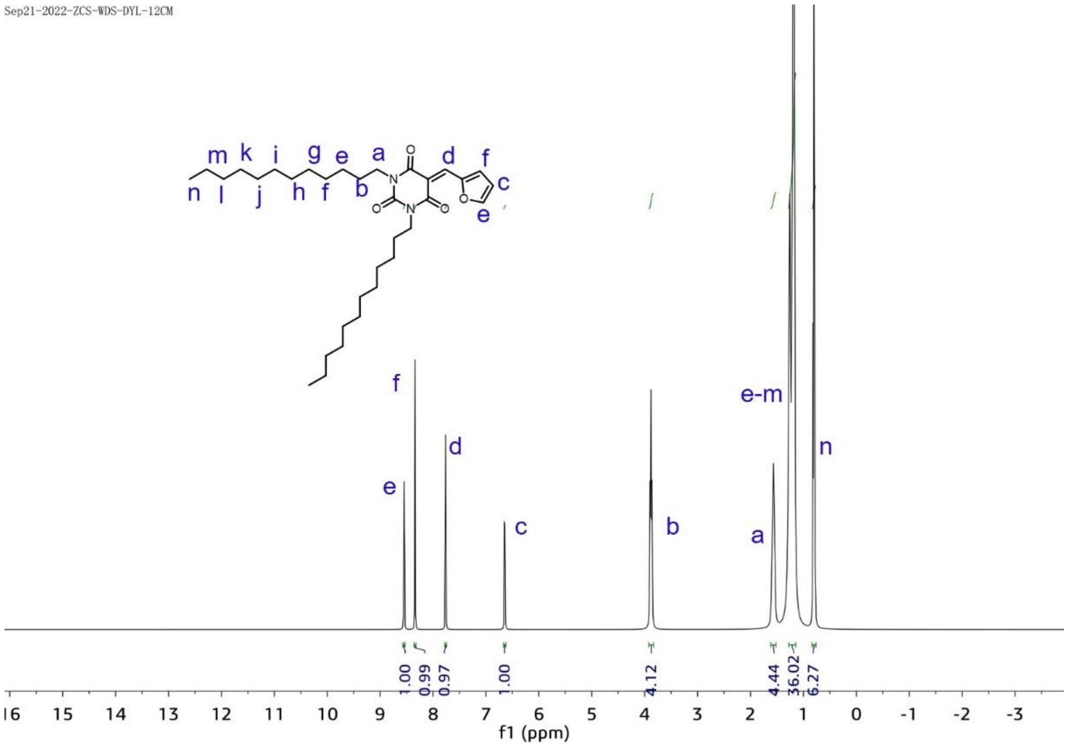

Figure S53. ^{1}H NMR spectrum (400 MHz at 298 K) of **compound 1d’** in $CDCl_3$.

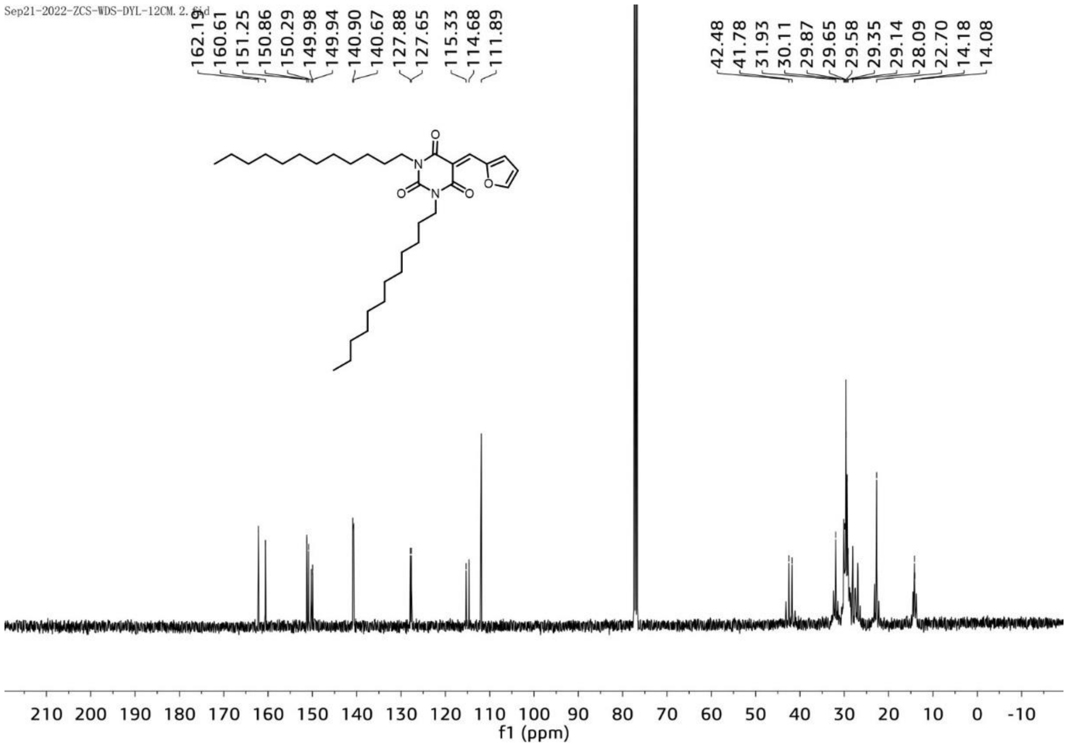

Figure S54. ^{13}C NMR spectrum (101 MHz at 298 K) of **compound 1d’** in $CDCl_3$.

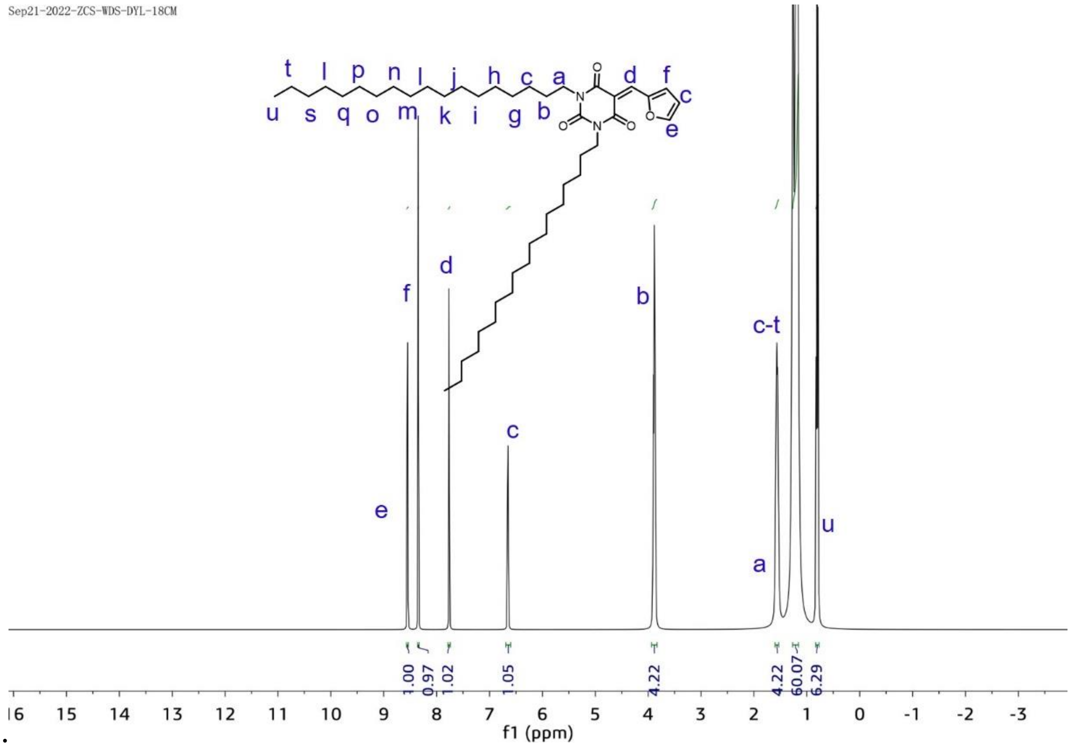

Figure S55. ^{1}H NMR spectrum (400 MHz at 298 K) of **compound 2h’** in $CDCl_3$.

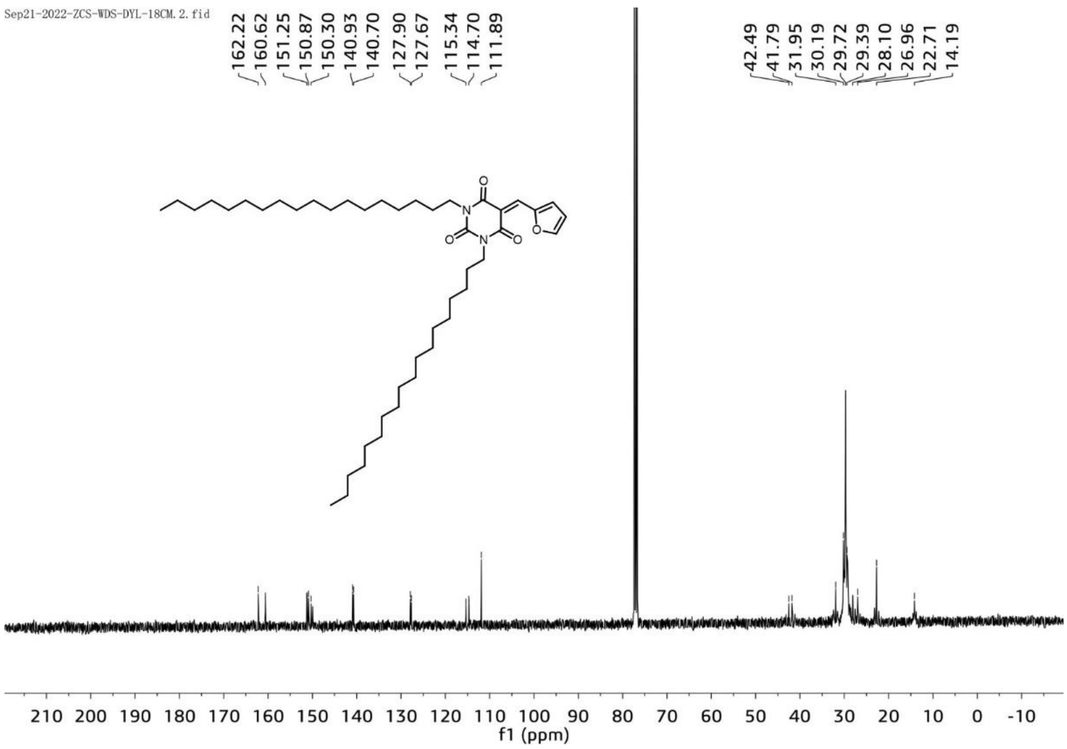

Figure S56. ^{13}C NMR spectrum (101 MHz at 298 K) of **compound 2h'** in $CDCl_3$.

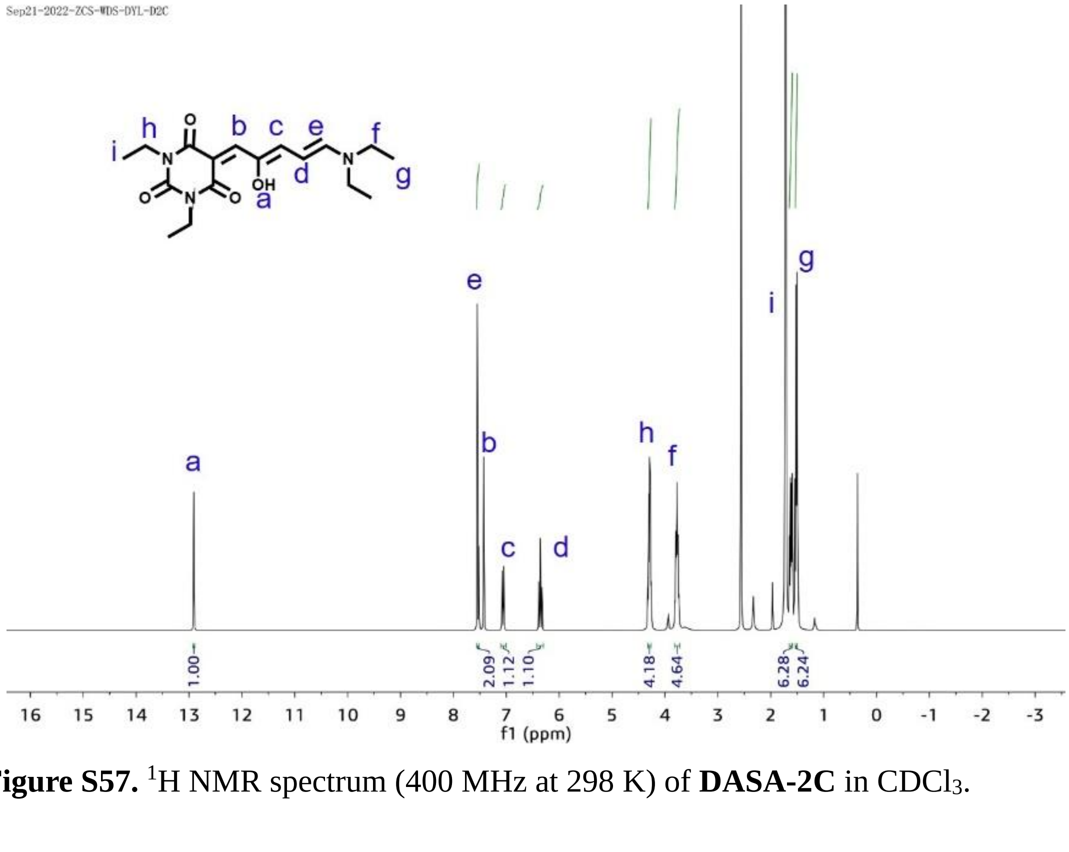

Figure S57. ^{1}H NMR spectrum (400 MHz at 298 K) of **DASA-2C** in $CDCl_3$.

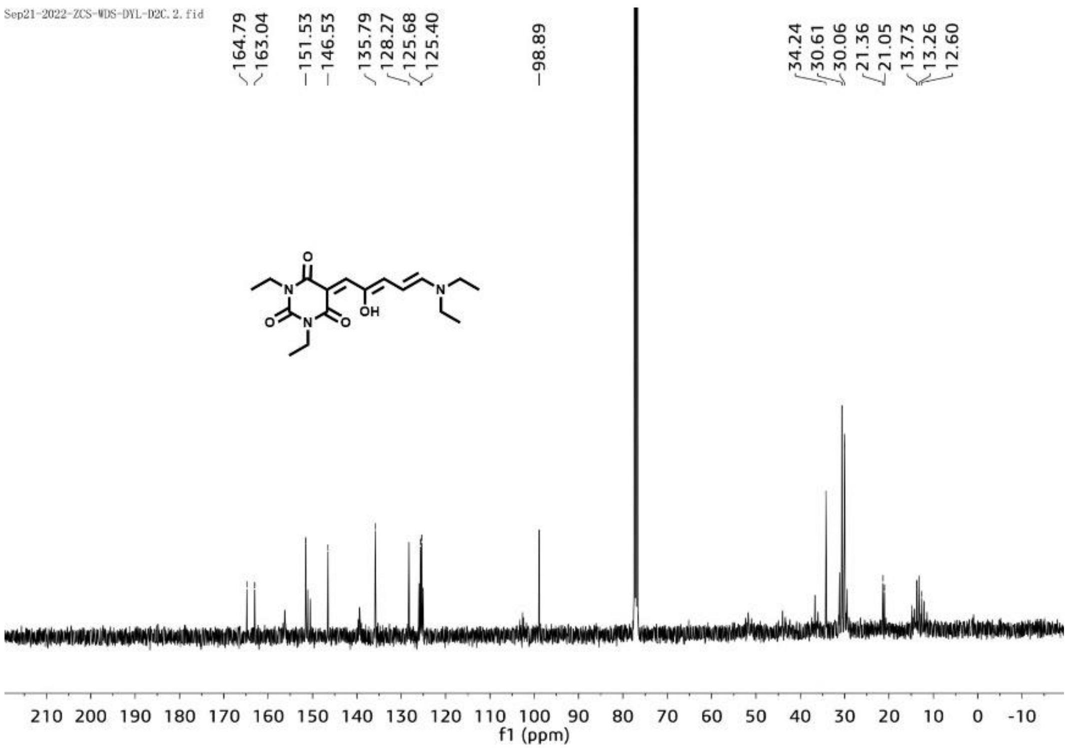

Figure S58. ^{13}C NMR spectrum (101 MHz at 298 K) of **DASA-2C** in $CDCl_3$

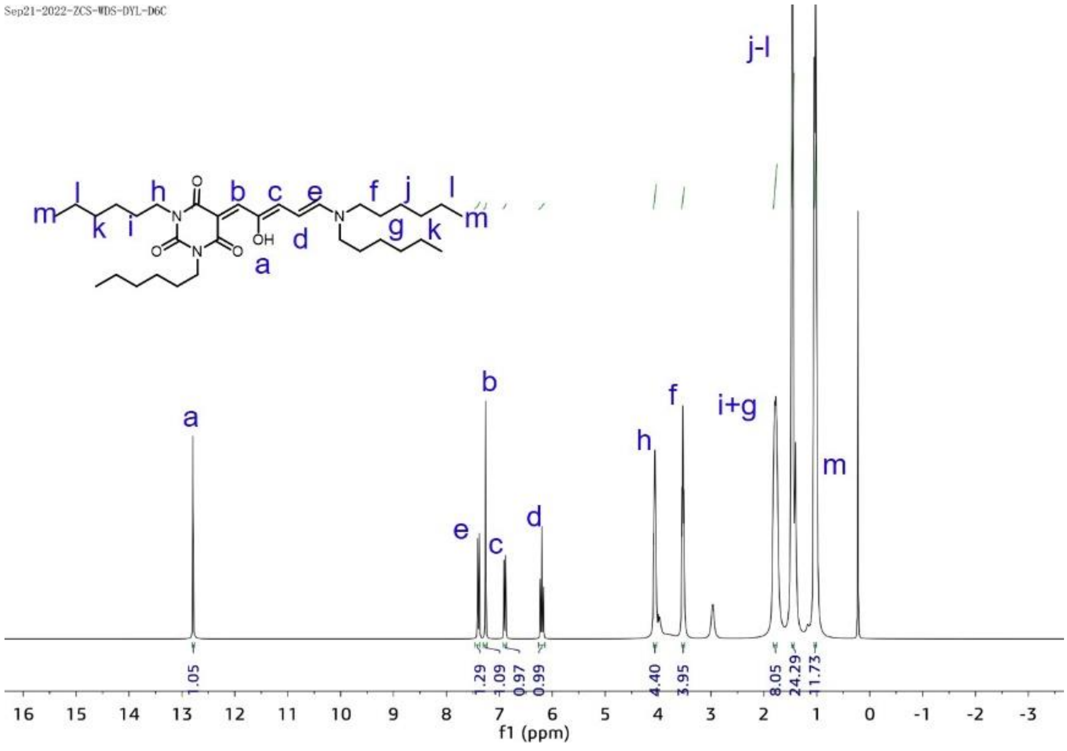

Figure S59. ^{1}H NMR spectrum (400 MHz at 298 K) of **DASA-6C** in $CDCl_3$.

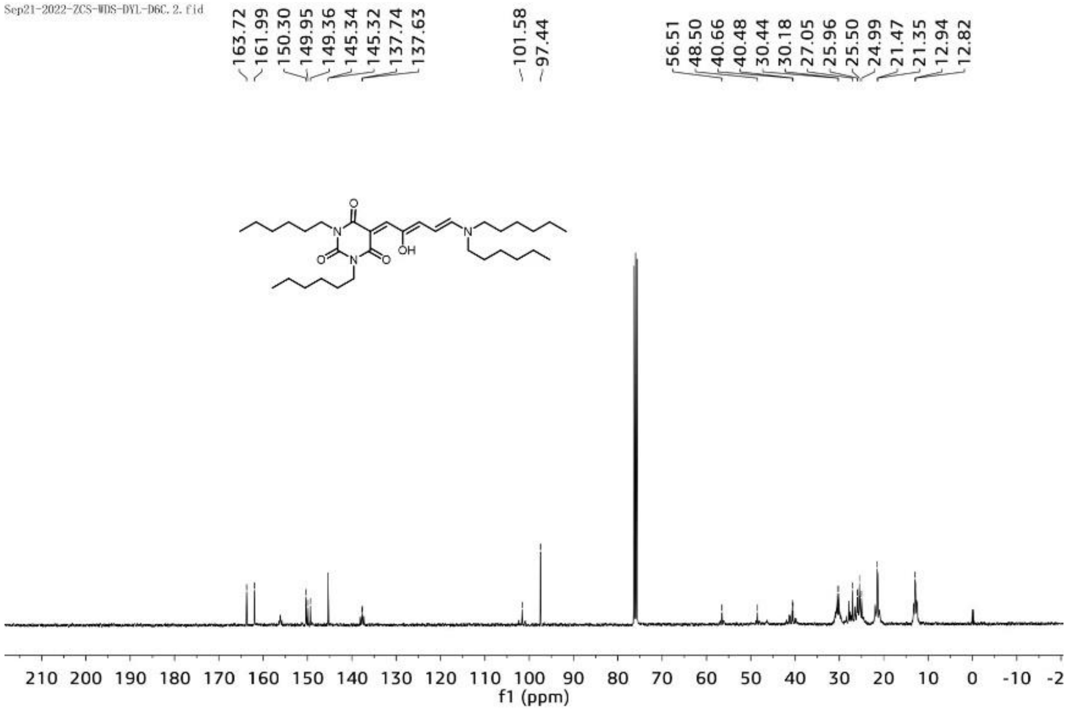

Figure S60. ^{13}C NMR spectrum (101 MHz at 298 K) of **DASA-6C** in $CDCl_3$.

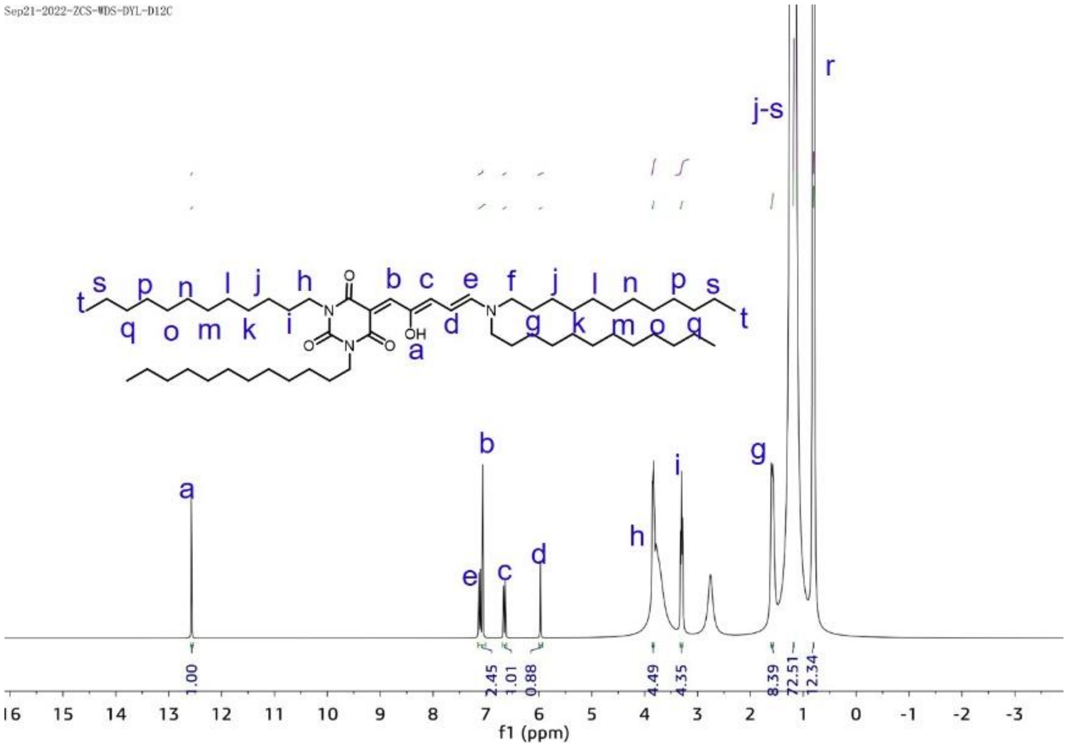

Figure S61. ^{1}H NMR spectrum (400 MHz at 298 K) of **DASA-12C** in $CDCl_3$.

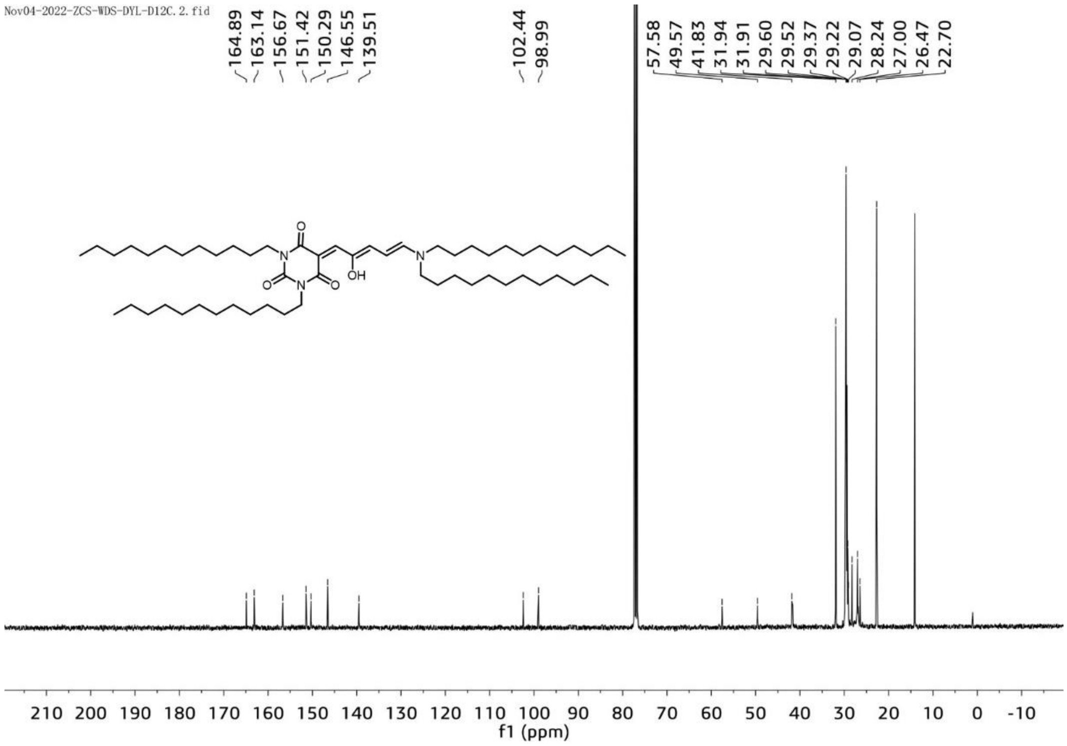

Figure S62. ^{13}C NMR spectrum (101 MHz at 298 K) of **DASA-12C** in $CDCl_3$

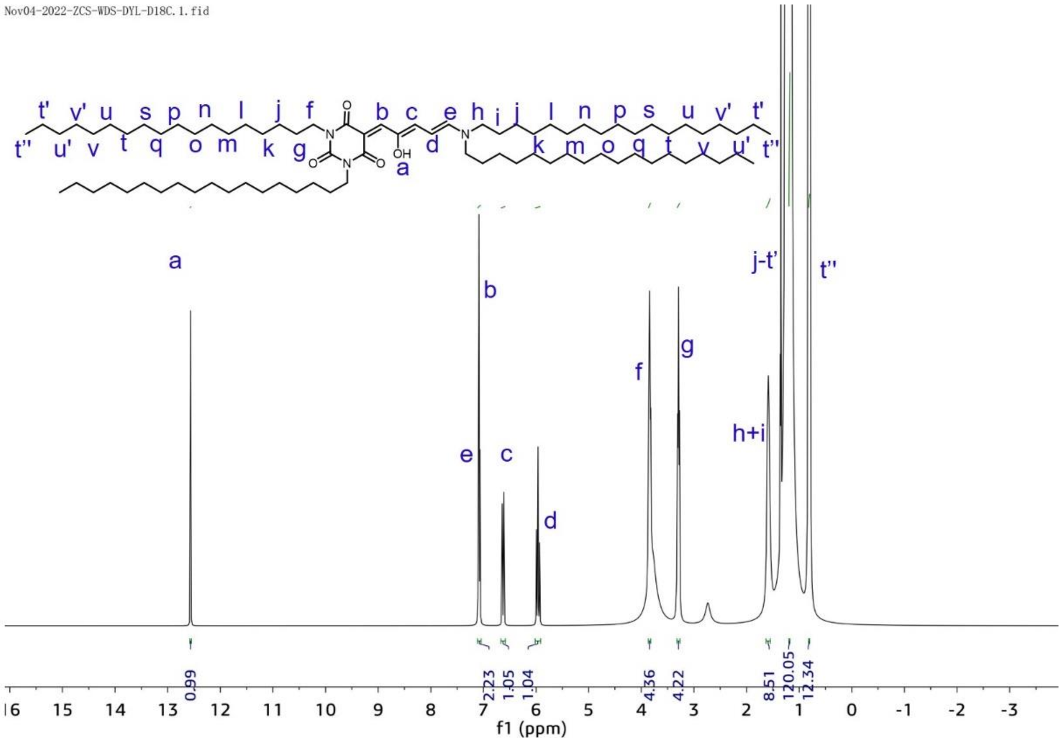

Figure S63. ^{1}H NMR spectrum (400 MHz at 298 K) of **DASA-18C** in $CDCl_3$.

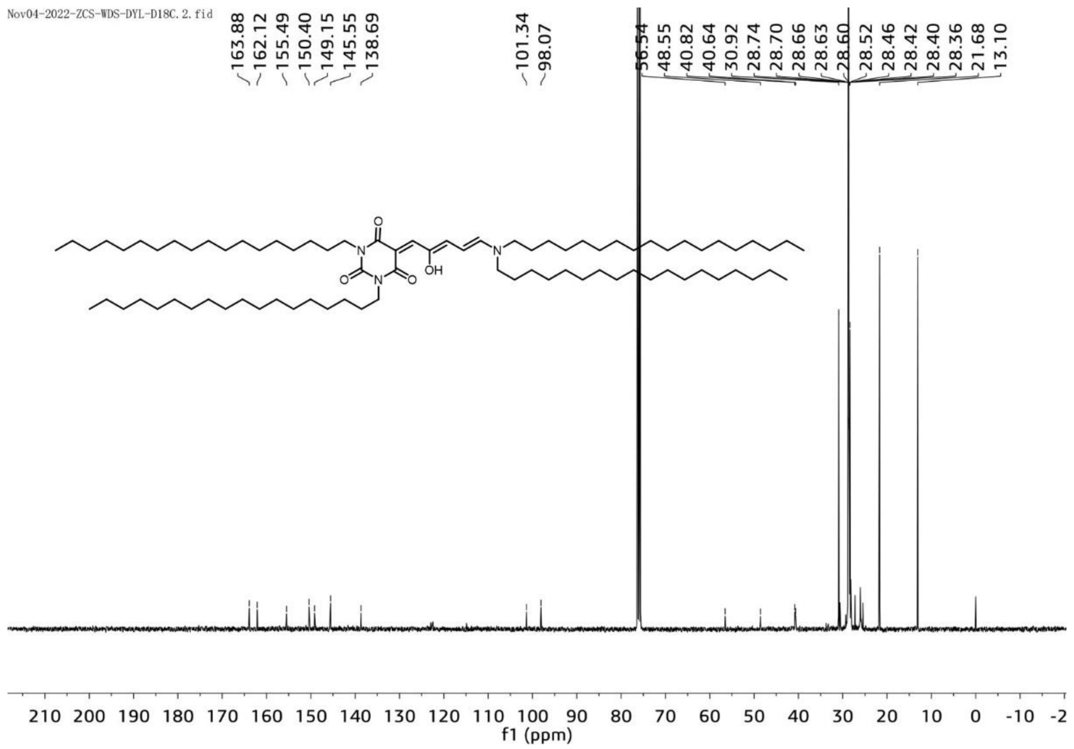

Figure S64. ^{13}C NMR spectrum (101 MHz at 298 K) of **DASA-18C** in $CDCl_3$

10. Reference

[1] Hutter, J.; Iannuzzi, M.; Schiffmann, F.; VandeVondele, J, *WIREs Comput. Mol. Sci.* **2014**, *4*, 15–25.

[2] Lippert, G.; Hutter, J.; Parrinello, M, *Mol. Phys*. **1997**, *92*, 477–487.

[3] Vandevondele, J.; Krack, M.; Mohamed, F.; Parrinello, M.; Chassaing, T.; Hutter, J, *Comput. Phys. Commun.* **2005**, *167*, 103–128.

[4] Perdew, J. P.; Burke, K.; Ernzerhof, M, *Phys. Rev. Lett.* **1996**, *77*, 3865–3868.

[5] VandeVondele, J.; Hutter, J, *J. Chem. Phys.* **2007**, *127*, 114105.

[6] Goedecker, S.; Teter, M.; Hutter, *J. Phys. Rev. B.* **1996**, *54*, 1703–1710.

[7] Grimme, Stefan; Ehrlich, Stephan; Goerigk, Lars, *J. Comput. Chem.* **2011**, *32*, 1456-1465.

[8] S. Helmy, F. A. Leibfarth, S. Oh, J. E. Poelma, C. J. Hawker, J. Read de Alaniz, *J. Am. Chem. Soc.* **2014**, *136*, 8169-8172

[9] Butt, H. J.; Cappella, B.; Kappl, M, *Surf. Sci. Rep.* **2005,** *59*, 1-152.

[10] Butt, H. J.; Jaschke, M, *Nanotechnology.* **1995,** *6*, 1.

[11] Johnson, K. L.; Kendall, K.; Roberts, *A. D. Proc. R. Soc. London.* **1971**, *324*, 301-313.

[12] Liu, H.; Fujinami, S.; Wang, D.; Nakajima, K.; Nishi, T, *Polymer Morphology: Principles*, *Characterization, and Processing*, John Wiley & Sons. **2016**, 317-334.
